# Mitochondrial transporter expression patterns distinguish tumor from normal tissue and identify cancer subtypes with different survival and metabolism

**DOI:** 10.1038/s41598-022-21411-0

**Published:** 2022-10-11

**Authors:** Hartmut Wohlrab, Sabina Signoretti, Lucia E. Rameh, Derrick K. DeConti, Steen H. Hansen

**Affiliations:** 1 Department of Biological Chemistry and Molecular Pharmacology, Harvard Medical School, 240 Longwood Avenue, Boston, MA 02115 USA; 2 GI Cell Biology Research Laboratory, Boston Children’s Hospital, 300 Longwood Ave, Boston, MA 02115 USA; 3 Department of Pathology, Brigham and Women’s Hospital, 75 Francis Street, Boston, MA 02115 USA; 4 Harvard Medical School, Boston, USA; 5 Department of Biochemistry, School of Medicine, Vanderbilt University, 2209 Garland Ave, Nashville, TN 37240 USA; 6 Quantitative Biomedical Research Center, Department of Biostatistics, Harvard T.H. Chan School of Public Health, 655 Huntington Ave, Boston, MA 02115 USA

**Keywords:** Biotechnology, Cancer, Computational biology and bioinformatics

## Abstract

Transporters of the inner mitochondrial membrane are essential to metabolism. We demonstrate that metabolism as represented by expression of genes encoding SLC25 transporters differentiates human cancers. Tumor to normal tissue expression ratios for clear cell renal cell carcinoma, colon adenocarcinoma, lung adenocarcinoma and breast invasive carcinoma were found to be highly significant. Affinity propagation trained on SLC25 gene expression patterns from 19 human cancer types (6825 TCGA samples) and normal tissues (2322 GTEx samples) was used to generate clusters. They differentiate cancers from normal tissues. They also indicate cancer subtypes with survivals distinct from the total patient population of the cancer type. Probing the kidney, colon, lung, and breast cancer clusters, subtype pairs of cancers were identified with distinct prognoses and differing in expression of protein coding genes from among 2080 metabolic enzymes assayed. We demonstrate that SLC25 expression clusters facilitate the identification of the tissue-of-origin, essential to efficacy of most cancer therapies, of CUPs (cancer-unknown-primary) known to have poor prognoses. Different cancer types within a single cluster have similar metabolic patterns and this raises the possibility that such cancers may respond similarly to existing and new anti-cancer therapies.

## Introduction

Substantial resources are vested into targeting metabolisms of cancer. Mitochondria have been the focus of extensive studies in normal physiology but have received much less attention in cancer. They contribute to essential cellular metabolisms beyond oxidative phosphorylation of ADP. Such contributions entail purine and pyrimidine biosynthesis (C1 metabolism), fatty acid biosynthesis and β-oxidation, steroid biosynthesis, the urea cycle, amino acid metabolism, oxidative carbohydrate metabolism, reductive carboxylation, the generation of reactive oxygen species (ROS), apoptosis^1^ and other aspects of cellular metabolism and signaling. SLC25 transporters are key to these processes, because they facilitate the transport of metabolites between cytosol and mitochondrial matrix located enzymes. These uni/sym/antiporters rigorously control transport across the inner mitochondrial membrane to preserve the mitochondrial electrochemical potential essential for oxidative phosphorylation. Cancer metabolism differs substantially from normal tissue^2–4^. Yet, in spite of their pivotal role in cell metabolism, a thorough investigation of the gene expression of SLC25 transporters as an interdependent group of facilitators of the many metabolic pathways is lacking.

FDA has approved tissue-agnostic agents [pembrolizumab (Keytruda), larotrectinib (Vitrakvi), entrectinib (Rozlytrek)] that were expected to dominate cancer therapies^5^. Unfortunately, tissue-agnostic agents, while very effective, are useful for only a very small percentage of cancers. The metabolic network of a tumor is more similar to the normal tissue from which the cancer arose than it is to the metabolic network of a tumor arising from another tissue^6^. Most cancer therapies thus remain tissue specific^7–9^. Tissue-specificity is also highly important for the promising cancer immunotherapies^10^. The importance of tissue specificity is furthermore evident from cancer of unknown primary (CUP) that is generally associated with a very poor prognosis. Some progress has been made in the identification of tissue of origin of some of such cancers^11–14^. However, further advances are much needed.

Here we demonstrate that the SLC25 gene expression patterns distinguish cancer from normal tissue. Taking advantage of affinity propagation, tissue types have been separated into 44 clusters. Most of the 19 human cancer types investigated have been separated from their respective normal tissues. Additionally, we demonstrate that (a) cancers of a single type but located in different clusters can be clinically separated on the basis of different survival times and the expression of a select group of metabolic enzyme genes and (b) some clusters contain several cancer types, which may respond to the same cancer therapeutics given their highly similar mitochondria-related metabolisms. Finally, the treatment of CUP cancers may be facilitated since tissue-of-origin can often be established from the cluster in which the tissue is located. Our approach is new, because it defines human cancer on the basis of metabolic patterns as indicated by the expression of SLC25 genes.

## Methods

### Gene transcript data

The Cancer Genome Atlas (TCGA) (https://portal.gdc.cancer.gov) was searched for single patient cancer and uninvolved tissue expression data for clear cell renal cell carcinoma (68 patients, ccRCC, TCGA-KIRC), colon adenocarcinoma (41 patients, CRC, TCGA-COAD), lung adenocarcinoma (54 patients, TCGA-LUAD), and breast invasive carcinoma (110 patients, TCGA-BRCA). The ENSG identifiers of the 1158 genes of the MitoCarta 2.0^15^ were obtained from https://www.broadinstitute.org. Twelve MitoCarta human genes were absent from the TCGA files. ENSG00000106246 (PTCD1, ATP5J2-PTCD1) and ENSG00000223572 (CKMT1A, CKMT1B) are each listed twice in the MitoCarta. MitoCarta refers here only to 1131 nuclear mitochondrial protein genes. The 13 mitochondrial DNA protein genes are not included. MitoCarta 3.0 data^16^ are not expected to significantly affect our results and conclusions.

### Total RNA

Total RNA was prepared with the Qiagen RNeasy Plus Micro Kit. Its concentration and purity assessed spectrophotometrically with a NanoDrop spectrophotometer. Tissues were processed with the Qiagen TissueLyser II. Cancer cell line (CCL) cells were processed with QIAshredder. Total RNA of NCI60 human CCLs (Caki-1, 786-O, A498, RXF-393, HCT-116, HT-29) was obtained from the Developmental Therapeutics Program NCI. ccRCC tissues and total RNA were obtained from the Brigham and Women’s Hospital. CRC tissues and total RNA were provided by Dr. Bert Vogelstein (Hereditary Colorectal Cancer Registry, Johns Hopkins Medicine).

### qRT-PCR

Reverse transcriptase (QuantiTect Reverse Transcription Kit) and QuantiFast SYBR Green PCR Kit were purchased from Qiagen. Primers (Supplementary Table S1) were purchased from Qiagen (QuantiTect Primer Assay) and Integrated DNA Technologies (Prime Time qPCR Primers).

All qRT-PCR reactions were initiated with the same amount of cDNA (total RNA) as total tissue RNA levels are generally tissue type independent^17^. Transcript concentrations were based on 21.63 (kidney) and 22.90 (colon) reference amplification cycles. The 20 µl reactions were carried out in 96 well plates in a BioRad MJ Opticon or QuantStudio 3: 5 min at 95 °C then 40 cycles of 95 °C (10 s) and 60 °C (30 s). Temperature increase (50 °C to 95 °C with reads every 0.2 °C) yielded melting curve of amplified fragments. All reactions were run at least twice and the mean with the two data points is indicated in the plots.

### SLC25 knockdown in Caki-1 cells

Lenti-293 T cells were from lab stock. All cells were cultured in DMEM supplemented with 10% FCS at 37 °C in 5% CO_2_. Lentiviral vectors encoding tetracycline inducible shRNAs targeting SLC25A22, SLC25A32, or SLC25A43 are shown in Supplementary Table S2. 2 × 10^6^ Lenti-293 T cells were transfected with 2 µg of lentiviral PAX2 packaging vector and VSV-G envelope encoding vector (Supplementary Table S2) along with appropriate shRNA expression vector using Fugene 6. Cell culture medium was replenished 24 h later and 48 h later supernatants were harvested and used to transduce approximately 5 × 10^5^ Caki-1 cells in the presence of polybrene. An additional 48 h later, the Caki-1 cells were subjected to selection with 2.5 µg/ml puromycin for 10 days. To induce knockdown of SLC25 transporters, pooled populations of Caki-1 cells were incubated 3 days in the presence of 2 µg/ml doxycycline.

### Unsupervised clustering of cancer samples (affinity propagation)

Affinity Propagation (AP) and SLC25 genes expressions were used in the identification of cancers and normal tissues. It is an unsupervised classification model that clusters samples by similarity of the expression of the SLC25 genes (https://pubmed.ncbi.nlm.nih.gov/17218491/). A TCGA data set (6825 samples) was combined with a GTEx data set (2322 samples). We used the 41 SLC25 genes set (L26 and S24 combined) (see “L10 and S10 SLC25 gene sets”): A1, A15, A4, A5, A6, A7, A8, A9, A10, A13, A14, A30, A16, A43, A22, A18, A19, A20, A21, A23, A24, A25, A41, A27, A28, A37, A29, A32, A33, A36, A34, A35, A38, A39, A40, A42, A44, A45, A47, A48, A50. The unsupervised clustering algorithm in AP identified clusters of samples based on the expression of the 41 genes. The gene expression was z-score normalized by sample. That is, each sample was normalized to the mean and standard deviation of the gene expression of those 41 genes. We tuned the AP algorithm parameters to 44 clusters. The parameters eventually settled on, attempting to minimize the number of clusters, were: preference (− 1000), affinity (Euclidean), seed (202000902). This resulted in 44 distinct clusters being identified.

### Supervised learning for assignment of AP derived cancer types

The tested classification models: Support Vector Machines (SVM), K-Nearest Neighbors (KNN), Random Forest (RF), Gradient Boosted Trees (GBT), and Gaussian Naive Bayes (GNB). The first step was to split the data set (z-score normalized expression values). First, we filtered for only the TCGA samples. Second, we removed any of the unsupervised clusters that had less than five samples in it. Finally, we randomly split the data by 60%/40%. 60% went to the training data and 40% went to the test data set. The test data set was only used for the final test of the final optimized model to determine how well the actual classification model performs. This was to ensure that the classification model was not overfitted to the data. Additionally, the test-train split was stratified, so that 60/40 split is approximately applied to all labels, rather than risk unbalanced labels from a purely random assignment.

From the train data, we performed a nested cross validation assay. This essentially tests all the various parameter tuning plus cross validation (cross validation is where the train data is further split into a random assortment of test-train splits) to determine an unbiases estimate of the performance of the classification models. Over multiple tests, the results of the F1 scores (TP/(TP + 1/2 * (FP + FN))) for the various tests (Supplementary Table S3) revealed that SVM with a non-linear kernel would be the best model for classification. The next step was to optimize the parameters for SVM to best tune the model for the data. We performed a grid-based search for the best parameters and the result was: kernel: rbf; gamma: 1e−5; C: 5000.

The results of the test against the reserved test data set were as follows: Precision: TP/(TP + FP); Recall: TP/(TP + FN); F1-score: TP/(TP + 1/2 * (FP + FN)); Support: number of samples. The macro average (avg) is the mean of all the scores, while the weighted avg is the mean score adjusted by the support (Supplementary Table S4).

In addition, the results were tested against the GTEx data set that was filtered from use in the training data (Supplementary Table S5).

### Quantification and statistical analysis

Statistically significant differences between two arrays of data (p-values) were established with the Students t-test (Excel T.TEST), two-tailed distribution and unequal (unknown) variances. Patient survival distributions, as illustrated by Kaplan–Meier plots, were subjected to pair-wise log-rank testing by comparing subtypes to all patients diagnosed with a given form of cancer.

## Results

### Transcriptome of mitochondria in human cancer

**Figure 1:**
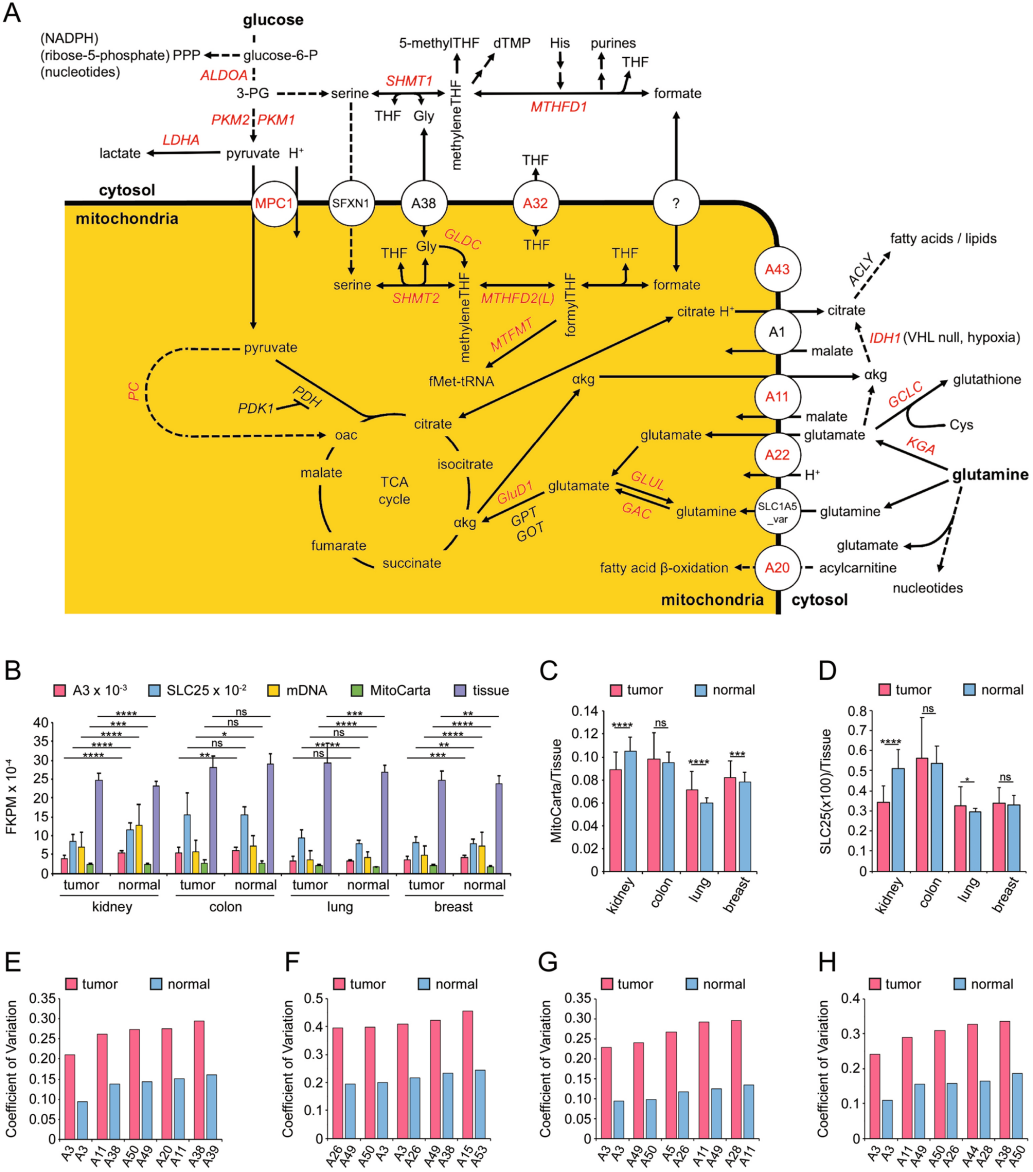
Mitochondrial SLC25 transporters. (**A**) Some mitochondrial transporters and their substrates. Transporters and enzymes assayed by RT-qPCR are shown in red (Supplementary Figs. S3–S5). (**B**) Gene expression of tumor and normal tissue. ‘A3’ denotes the SLC25A3 phosphate transport protein (PTP). ‘SLC25’ is the sum of FKPMs of all SLC25 genes. ‘mDNA’ is the sum of FPKMs of the 13 mitochondrial DNA protein coding genes. ‘Tissue’ refers to the sum of FKPMs of all protein coding genes on nuclear DNA. (**C**) Ratio of sum of FPKMs of MitoCarta over tissue genes in tumor and normal tissue from KIRC, COAD, LUAD and BRCA. (**D**) FPKM ratio of SLC25 over tissue genes from KIRC, COAD, LUAD, and BRCA and normal tissue. Error bar standard deviation. Only transporters with smallest coefficient of variation (standard deviation/mean) of transporter FPKM/sum of MitoCarta gene FPKMs from tumor and normal tissue for kidney [(**E**) KIRC)], colon [(**F**) COAD)], lung [(**G**) LUAD], and breast [(**H**) BRCA] are shown. Those of all SLC25 transporters are shown in Supplementary Fig. S1.

Mitochondria are essential to the cell. Their SLC25 transporters (Supplementary Table S6) shuttle metabolites from and into their matrix space (Fig. 1A). The expression (TCGA) of SLC25 transporter genes in four tumor types and their normal tissue was explored initially. We used 1131 of the MitoCarta genes (“Methods”)^15^ and refer to them as MitoCarta.

The expression of MitoCarta genes (TCGA) revealed major differences between normal and tumor tissue. Most significant, the sum of the FPKMs of the MitoCarta genes of ccRCC is 9.5% smaller than that of normal kidney tissue (Fig. 1B). Such FPKM decreases are also observed in sum of the thirteen mitochondrial DNA protein genes (45%), of the SLC25s (26%) and of the A3 phosphate transport protein (PTP)^18^ (26%). Figure 1C,D show that only in kidney is the ratio of the sum of MitoCarta FPKMs over the sum of all tissue gene FPKMs (MitoCarta/Tissue ratio) lower for tumor than for uninvolved tissue. This is in line with recently published data on the TCA cycle enzymes in kidney cancer^19^. The MitoCarta/Tissue ratio of colon tumor and uninvolved tissue is the same. For lung and breast this ratio is larger for tumor than for uninvolved tissue. Taken together, the MitoCarta/Tissue ratio exhibits tissue specificity in human cancer.

The A3 gene is of interest since the variation (coefficient of variation) of its FPKM over the sum of MitoCarta gene FPKMs among normal tissue samples (kidney, lung, breast) is the lowest (second or third lowest for colon) of all SLC25 transporter genes (Fig. 1E (kidney), F (colon), G (lung), H (breast)). This also holds true for the corresponding tumor tissues. However, for tumor tissue, the coefficient of variation is, as expected, significantly larger for all transporters (Fig. 1E–H). Only transporters with the smallest coefficient of variation are shown here to emphasize their high correlation with total nuclear mitochondrial gene expression. The coefficient of variation of all SLC25 transporters are shown in Supplementary Fig. S1. It should be noted that the coefficient of variation (standard deviation over the mean) is used here rather than standard deviation in order to be able to compare transporters with large differences in mean expression levels.

### The SLC25 family of mitochondrial transporters

**Figure 2:**
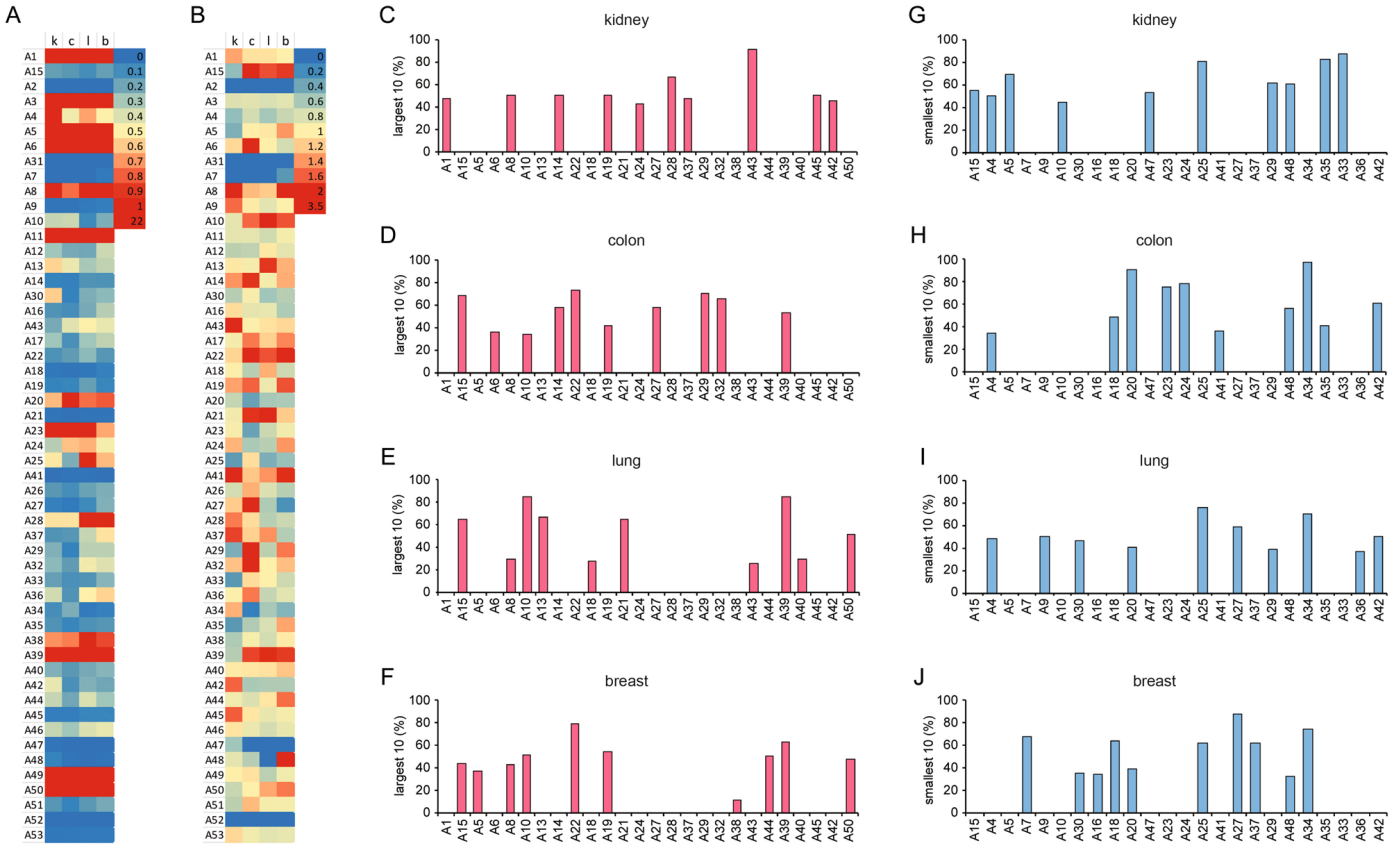
Expression of SLC25 genes in tumor and uninvolved tissue. (**A**) Heat map of uninvolved tissue FPKM ratios of single SLC25 over the sum of MitoCarta genes (FPKM SLC25Ax/ΣFPKM MitoCarta) from same person. ‘k’ (KIRC), ‘c’ (COAD), ‘l’ (LUAD), and ‘b’ (BRCA) with values shown × 10^3^. (**B**) SLC25Ax/ΣFPKM MitoCarta ratios for tumor over those of uninvolved tissue (same individual). The L10 transporters [(**C**) KIRC], [(**D**) COAD], [(**E**) LUAD], [(**F**) BRCA] and the S10 transporters [(**G**) KIRC], [(**H**) COAD], [(**I**) LUAD], [(**J**) BRCA]. The percentage of patients with the indicated L10 and S10 of the total number of patients probed is shown in (**C**–**J**). The transporters are arranged numerically as indicated in Table 1. Those transporters with sequence similarity are grouped together between the red lines.

The normalized mitochondrial SLC25 transporter expression levels (FPKM of individual SLC25 over sum of FPKMs of Mitocarta genes) in uninvolved tissue are very different (Fig. 2A) between kidney (k), colon (c), lung (l), and breast (b) tissue. Expression levels are largest for the two ADP/ATP transporters (A5, A6). Those for a few other transporters should be noted: A39 is largest for kidney (2.0) and smallest for breast (1.0) with colon and lung about the same (1.5); A32 is smallest for colon and largest for lung with breast larger than kidney; A43 is largest for lung and smallest for kidney with colon and breast approximately the same; A22 is very small for the four tissues (less than 0.25), yet largest for lung and smallest for kidney but similar for breast and colon.

The expression of the SLC25 genes in the cancers compared to normal tissues, i.e., ratio of normalized expression of transporter in cancer tissue over that in normal tissue (Fig. 2B), differs quite frequently from 1.0. It had been assumed that SLC25 gene expression levels are minimally affected by altered cellular metabolic conditions^20^. The cancer SLC25 gene expression difference from 1.0 (Fig. 2B), however, is often statistically highly significant (Table 1). For A20, essential for fatty acid oxidation, the expression in cancer is significantly below 1.0 in the four cancer types. In contrast, the expression of A19 is increased in all cancers except in lung where the change is insignificant. A15 (ornithine) expression is larger in all cancers except kidney where it is smaller. Taken together, the data suggest that expression of SLC25 genes and thus cancer metabolisms can be utilized to differentiate between tumor types, tumor and normal tissue, and to identify a cancer’s tissue of origin.

**Table 1:**
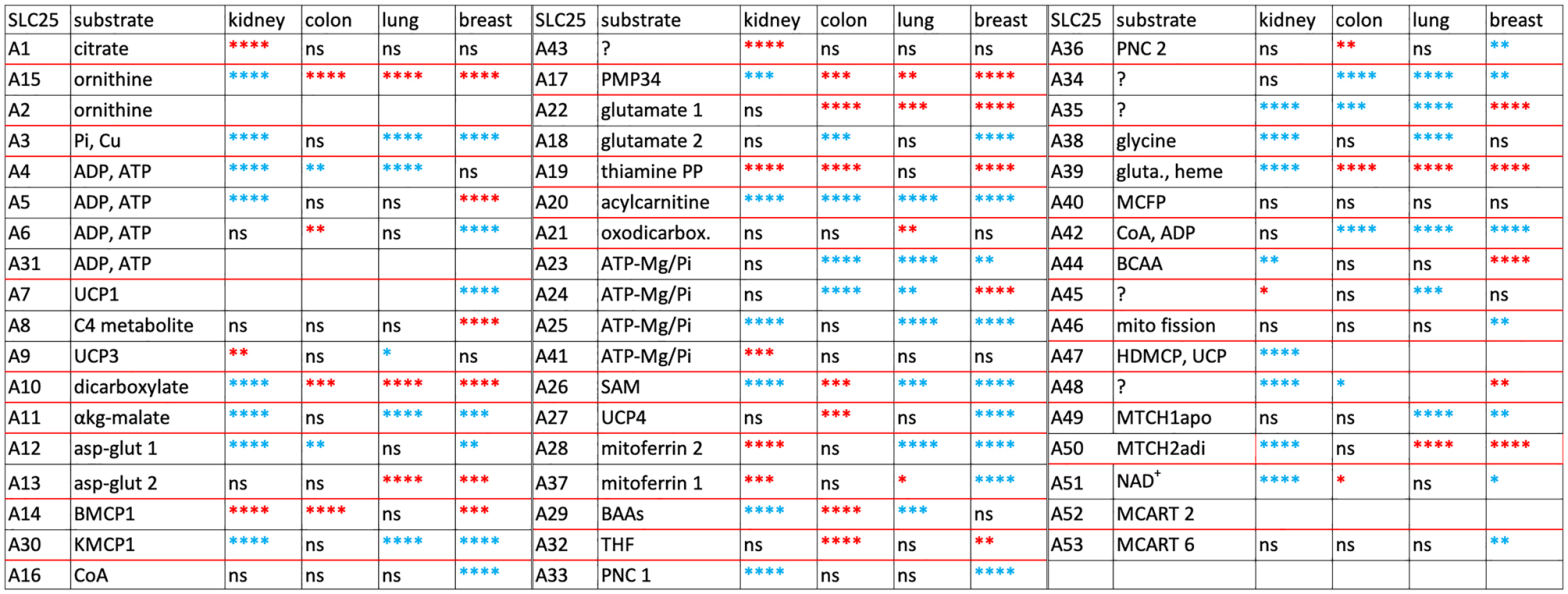
Significance of SLC25 transporter genes with induced and suppressed expression in tumors compared to normal tissues Expression (FPKM) ratio of SLC25 gene of cancer per MitoCarta sum of same patient compared to same gene, same ratio for normal tissue of same patient.

Transporters grouped between bold red lines have high sequence similarity, i.e., they have at least one transmembrane helix with similarity E-value of less than 1e-04. Substrate column lists transported substrate and/or name of transporter. UCP (uncoupling protein), BMCP1 (brain mitochondrial carrier protein 1), KMCP1 (kidney mitochondrial carrier protein 1), PMP34 (peroxisomal membrane protein 34, the only known SLC25 transporter expressed in peroxisomes and not in mitochondria), SAM (*S*-adenosyl methionine), BAA (basic amino acids), THF (tetrahydrofolate), PNC (pyrimidine nucleotide carrier), gluta. (glutathione), MCFP (mitochondrial carrier family protein), BCAA (branched-chain amino acids), HDMCP (hepatocellular carcinoma-down regulated mitochondrial carrier protein), MTCH1apo (apoptosis-related), MTCH2adi (adipocyte-related). p-Values of ratio data of Fig. 2B with red color indicating a ratio > 1.0 and blue color a ratio < 1.0. *(p < .05), **(p < .01), ***(p < .001), ****(p < .0001), ns (not significant). Kidney (KIRC)(n = 61), colon (COAD) (n = 41), lung (LUAD) (n = 54), breast (BRCA) (n = 110).

### L10 and S10 SLC25 gene sets

To facilitate a characterization of human cancers and their metabolisms with SLC25 expression patterns, we reduced the SLC25 genes to a small number and to those best identified with tissue specificity. Based on a transporter’s transcript ratio (TR) (FPKM ratio of cancer over normal tissue), the ten transporters with the largest and those with the smallest ratio were identified for each patient. From these transporters we selected those twenty (ten largest and ten smallest) most frequently present in a specific tumor type. These SLC25 transporters are the L10 and the S10 sets for each of the four cancer types (KIRC, COAD, LUAD, BRCA). The four sets of L10 and S10 transporters were pooled and yielded of 26 L10 (L26) and 24 S10 (S24) transporters. For each cancer type the percentage of patients with each of its L10 transporters within the L26 set is shown in Fig. 2C–F and similarly its S10 transporters within the S24 set in Fig. 2G–J. Some transporters (A5, A10, A15, A18, A24, A27, A29, A37, A42) are in both the L26 and the S24 sets. For instance, A15 is among the S10 for kidney, but also among the L10 for colon, lung and breast. The number of different transporters within a combined L26-S24 set is forty-one.

Next, to experimentally validate the utility of the L26-S24 set of SLC25 transporters to query publicly available data for cancer types, we determined SLC25 transcript ratios of seven ccRCC and seven CRC cancers and matched normal tissues from the same patients by RT-qPCR. CRC sample 3463-3T is from a liver metastasis. For its matched normal tissue, the average of matched uninvolved tissue data from the six primary colon cancers was used. The L10 and S10 of each of the fourteen tumors were identified. Expression arrays were generated (Supplementary Fig. S2) with columns of the L26 and S24 transporter sets and four rows (four cancer types) for each cancer sample. Thus, for each L10 transporter from an analyzed cancer that was identified with a column in Fig. 2C, a red square, defined by the transporter’s column and row "k" (kidney) of the analyzed cancer, was generated (Supplementary Fig. S2A). This process was repeated with the same transporter for rows "c" (colon), "l" (lung) and "b" (breast) (Supplementary Fig. S2A). This analysis was then extended to the other nine L10 transporters. Next, this process was repeated for the S10 transporters (Supplementary Fig. S2B). For all seven ccRCCs, the largest number of matches are in the "k" rows. The same type of analysis was carried out with the seven CRCs. Supplementary Figs. S2C,D show that the most matches are found in row "c" for CRC L10 and S10 transporters. Rows ‘l’ and ‘b’ showed fewer red squares. Taken together, the L26-S24 set of transporters (Supplementary Fig. S2A–D) is well suited for the identification of cancer types and cancer metabolisms.

### Metabolic importance of L10 genes

The L10 transporters are overexpressed in some but not all cancer types. Their metabolic role must be significant and should facilitate the identification of cell type and cancer type. We probed the metabolic significance of several L10 transporters using cancer cell lines (CCL). Tissue type identification of CCLs with L26-S24 transporter expression should also be possible. Supplementary Table S7 lists the L10 and S10 transporters of four kidney and two colon CCLs. Five L10 transporters (A43, A24, A14, A19, A1) and five S10 transporters (A48, A25, A4, A29, A33) together identify CCLs as originating from kidney (Fig. 2C,G). Similarly, L10 transporters (A22, A32, A19, A39, A15) and S10 transporters (A34, A24, A20, A41, A18) identify CCLs as originating from colon (Fig. 2D,H). The CCL L10 and S10 sets may differ somewhat from those of the original cancers due to post purification mutations^21^.

L10 transporters A22 and A32 are present in the six CCLs (Supplementary Table S7) but L10 A43 only in the kidney CCLs. We used the kidney Caki-1 CCL, which originated from ccRCC, to generated CCLs with doxycycline-inducible knockdown (kd) of A22, A32, and A43 transcripts to establish the transporters’ importance to CCL proliferation and expression of related enzymes.

A22 has been shown to transport glutamate^22^. Search for a glutamine transporter has been extensive^23^. Knockdown of A22 potently inhibits Caki-1 cell proliferation (Supplementary Fig. S3A). This inhibition is not due to A22’s role as glutamine transporter since Caki-1 cells grown in the absence of glutamine or in the presence of glutamine but with A22 knocked down do not yield similar expression profiles of metabolic enzymes (Supplementary Fig. S3B). A mitochondrial glutamine transporter has recently been identified^24^ (Fig. 1A). The reason A22 is among L10 of CCLs but not of ccRCC (Fig. 2C) is not due to the high glutamine concentrations in the tissue culture medium since there was no significant difference between transcript concentration of A22 in cells grown in medium with 4 mM or 0.1 mM glutamine (Supplementary Fig. S3C).

A32 is an L10 of all kidney CCLs (Supplementary Table S7) but not of ccRCC (Fig. 2C–F). A32 replenishes the mitochondrial matrix tetrahydrofolate (THF) as cells and mitochondria proliferate. Accordingly, A32-kd Caki-1 CCL cells proliferate slower (Supplementary Fig. S4A) and show a significantly altered expression of the cytosolic KGA and both the cytosolic and the mitochondrial serine hydroxymethyltransferase enzyme genes (Supplementary Fig. S4B). These results are consistent with an important metabolic role for A32.

A43 is an L10 only of the kidney CCLs and ccRCC (Fig. 2C–F; Supplementary Table S7). Its knockdown reduces the Caki-1 proliferation rate (Supplementary Fig. S5A). Such Caki-1 cells show significant expression changes of KGA and A22 (Supplementary Fig. S5B). The reduced proliferation rate reduces the need for C1 metabolism reactions (synthesis of nucleotides, mitochondrial protein synthesis reactions) as reflected by transcript ratios for SHMTs.

### Affinity propagation of L26-S24 gene expression in human tumors and normal tissues

Expression of SLC25 L26-S24 genes set in 6825 TCGA and 2322 GTEx samples were trained by affinity propagation. Based on the raw transporter gene expression data, this analysis yielded 44 clusters. The cluster distributions for twelve of the primary tumor types and normal tissues are shown in Supplementary Fig. S6. Remarkably, many cancer types show very little overlap between clusters harboring tumor and those harboring normal tissue.

**Figure 3:**
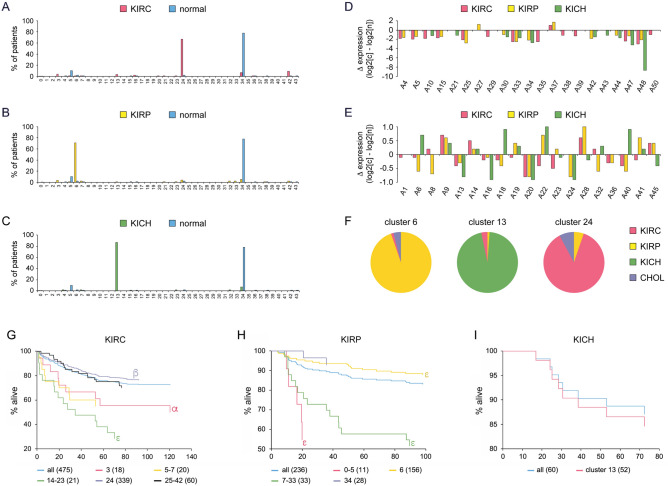
SLC25 gene expression in the three major kidney cancers. KIRC (n = 475), KIRP (n = 236), KICH (60), and normal kidney (n = 138). (**A**–**C**) Distribution of cancer and normal patients in SLC25 expression clusters. (**D**) Expression of L26-S24 genes ≥ twofold or (**E**) less than < twofold larger in KIRC (cluster 24), KIRP (clusters 6), and KICH (cluster 13) cancers (c) compared to normal (n) tissue (cluster 34). Expression is median of the ratio of tumor (Supplementary Fig. S9) and normal (Supplementary Fig. S10) box plot. (**F**) Pie charts for the principal kidney tumor expression clusters. (**G**–**I**) Kaplan–Meier plots. ‘all’ is total population of cancer type. The number of patients is noted in parentheses. Statistical analyses compared survival of patients of a single cluster or defined group of clusters to the total patient cancer type population. α = p < 0.05; β = p < 0.025; γ = p < 0.02; δ = p < 0.01; ε = p < 0.001.

The results for six primary tumor types and normal tissues are described in more detail, as they exemplify the utility of SLC25 expression patterns in human cancer. The three different forms of kidney cancer: KIRC, KIRP, and KICH show a highly distinct distribution of cancers with about 75% of tissues mapping to a single cluster: cluster 24 for KIRC, 6 for KIRP, 14 for KICH, and 34 for normal tissue (Fig. 3A–C). Are these distinct cluster distributions for KIRC, KIRP and KICH due to differences in expression of only one or two SLC25 genes? Figure 3D,E show the ratio of the mean transporter expressions in cancer over that of normal tissue obtained from data in Supplementary Figs. S9 and S10. There are only small differences for the three renal tumor types. The differences become dominant after affinity propagation when the expression of all of the L26-S24 transporters is considered collectively. The pie plots of Fig. 3F demonstrate how unique the three main clusters of the three renal cancer types are. Only a few patients with the closely similar transporter expression profiles yet different cancer types belong to these clusters. Equally striking are the Kaplan–Meier plots (Fig. 3G–I). While a combination of all patient data for each cancer type shows a typical survival curve, patients from several clusters exhibit significantly different survival. Taken together, the results show that, in aggregate, SLC25 expression patterns are highly distinct between KIRC, KIRP and KICH and moreover can identify patient populations with higher or lower survival relative to the total cancer type patient population.

**Figure 4:**
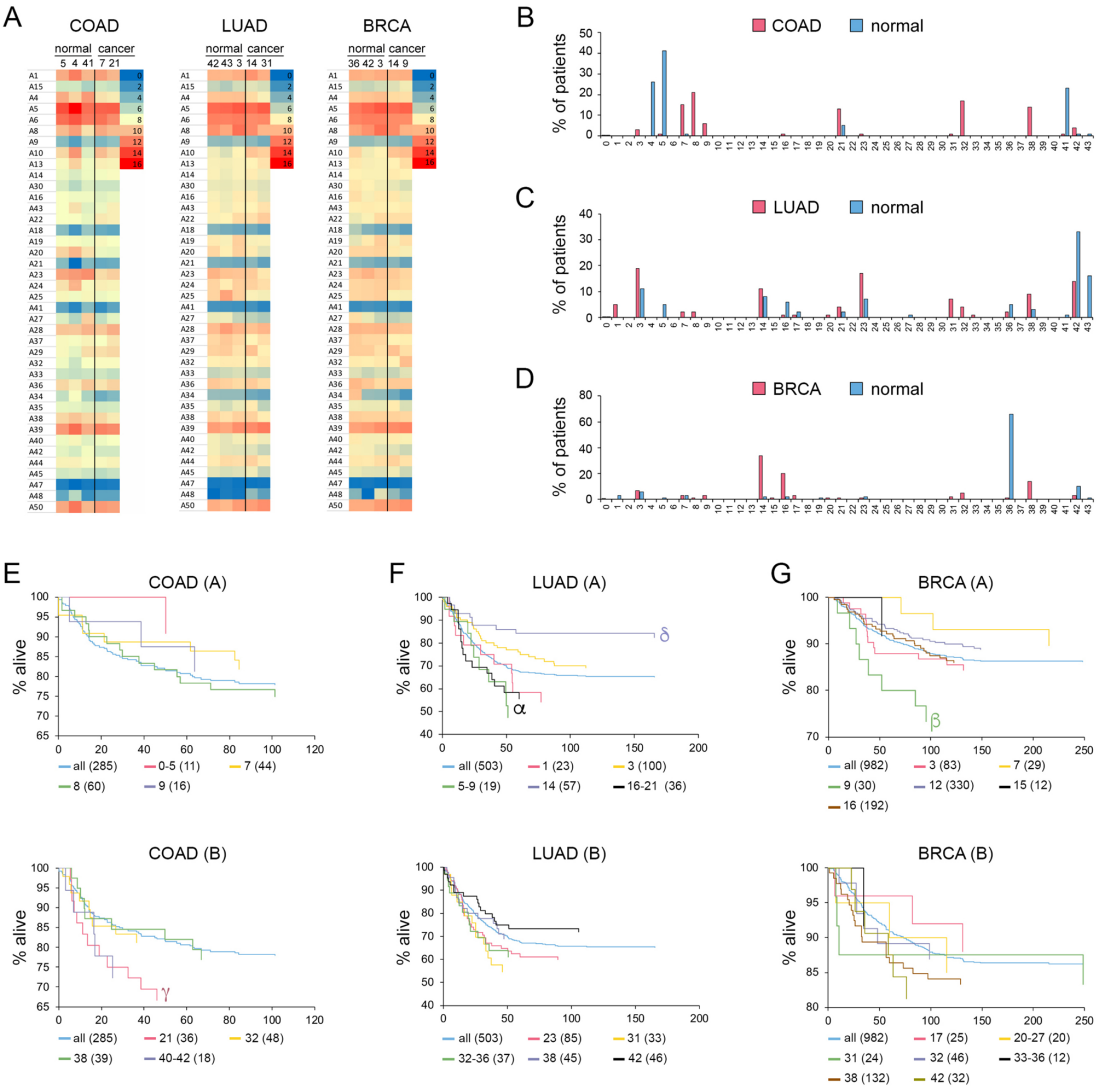
SLC25 expression of cancer types and matched normal tissue. COAD (n = 285); LUAD (n = 503); BRCA (n = 982). (**A**) Heat maps of gene expression of the indicated clusters of tumor and normal tissue L26-S24 SLC25 transporters. (**B**–**D**) Distribution of cancer and normal patients among SLC25 expression clusters. (**E**–**G**) Kaplan–Meier plots of patients grouped by SLC25 expression cluster. ‘all’ refers to total number of patients with the cancer type. Number in parenthesis is the number of patients within cluster or group of clusters used. Statistical analysis between survival of patients of SLC25 expression cluster(s) and the total patient population of the cancer type. α = p < 0.05; β = p < 0.025; γ = p < 0.02; δ = p < 0.01; ε = p < 0.001.

Colon cancer project COAD, lung adenocarcinoma project LUAD, and breast cancer project BRCA in TCGA were looked at more closely. Figure 4A shows the expression of the SLC25 transporters for normal tissue within three different clusters of each cancer type, as well as for patients from cancer clusters with much better or much worse prognosis than the typical patient of each of the three cancer types as detailed below. Again, differences for the cancers only become clearly apparent after affinity propagation.

The cluster plots of COAD, LUAD and BRCA are shown in Fig. 4B–D, respectively. While COAD and BRCA conform to the general scheme of excellent separation between cluster distributions for tumor and normal tissue, LUAD represents an exception. This is surprising given that LUSC shows rather minimal overlap between tumor and normal tissue clusters (Supplementary Fig. S6G). This finding suggests that for LUAD patients, the expression profile of the SLC25 transporters is similar for tumor and normal tissue. It remains possible that a better separation might be obtained by refining the affinity propagation protocol.

We explored whether there are clinical differences between patients with the same form of cancer but with SLC25 expression profiles belonging to different clusters as already noted for the kidney cancers. We plotted all COAD, LUAD and BRCA cancers taken through affinity propagation to generate Kaplan–Meier plots (Fig. 4E–G). Survival curves for the total population of patients with these cancer types are indicated by blue lines, while survival curves for patients belonging to cancers of individual clusters or groups of clusters are shown in distinct colors. Clear differences in survival are observed between patients of different clusters. For instance, COAD patients in clusters 7, 8, and 9 have a significantly better prognosis than those in cluster 21 (Fig. 4E). Similarly, Kaplan–Meier plots for LUAD and the BRCA patients also exhibit some dramatic differences in survival between those in different clusters (Fig. 4F,G). This is striking, because it suggests that even though separation of clusters for tumor and normal tissue is rather poor for LUAD, clustering may still be highly useful to predict survival. Kaplan–Meier plots for other cancer types and subtypes are shown in Supplementary Figs. S7 and S8.

### Paired cancer subtypes with different survivals of four cancer types are differentiated by metabolic enzymes

**Figure 5:**
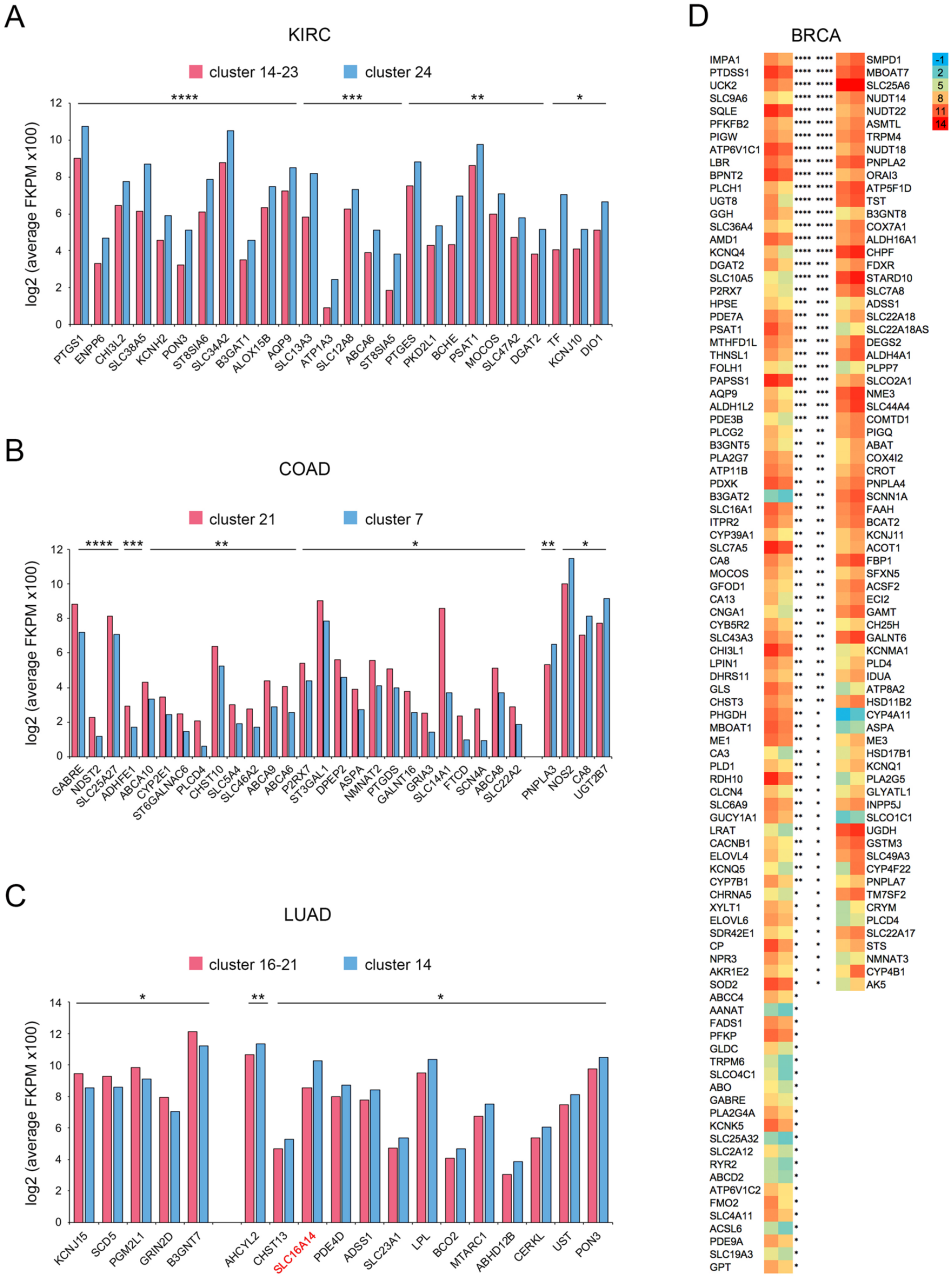
Metabolic enzymes with expressions that differentiate worse from better prognosis cancer subtypes. (**A,B**) Only those enzymes are shown with expression ratio of worse prognosis (red) over better prognosis (blue) of ≥ 2.0 and ≤ 0.5. (**C**) Only SLC16A14 falls into the range of ratios of (**A**,**B**). All others fall into smaller (2.0 ≥ 1.5) and larger (0.7 ≥ 0.5) ratios. (**D**) Same ratio ranges as (**A**,**B**) with left column ≥ 2.0 and right column ≤ 0.5. The left squares of both columns are of cancer subtypes with worse prognoses (clone 9) while those on the right are of cancer subtypes with better prognosis (clone 7). p < 0.05 (*), p < 0.01 (**), p < 0.001 (***), p < 0.0001 (****).

The SLC25 transporters are intermediates of essential metabolic pathways. Since cancers have been placed into different clusters based on SLC25 expression patters it follows that such cancers should be found to differ in metabolic enzyme expressions. Unstranded fpkm TCGA data of 2080 protein-coding metabolic enzyme genes^25^ were searched for two-fold or greater expression differences between cancer subtypes with better or worse prognosis of a cancer type. Figure 5 identifies such enzymes for subtypes from KIRC, COAD, BRCA, and LUAD cancers. It may appear surprising that while the cancer subtypes of BRCA show by far the largest number of such enzymes, LUAD subtypes show only one. This difference may be due to a greater variety of cancer subtypes still present in the BRCA samples compared to the LUAD subtypes. It should be noted however that more enzymes are present in all the cancer subtypes with expression ratios of less than 2.0 and that are statistically significant.

### SLC25 gene expressions and cancer of unknown primary (CUP)

Almost all cancer therapies rely on the tissue of origin of a cancer. A major obstacle in treating CUP is that an originating tissue is not known. We queried if the clusters defined by affinity propagation could be exploited for tissue of origin identification. We plotted cancer clusters against normal tissue clusters (Table 2). Most cancer types and their subtypes can be separated. However, there are distinct cancer types with the same location in this plot. These are of diagnostic and therapeutic interest since their metabolic pathways are similar. Might they respond to similar therapeutic intervention? By definition, we do not have access to data of the normal tissue for CUPs. However, once the amount of normal tissue data in GTEx is expanded, CUP’s tissue-of-origin will be identifiable with an even higher degree of confidence.

**Table 2:**
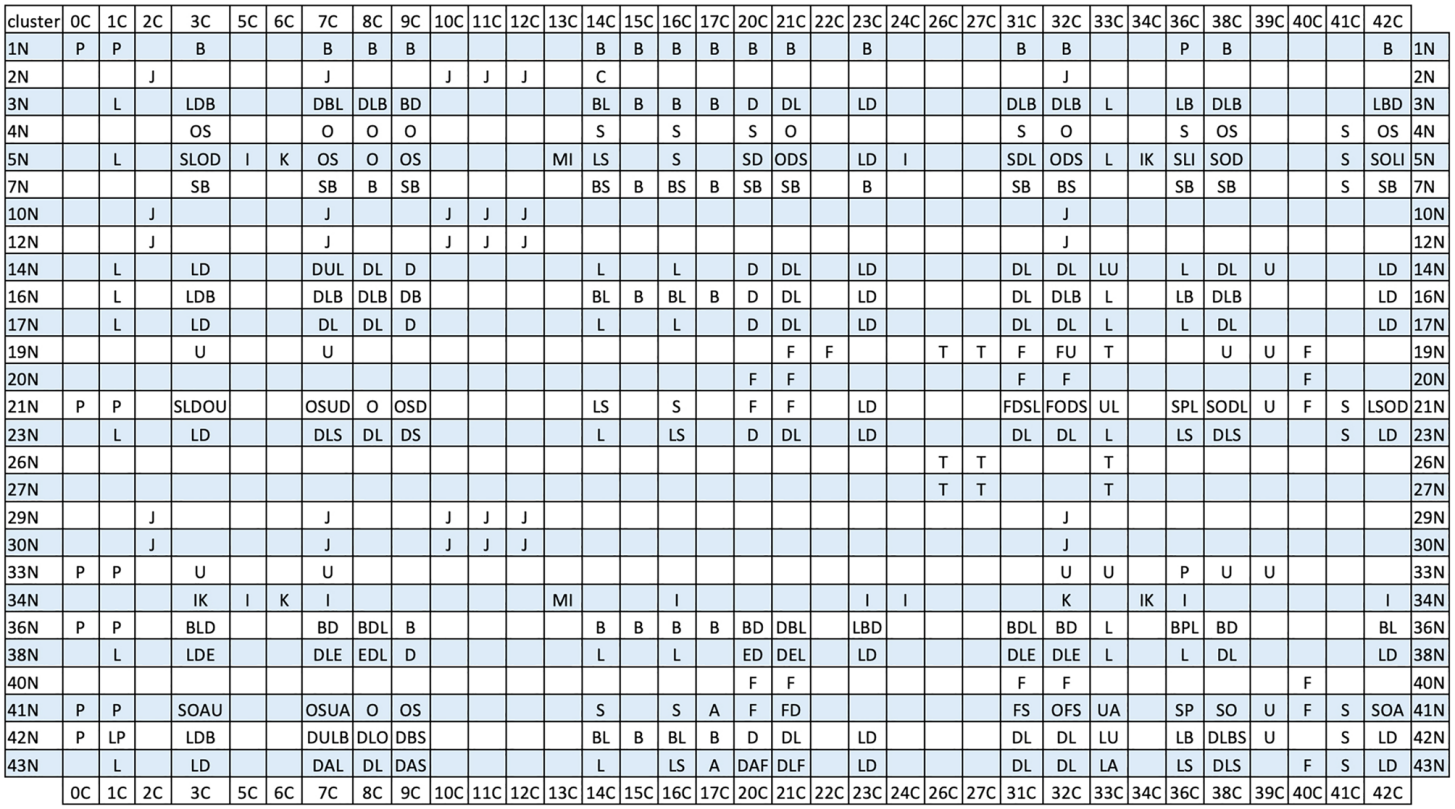
Tissue of origin of cancers.

Cancer clusters (horizontal) vs. normal clusters (vertical). A cancer type is listed if the product of its number of cancer tissue samples times its number of normal tissue samples for a cluster is ≥ 10% of that of the cancer type with the largest product within the table’s cell. Tissue clusters with ≥ 5 samples were used in the analysis. Cancer types at each location are listed with decreasing values from left to right. TCGA cancers: A (BLCA, bladder urothelial carcinoma), B (BRCA, breast invasive carcinoma), C (CHOL, cholangiocarcinoma), D (LUSC, lung squamous cell carcinoma), E (CESC, cervical squamous cell carcinoma and endocervical adenocarcinoma), F (ESCA, esophageal carcinoma), I (KIRC, kidney renal clear cell carcinoma), J (LIHC, liver hepatocellular carcinoma), K (KIRP, kidney renal papillary cell carcinoma), L (LUAD, lung adenocarcinoma), M (KICH, kidney chromophobe), O (COAD, colon adenocarcinoma), P (PRAD, prostate adenocarcinoma), S (STAD, stomach adenocarcinoma), T (THCA, thyroid carcinoma), U (UCEC, uterine corpus endometrial carcinoma). H (HNSC, head and neck squamous cell carcinoma), R (READ, rectum adenocarcinoma), and UCS (uterine carcinosarcoma) have too few tissue samples.

## Discussion

Mitochondria are receiving increased attention in the context of cancer. SLC25 transporters link the many metabolic pathways that are split between the mitochondrial matrix and the cytosol. Cellular metabolic patterns are tissue dependent. The L10 and S10 transporters with their FPKM data should be suitable for identifying human cancer types on the basis of their metabolisms.

**Figure 6:**
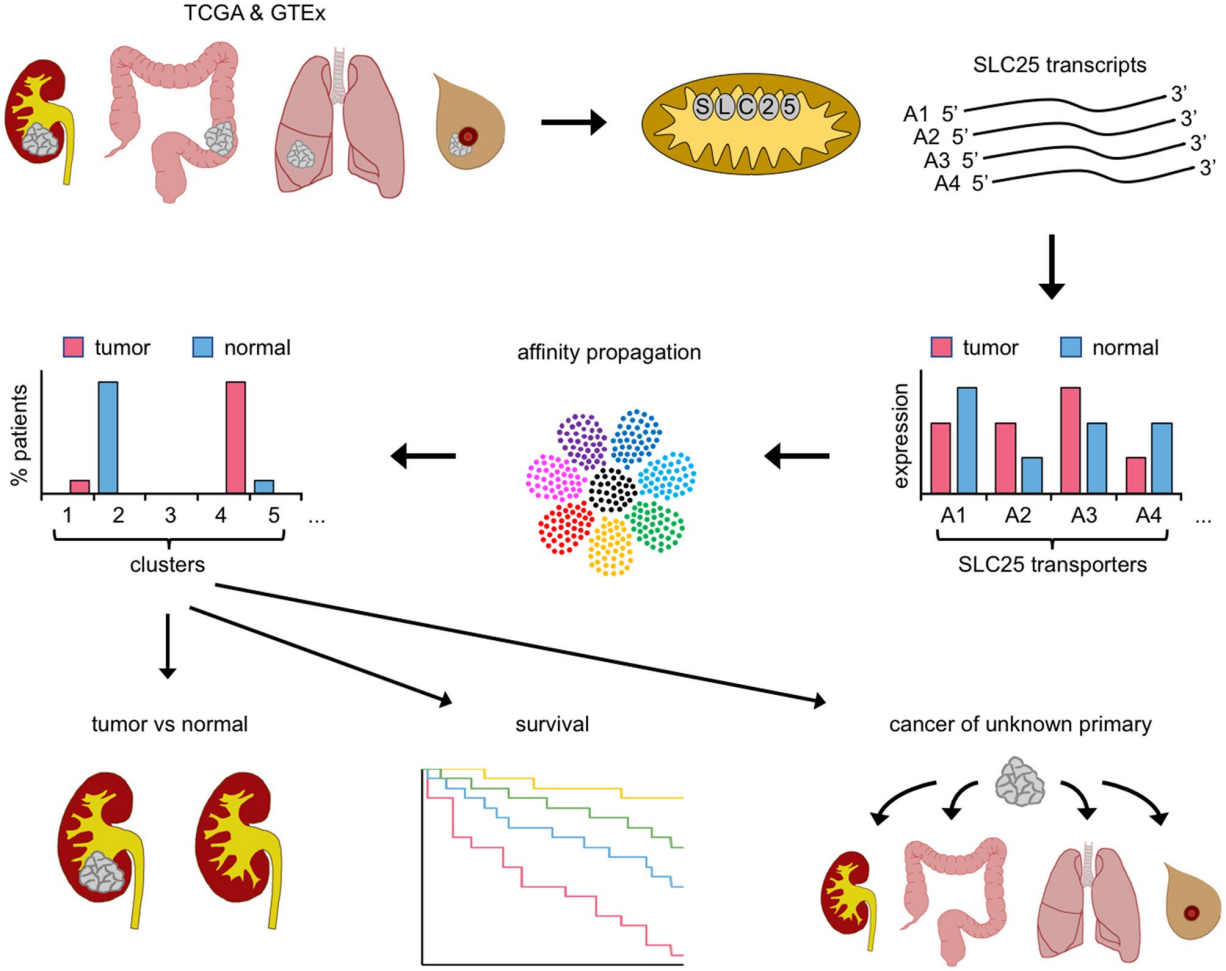
Diagrammatic presentation of the mitochondrial SLC25 transporter algorithm used to identify and characterize human cancer types and subtypes.

The capability of our approach (Fig. 6) was first established by probing four human cancer types: KIRC, COAD, LUAD, and BRCA. We identified for each cancer type of a patient, the ten transporter genes with the largest (L10), as well as the smallest (S10) ratio of cancer over uninvolved tissue expression. Such transporters in the largest number of patients in each of the cancer types were pooled and yielded sets of 26 L10 (L26) and 24 S10 (S24) transporters. They are identifiers for the four cancer types. The importance of L26 transporters to cancer metabolism was demonstrated with kidney Caki-1 CCL cells and L26 genes A22, A32, and A43. Each promotes cell proliferation and modulates cytosolic and mitochondrial C1-metabolism.

Does the L26-S24 transporter set provide unique signatures for other cancer types? We performed affinity propagation with FPKM data of 19 human cancer types and normal tissues. Most clusters showed a high degree of tissue specificity. This is exemplified by KIRC, KIRP, KICH and the normal tissue. High tissue specificity is also observed for LIHC, PRAD, and THCA. For some tissues there is overlap with other cancer types, i.e., LUAD, LUSC and COAD. LUSC and CESC exhibit considerable overlap, as did the normal tissues. BRCA map into distinct clusters. This must reflect the numerous breast cancer subtypes as also suggested by the expression of a large number of metabolic enzymes that differentiate BRCA subtypes.

The SLC25 (L26-S24 transporter set) expression-based clusters often distinguish between tumor and normal tissue. CHOL indicates no overlap between clusters populated with normal and tumor samples. BLCA, BRCA, CESC, COAD, HNSC, KIRC, KIRP, KICH, READ, and STAD also show excellent separation of cancer and normal clusters. Such separation of cancer from normal tissue can be of value following endoscopic retrograde cholangiopancreatography or in situations of limited sample size as with liquid biopsies. Refinement of our affinity propagation analysis may prove useful to LUAD where distinction between tumor and normal tissue is limited.

Our study presents a future opportunity, facilitated with the set of L26-S24 transporters, to explore cancer metabolism. The role of single SLC25 transporters, all, except A11, belonging to the L26-S24 transporter set, in CCLs has been explored, i.e., A38^26^, A15^27^, A10^28^, A11^29^, A18^30^, A22^31^, A28^32^, A37^32^, A32^33^, A20^34^, A29^35^. Each study addresses the role of only a single transporter and only in cancer cell lines. In contrast, our present study provides a comprehensive, tissue-based analysis with a set of 41 SLC25 transporters with significant alterations in human cancer types.

The expression of a large percentage of all SLC25 transporter genes is affected by oncogenic transformation (p < 0.0001: 47% KIRC, 23% COAD, 34% LUAD, 49% BRCA) and generally to a different extent in the tissues of different cancer types. To what extent these expression changes are interdependent, transforming, enabling, or neutral^36^ to the oncogenic transformation remains to be established. The L26-S24 gene sets were used to establish clusters. This led to the observation that (a) different cancer types may be located in the same cluster and (b) same cancer types in different clusters often have different survivals. Different cancer types in the same cluster have similar metabolism and may respond to the same therapy. On the other hand, the observation that same cancer type in different clusters has different survival, i.e., a better or a worse prognosis, suggests that such cancer subtypes should possess metabolic enzyme expressions that can be linked to survival differences. We have indeed identified such enzymes. Future research should help identify therapies that take advantage of metabolic differences responsible for differences in survival.

The L26-S24 transporter set points to other important and new practical aspects. A primary reason for an overall poor prognosis for CUP patients is the inability to identify tissue of origin. Mitochondria from different tissues are known to differ dramatically. A most extreme example could be the difference between heart and liver mitochondria^37,38^. We demonstrate here that the set of L26-S24 transporters can identify many cancers and their corresponding normal tissues. The data we have presented suggest that indeed SLC25 expression can be utilized to aid in identifying tissue of origin for CUP. The L26-S24 transporter set may also be of use in the identification of precancerous lesions and metabolic grading of tumors.

## Supplementary Information

Supplementary Information 1.

Supplementary Table S8.

## Data Availability

For cancer (TCGA) and normal (GTEx) data see Supplementary Information Table S8. Additional information is available from the corresponding author on reasonable request.
