# Supplementary material for: Mitochondrial transporter expression patterns distinguish tumor from normal tissue and identify cancer subtypes with different survival and metabolism
Source: Sci Rep. 2022 Oct 11;12:17035. doi: 10.1038/s41598-022-21411-0 (PMC9553943; doi:10.1038/s41598-022-21411-0)
Supplement: Supplementary file 1 — Supplementary Information 1. [file 41598_2022_21411_MOESM1_ESM.pdf]

Supplementary information

Mitochondrial Transporter Expression Patterns Distinguish Tumor from Normal Tissue and Identify Cancer Subtypes with Different Survival and Metabolism

Hartmut Wohlrab,[1,2,7*] Sabina Signoretti,[3,4] Lucia E. Rameh,[5] Derrick K. DeConti,[6*] Steen H. Hansen[2,4]

[1]Department of Biological Chemistry and Molecular Pharmacology, Harvard Medical School, 240 Longwood Avenue, Boston MA 02115.
[2]GI Cell Biology Research Laboratory, Boston Children's Hospital, 300 Longwood Ave, Boston, MA 02115.
[3]Department of Pathology, Brigham and Women's Hospital, 75 Francis Street, Boston, MA 02115.
[4]Harvard Medical School.
[5]Department of Biochemistry, School of Medicine, Vanderbilt University, 2209 Garland Ave, Nashville, TN 37240.
[6]Quantitative Biomedical Research Center, Department of Biostatistics, Harvard T.H. Chan School of Public Health, 655 Huntington Ave, Boston, MA 02115.
[7]Lead contact
[*]Correspondence:
hartmut_wohlrab@hms.harvard.edu (H.W.), tel: 6178332497
ddeconti@hsph.harvard.edu (D.K.D.)

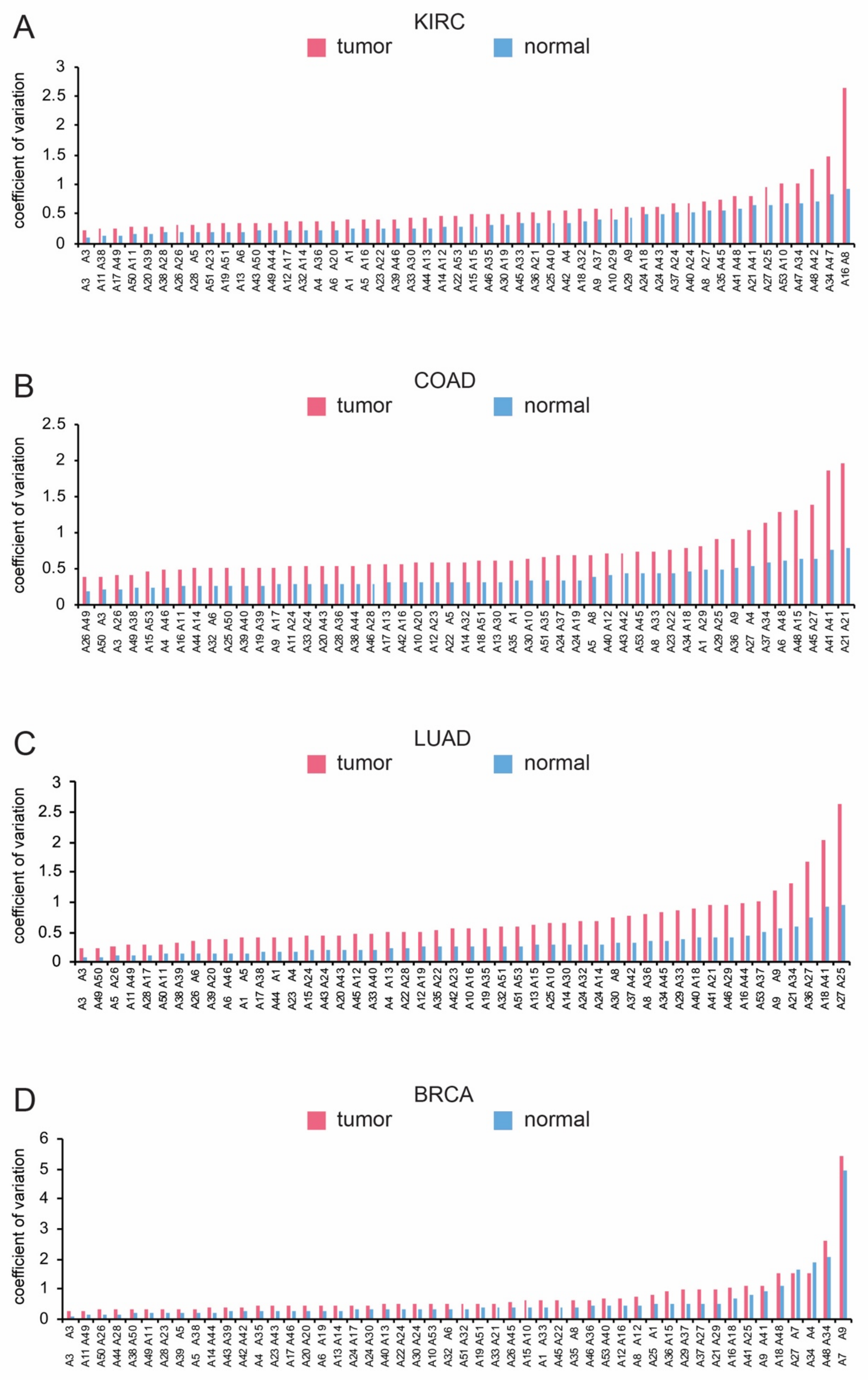

Figure S1.

Coefficient of variation (standard deviation/mean) of transporter FPKM/sum of MitoCarta gene FPKMs from tumor and normal tissue for kidney [(**A**) KIRC)], colon [(**B**) COAD)], lung [(**C**) LUAD], and breast [(**D**) BRCA].

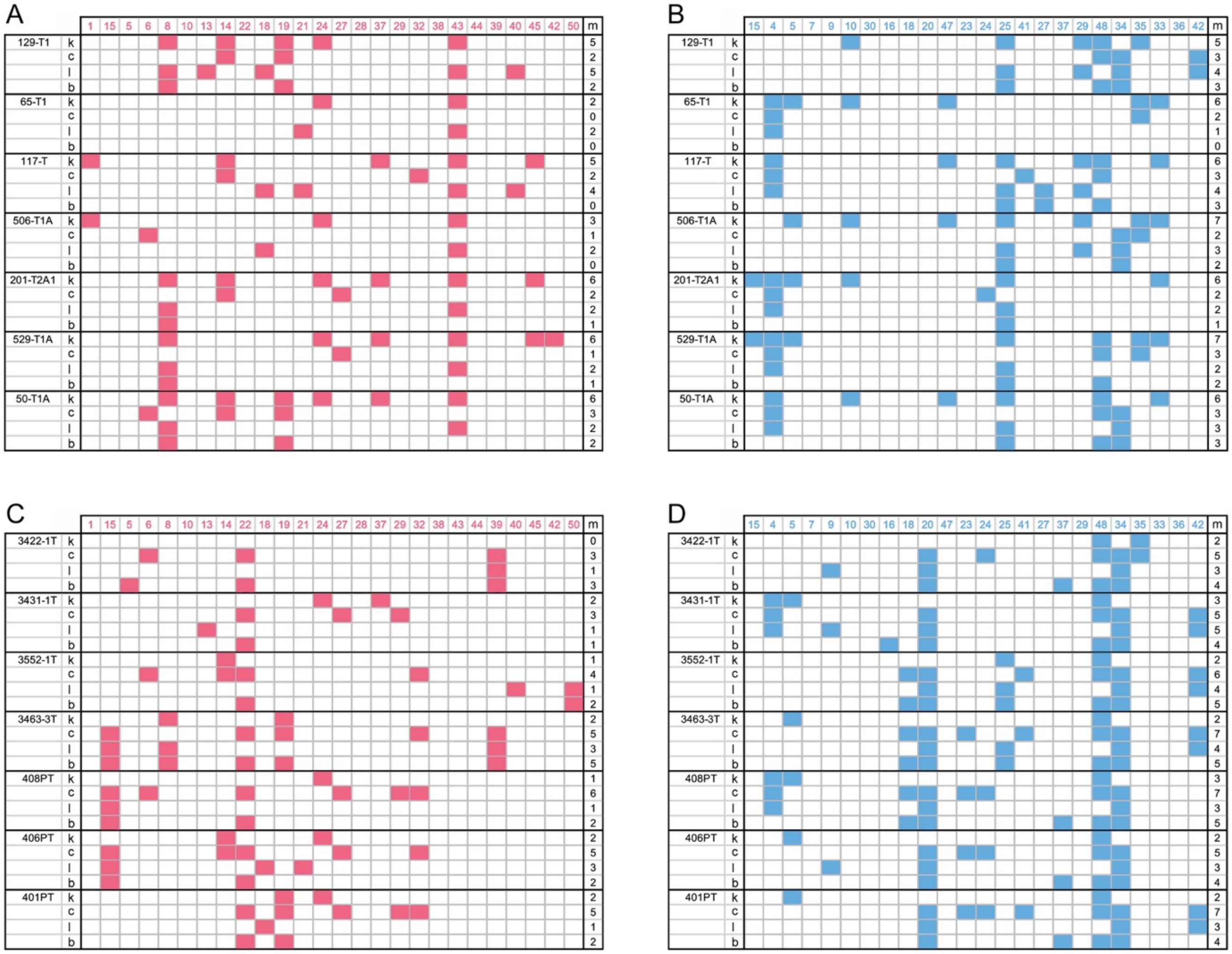

Figure S2.

Identification of tissue of origin of clear cell renal cell carcinomas (KIRC) and colon adenocarcinoma (COAD) by transporter sets. RT-qPCR-obtained transcripts were used to obtain tumor over normal tissue transcript ratios. Horizontal axis lists the SLC25 genes by number only, i.e., A15 becomes 15. Along the vertical axis ‘k’, ‘c’, ‘l’, and ‘b’ denotes kidney, colon, lung, and breast, respectively. Identification codes of KIRC tumors: 129-T1, 65-T1, 117-T, 506-T1A, 201-T2A1, 529-T1A and 50-T1A. Identification codes of COAD tumors: 3422-1T, 3431-1T, 3552-1T, 3463-3T, 408T, 406PT and 401PT. ‘M’ indicates the number of L10 and S10 transporters of each of the 14 tumors that match the L26 and S24 set of Figure 2. **A**, Matches for L10 transporters in KIRC. **B**, Matches for S10 transporters in KIRC. **C**, Matches for L10 transporters in COAD. **D**, Matches for S10 transporters in COAD.

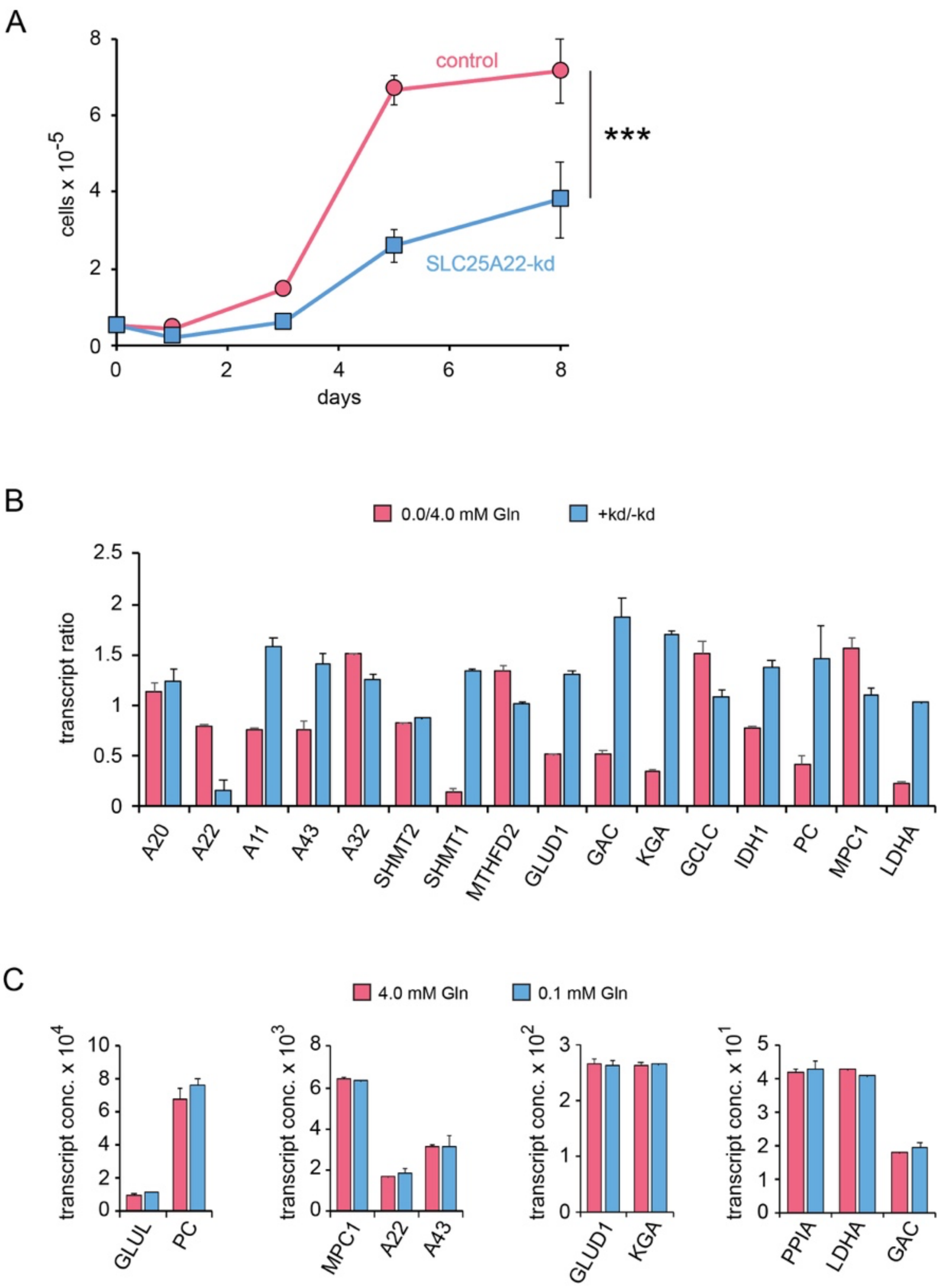

Figure S3.

Effect of SLC25A22 depletion and glutamine on Caki-1 cells. **A**, Growth curves of Caki-1 cells with or without knockdown of SLC25A22. Error bars represent standard deviations (n=3); δ = p<0.01. **B**, Effect of glutamine deprivation (red bars) or SLC25A22 depletion on transcript ratios of genes as listed on the abscissa. Error bars represent standard deviations (n=4). **C**, Transcript concentrations of indicated genes of Caki-1 cells grown in DMEM medium (10% fetal bovine serum supplemented) with 4.0mM or 0.1mM glutamine. Error bars show range (high-low/2) of duplicate experiments.

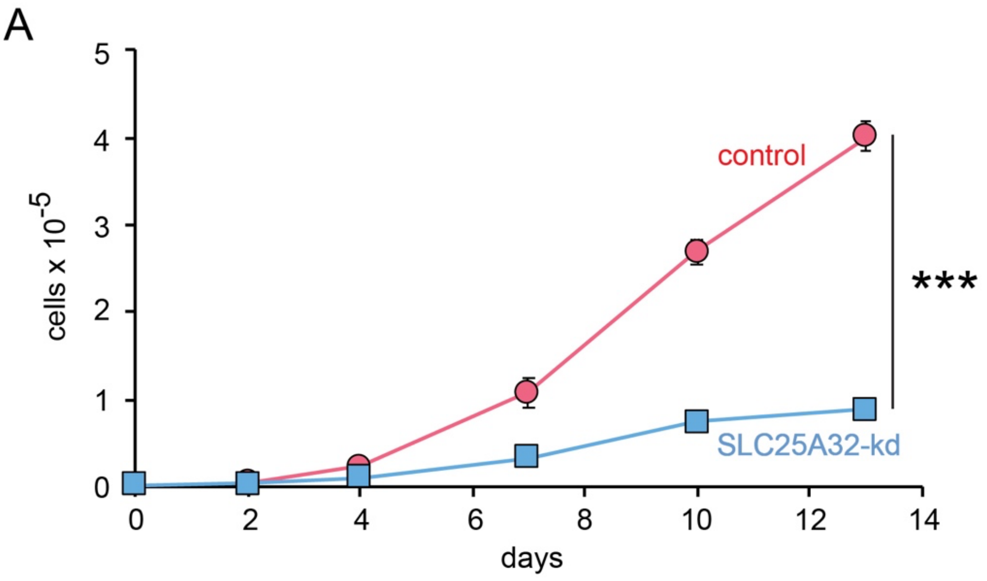

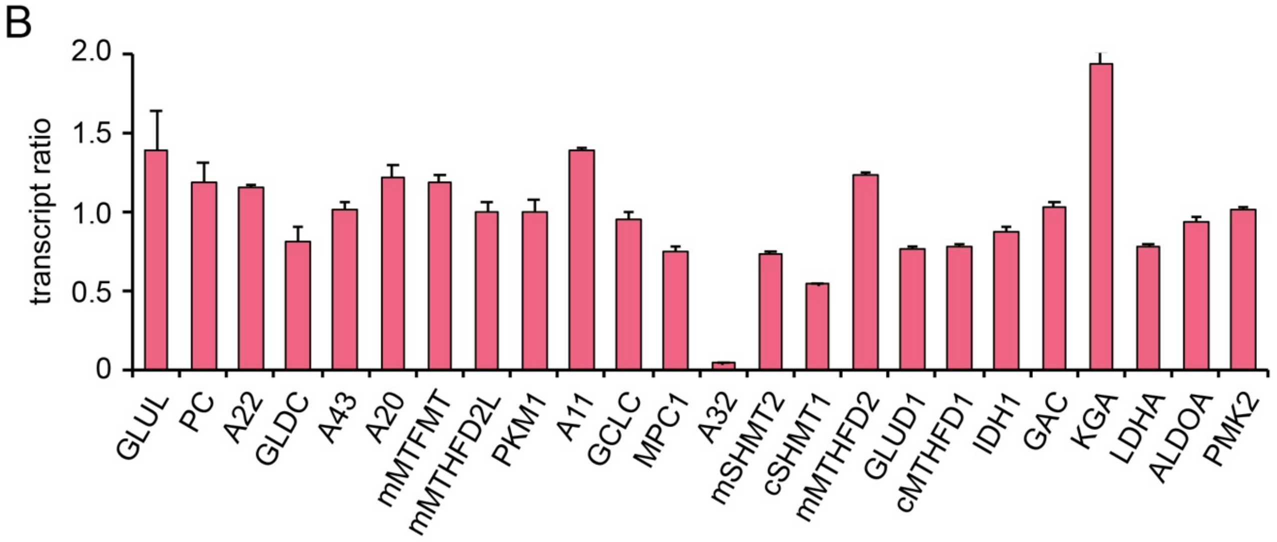

Figure S4.

Effect of SLC25A32 depletion on Caki-1 cells. **A**, Growth curves of Caki-1 cells with and without knockdown of SLC25A32. Error bars represent standard deviations (n=3). **B**, Ratios of gene transcripts of SLC25A32 knockdown cells over control cells. Error bars represent standard deviations (n=4). Labels ‘m’ and ‘c’ of enzyme abbreviations refer to mitochondrial and cytosolic, respectively.

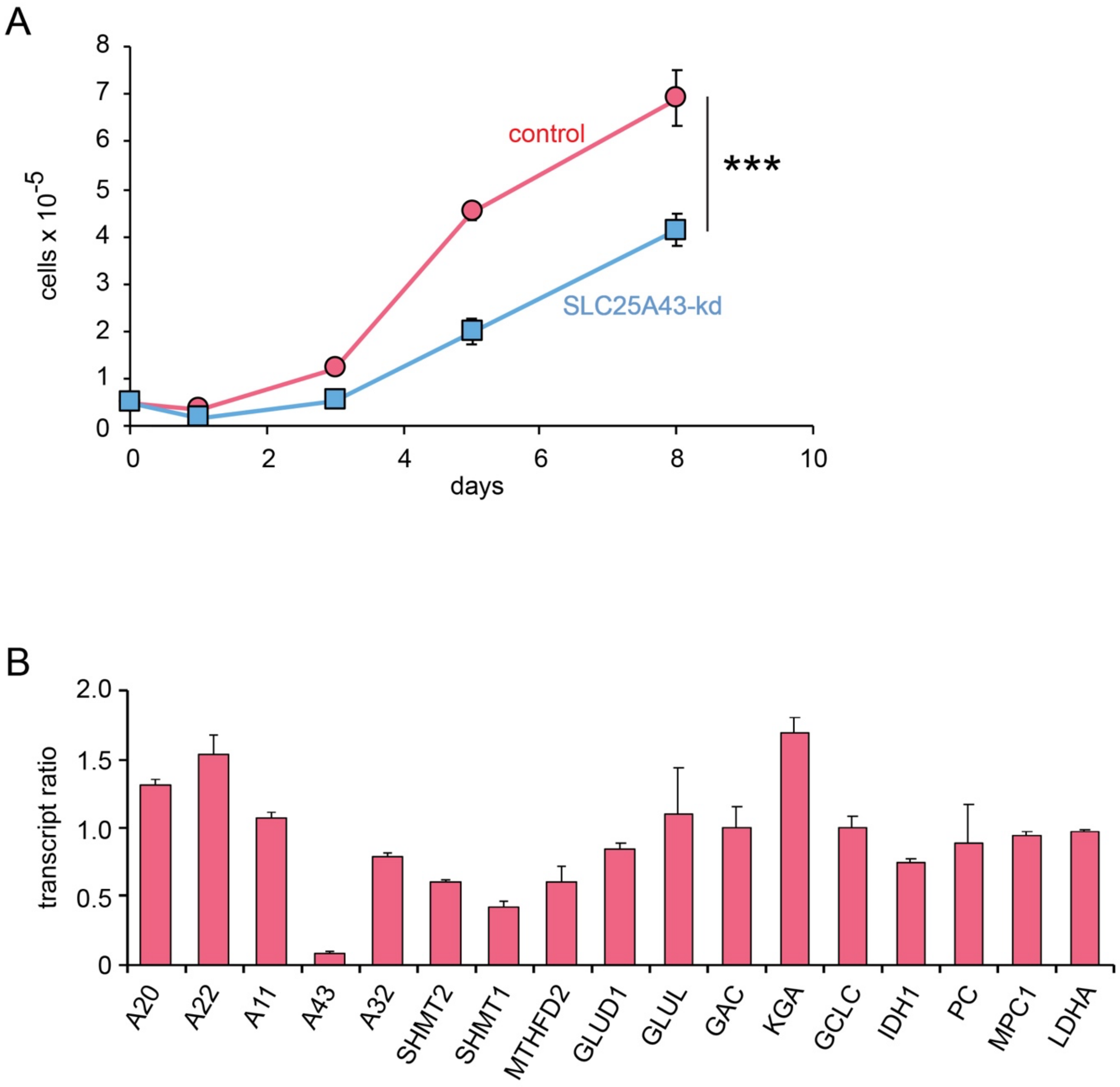

Figure S5.

Effect of SLC25A43 depletion on Caki-1 cells. **A**, Growth curves of Caki-1 cells with or without A43 knockdown. Error bars represent standard deviations (n=3). **B**, Ratios of gene transcripts of SLC25A43 knockdown cells over control cells. Error bars represent standard deviations (n=4).

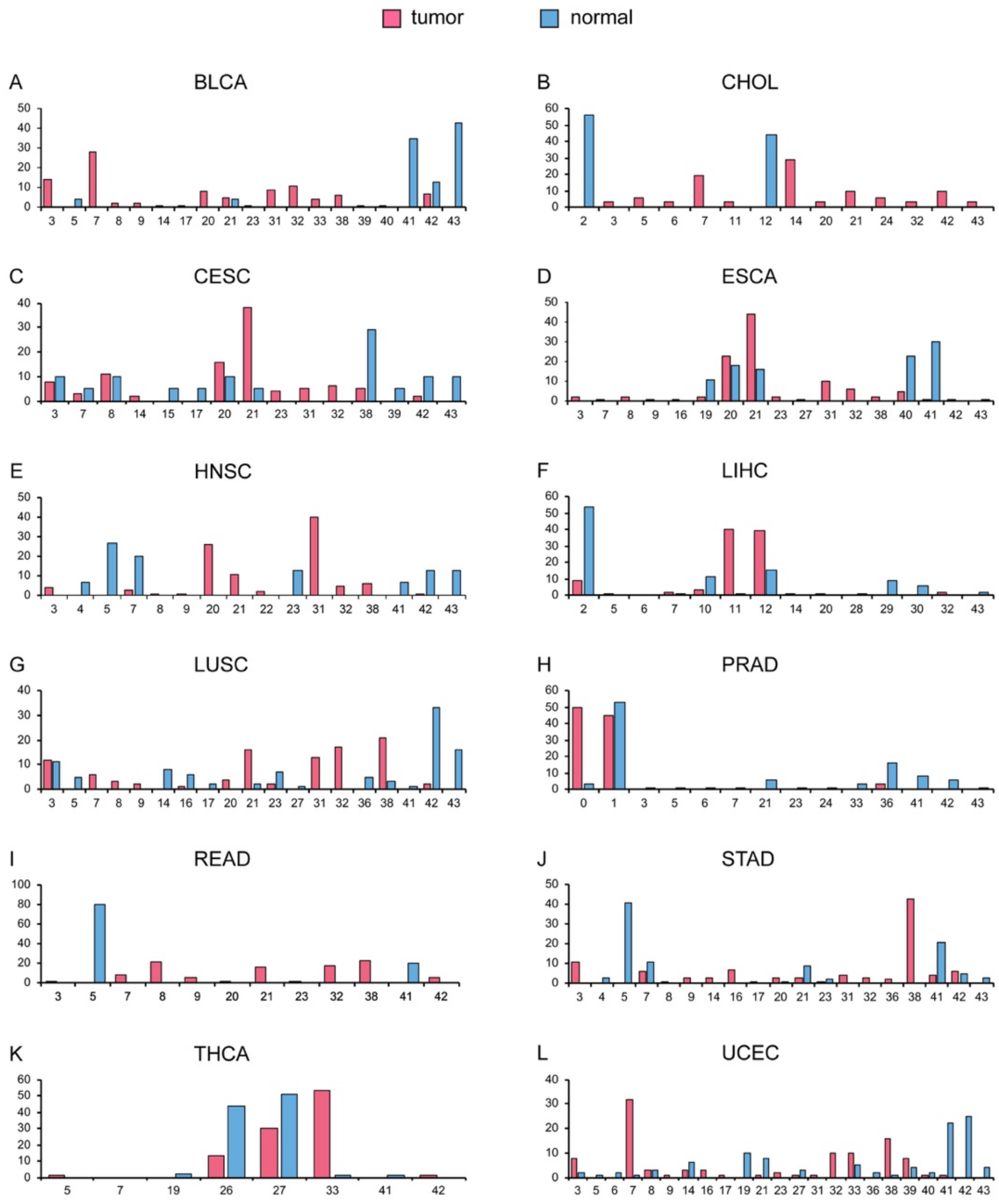

Figure S6.
SLC25 transporter expression cluster distributions for twelve forms of human cancer and corresponding normal tissues.

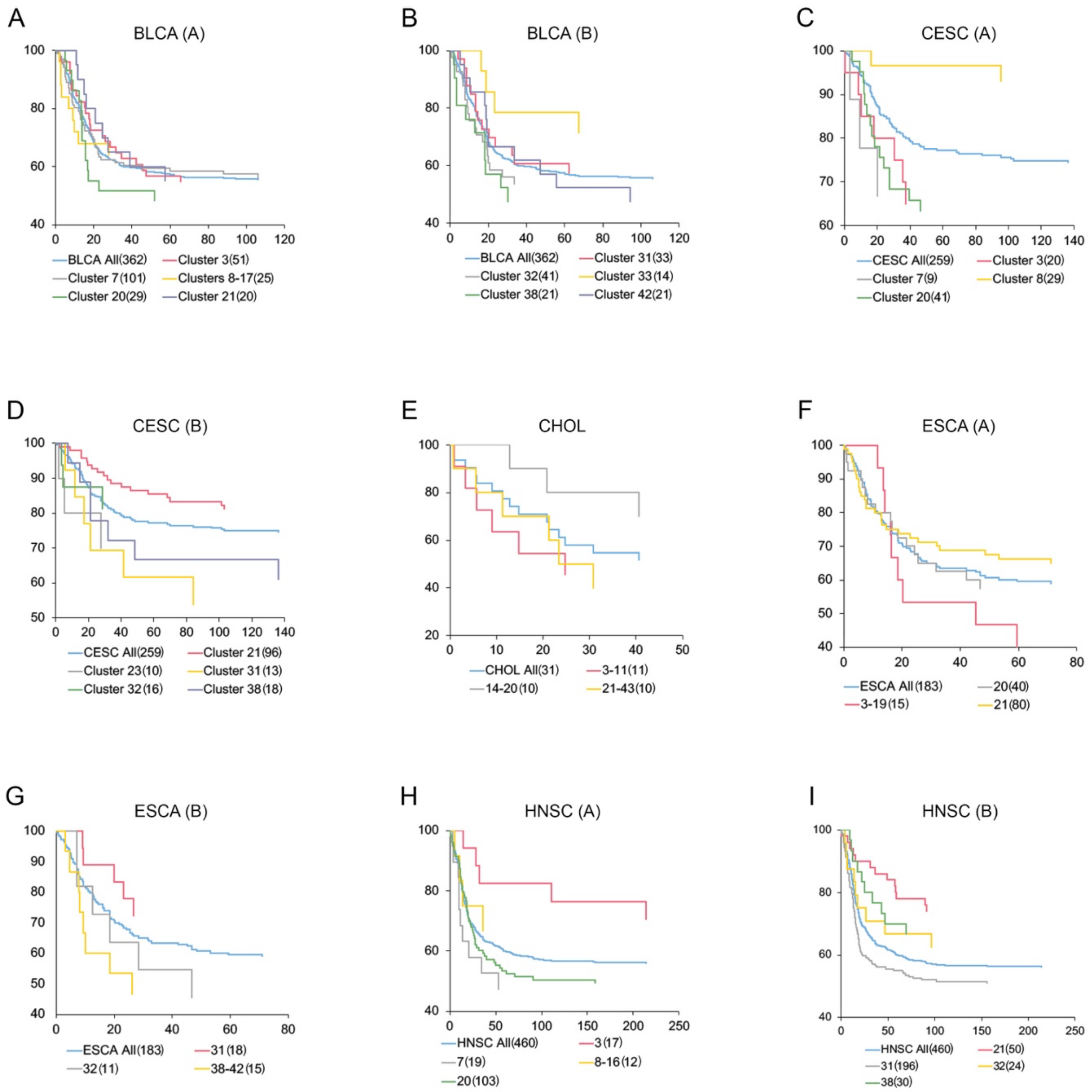

Figure S7.

Kaplan-Meier plots of patients grouped by SLC25 expression cluster, as well as the total number of patients with a given cancer form (‘All’). The number of patients of each cluster or group of clusters used in the Kaplan-Meier plots is noted in parentheses.

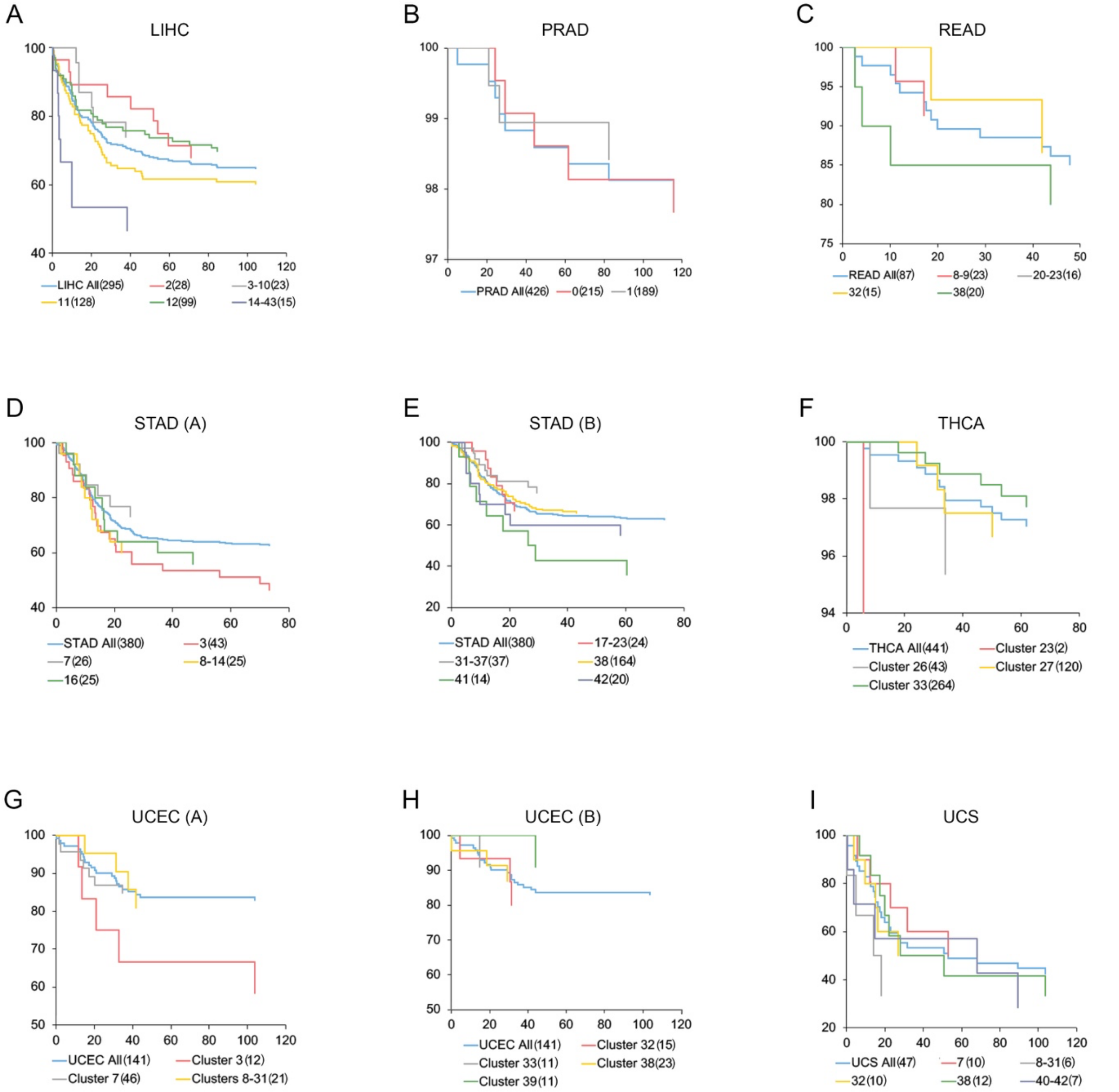

Figure S8.

Kaplan-Meier plots of patients grouped by SLC25 expression cluster, as well as the total number of patients with a given cancer form (‘All’). The number of patients of each cluster or group of clusters used in the Kaplan-Meier plots is noted in parentheses.

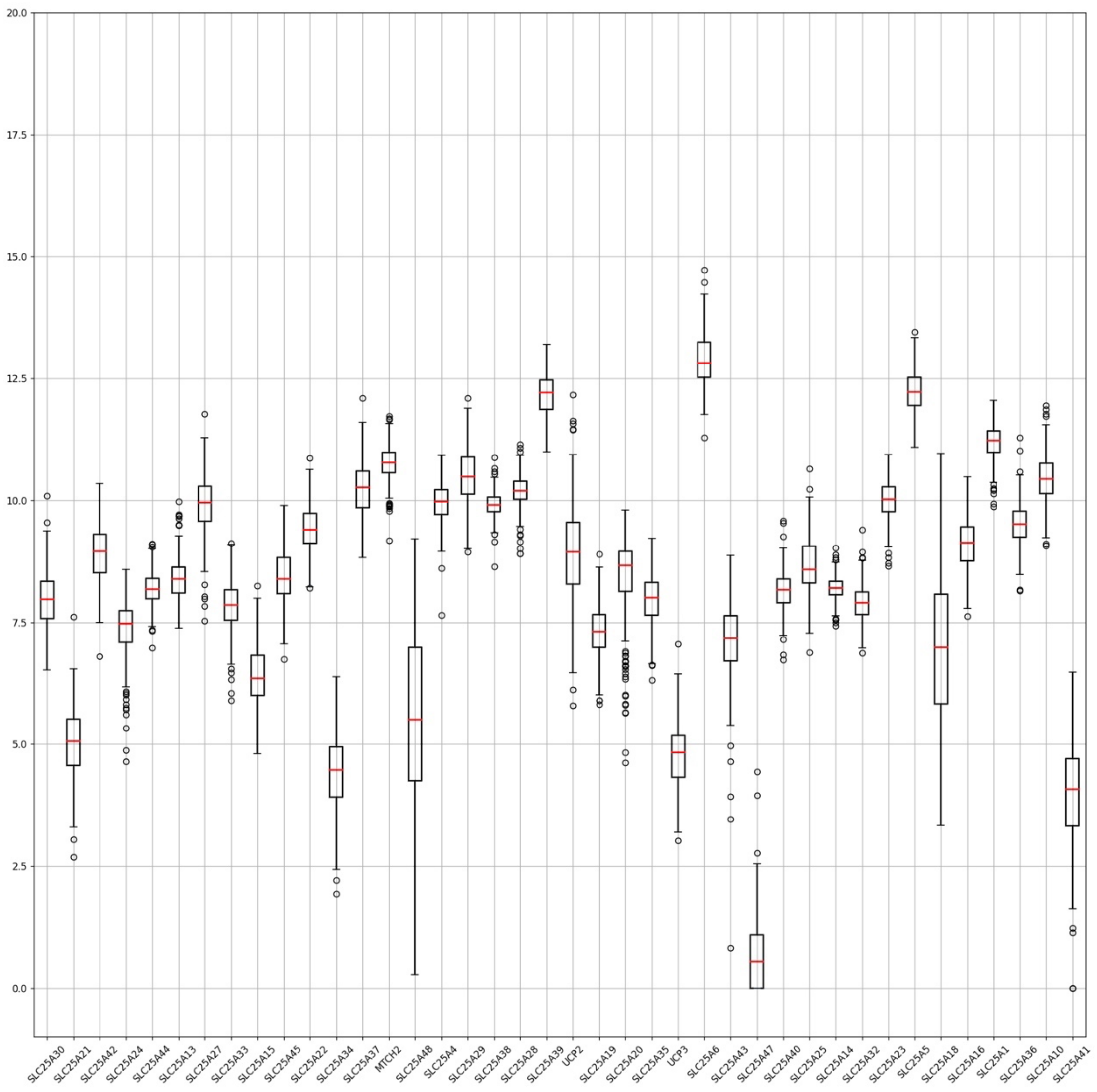

Figure S9A.

Boxplot of SLC25 transporter expression cluster 0 of TCGA tumors (log_2FPKM)

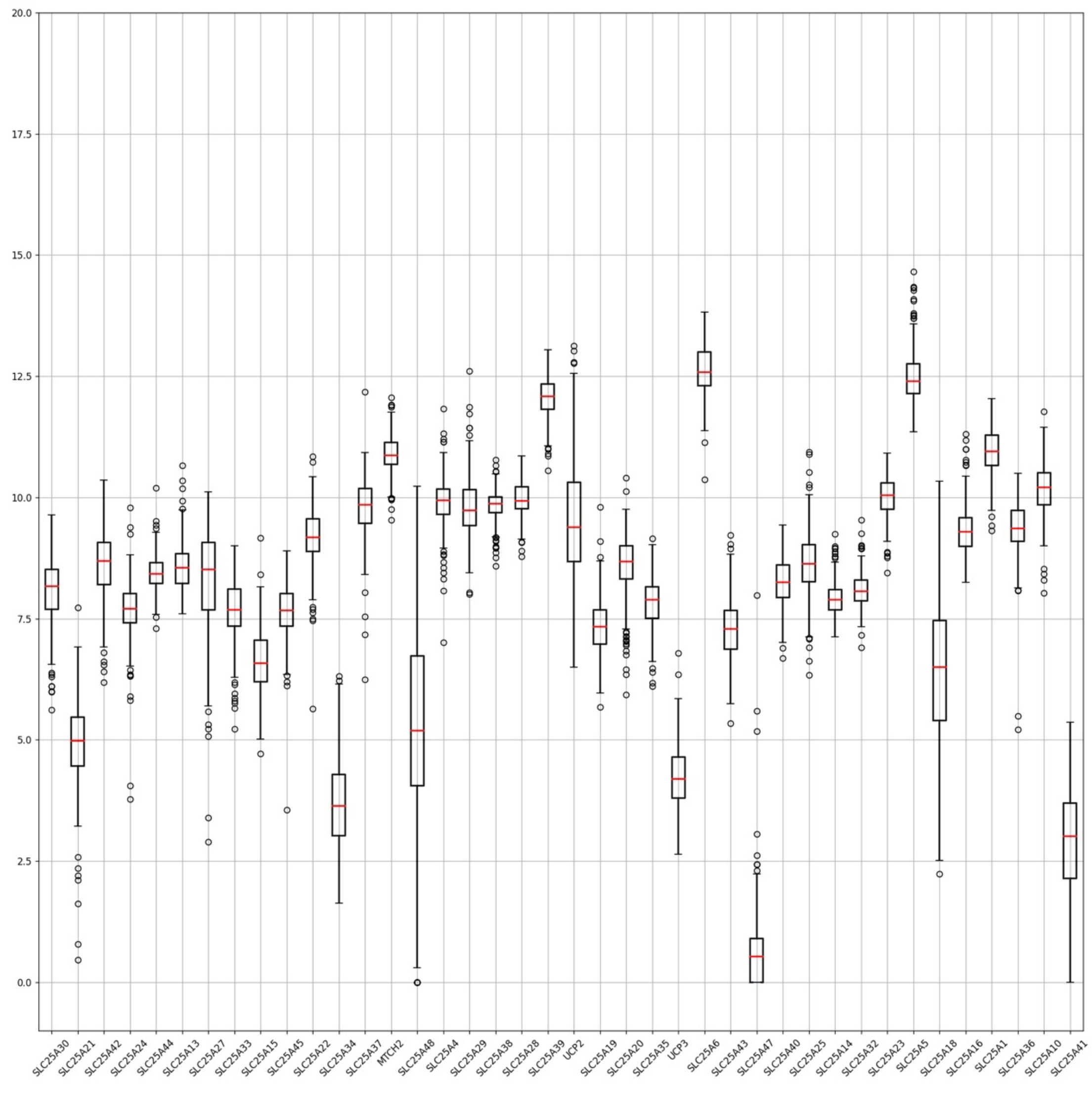

Figure S9B.

Boxplot of SLC25 transporter expression cluster 1 of TCGA tumors (log_2FPKM)

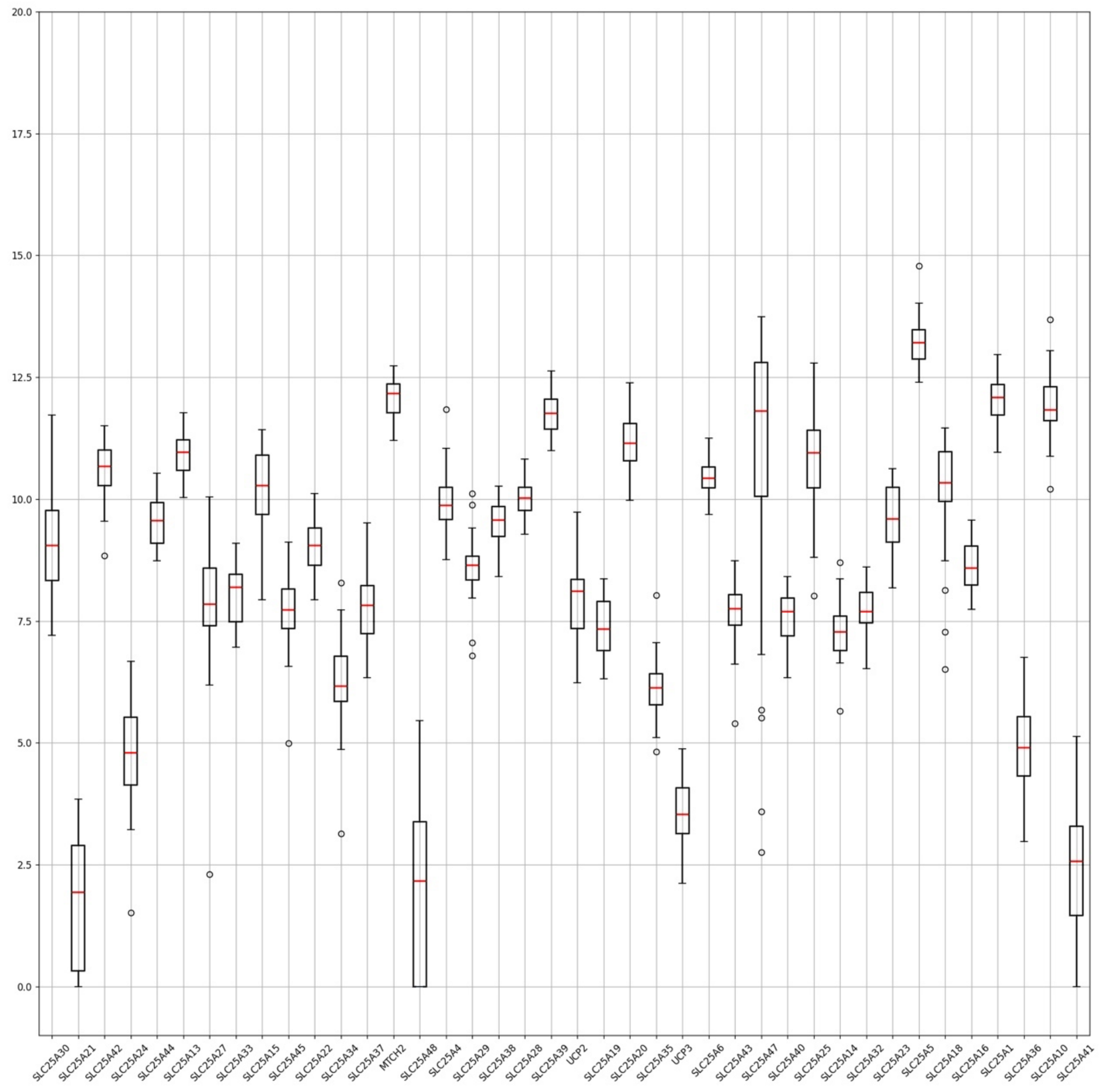

Figure S9C.

Boxplot of SLC25 transporter expression cluster 2 of TCGA tumors (log_2FPKM)

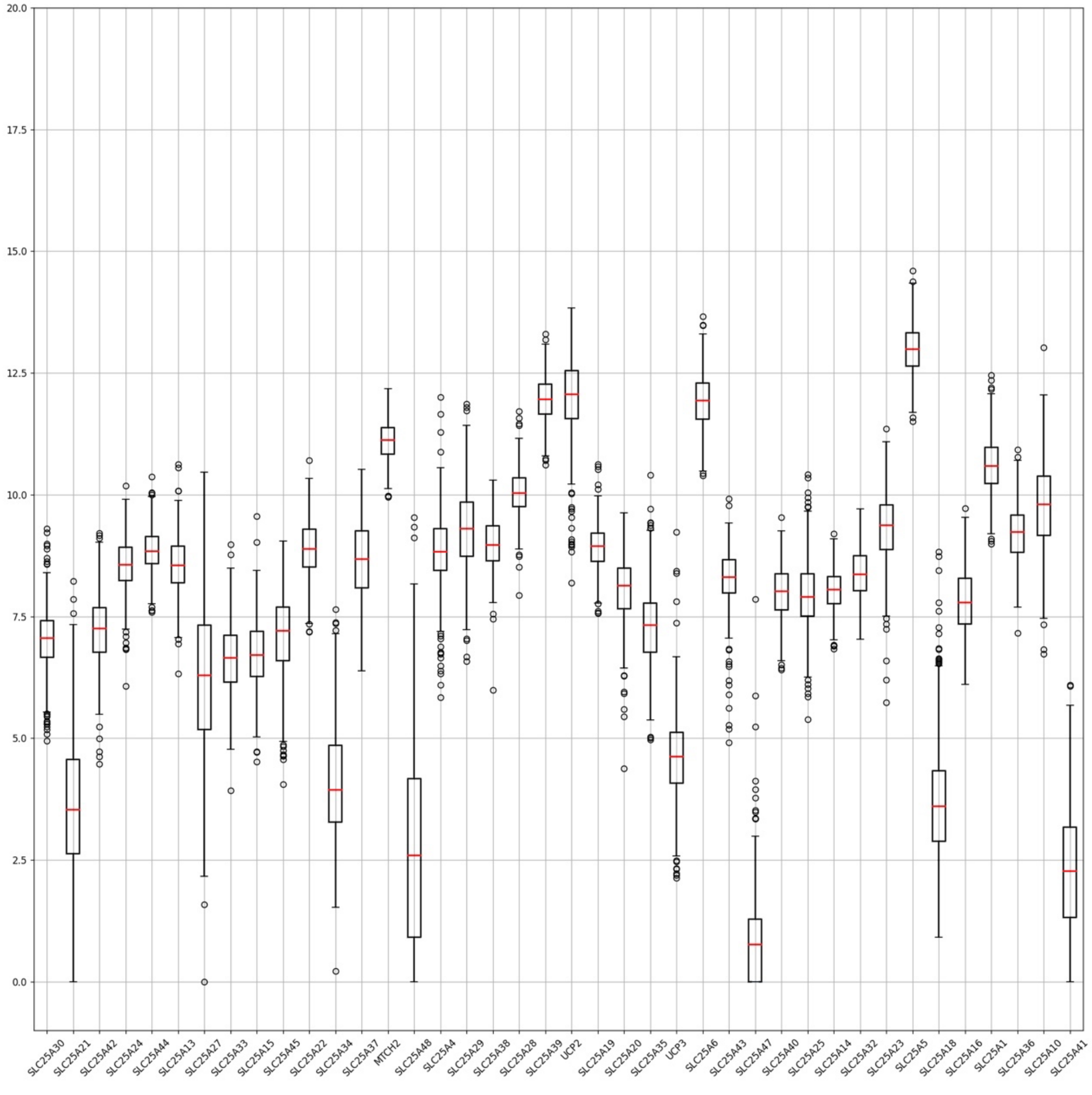

Figure S9D.

Boxplot of SLC25 transporter expression cluster 3 of TCGA tumors (log_2FPKM)

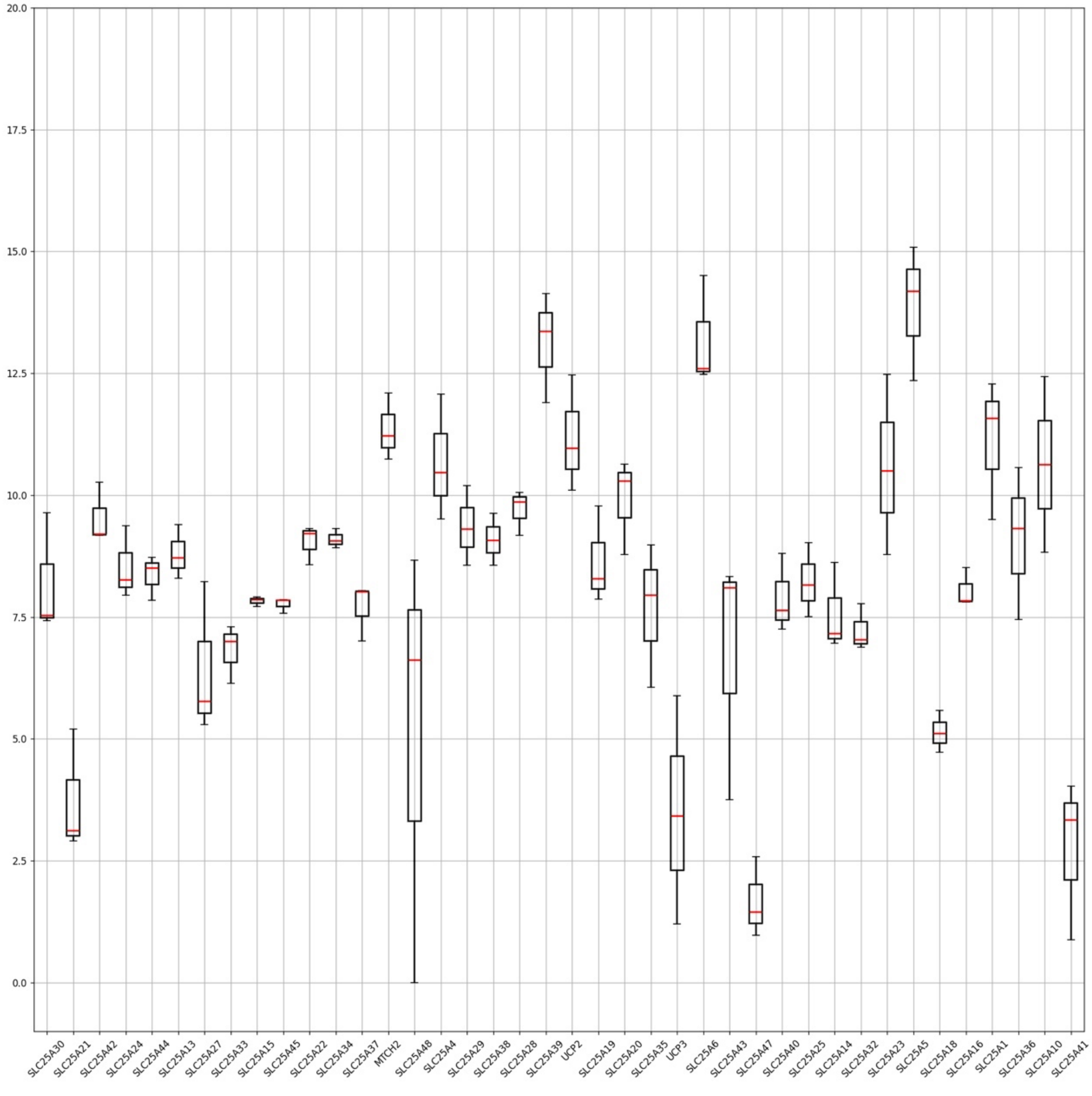

Figure S9E.

Boxplot of SLC25 transporter expression cluster 4 of TCGA tumors (log_2FPKM)

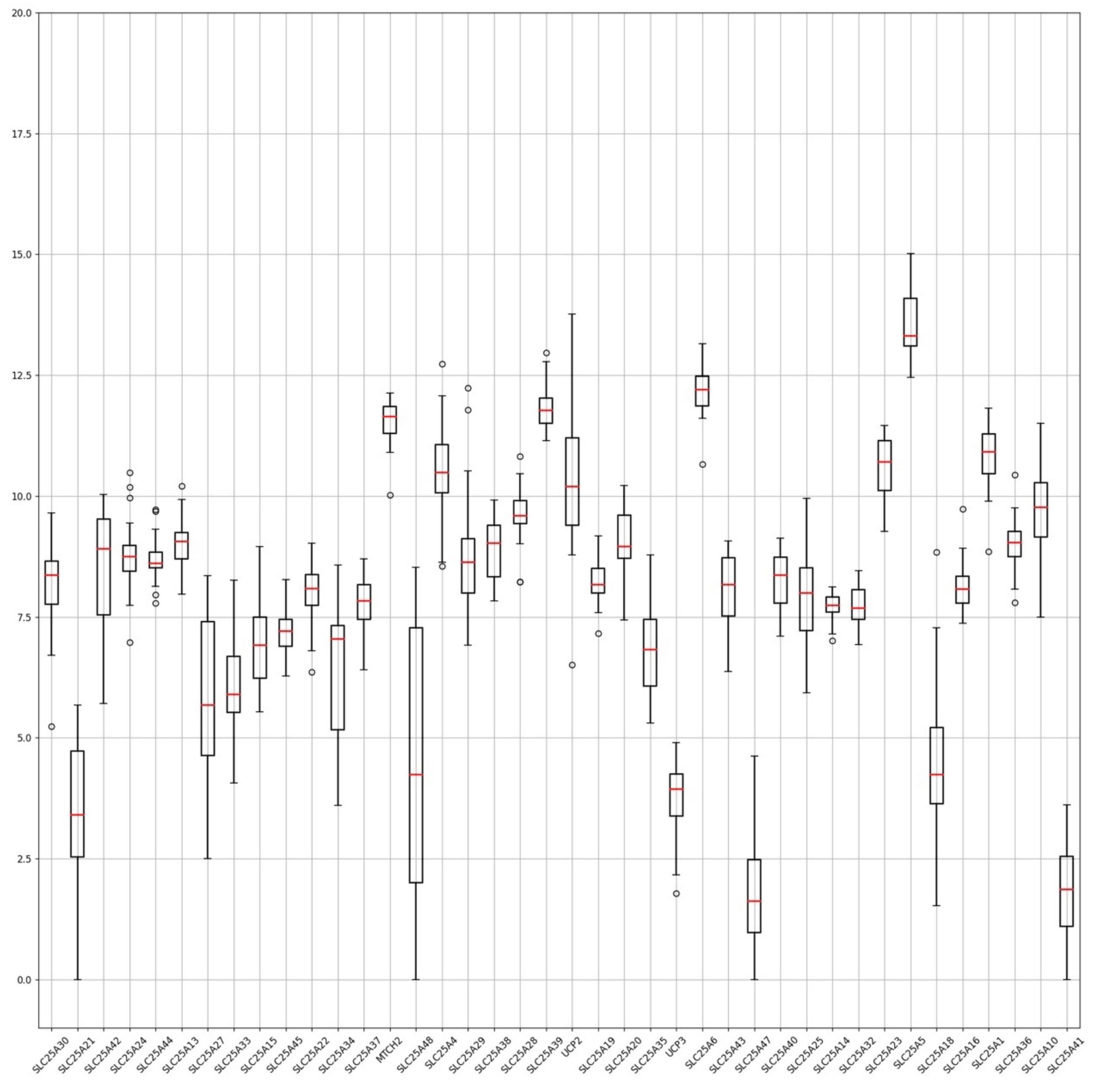

Figure S9F.

Boxplot of SLC25 transporter expression cluster 5 of TCGA tumors (log_2FPKM)

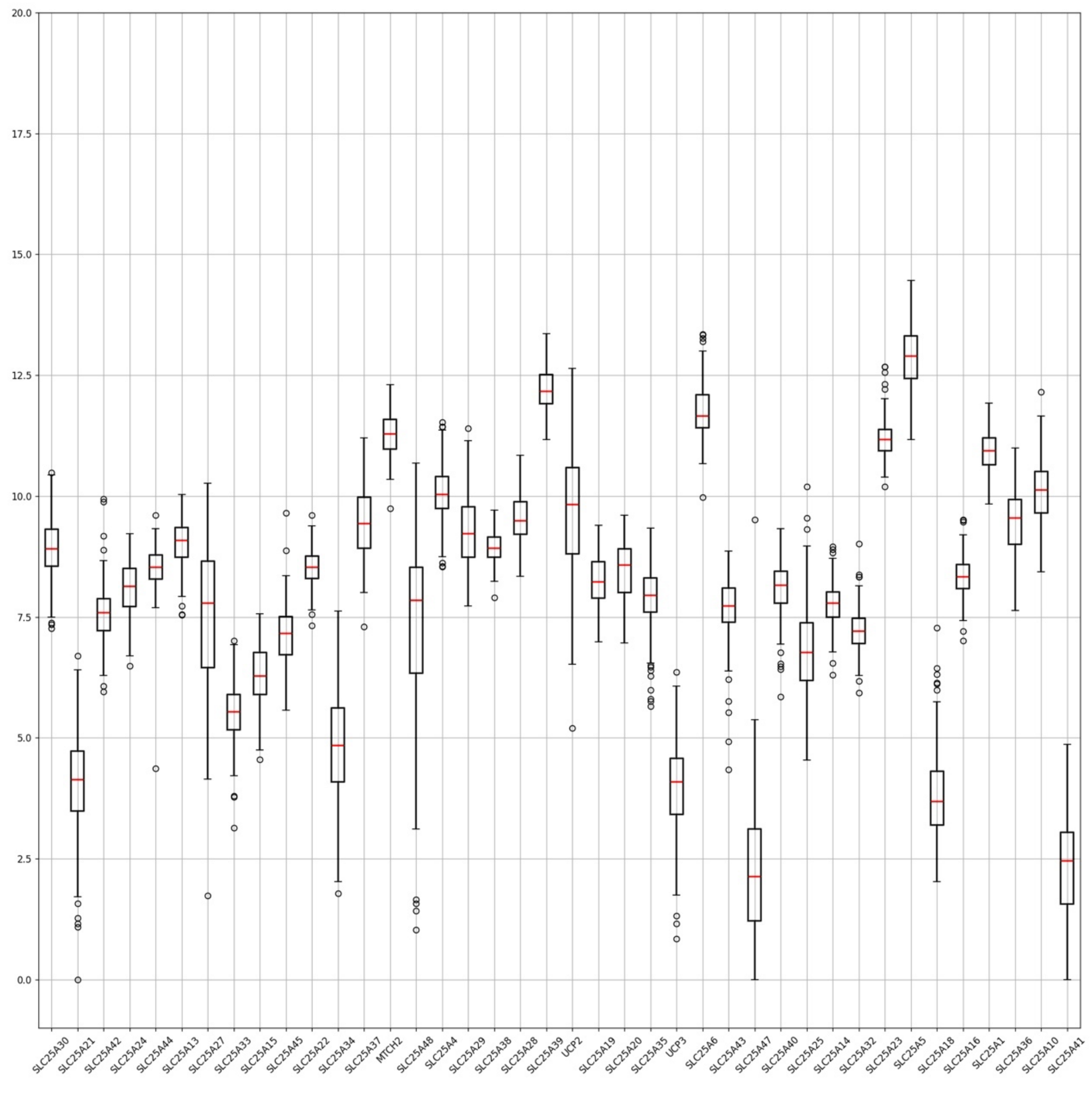

Figure S9G.

Boxplot of SLC25 transporter expression cluster 6 of TCGA tumors (log_2FPKM)

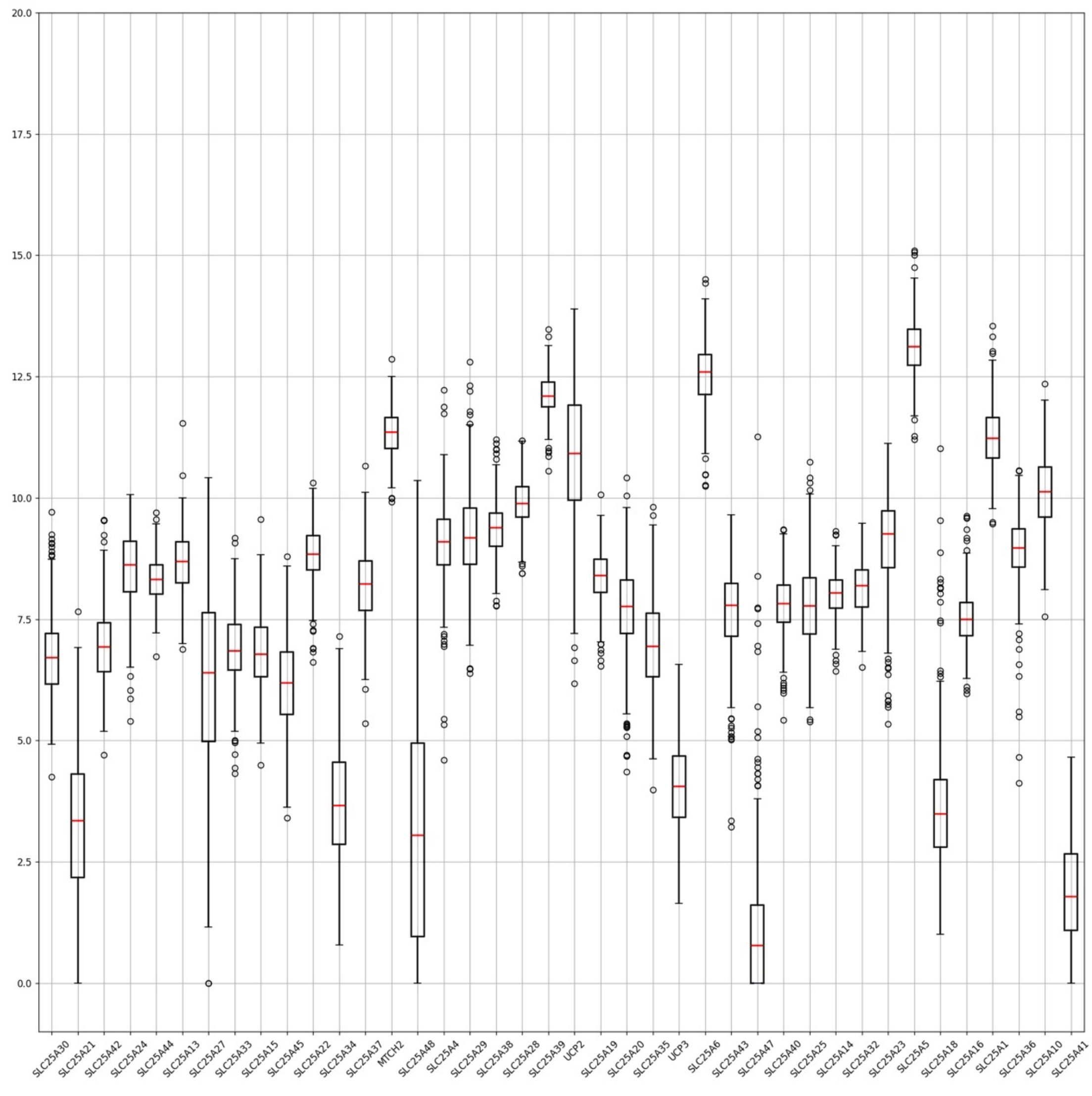

Figure S9H.

Boxplot of SLC25 transporter expression cluster 7 of TCGA tumors (log_2FPKM)

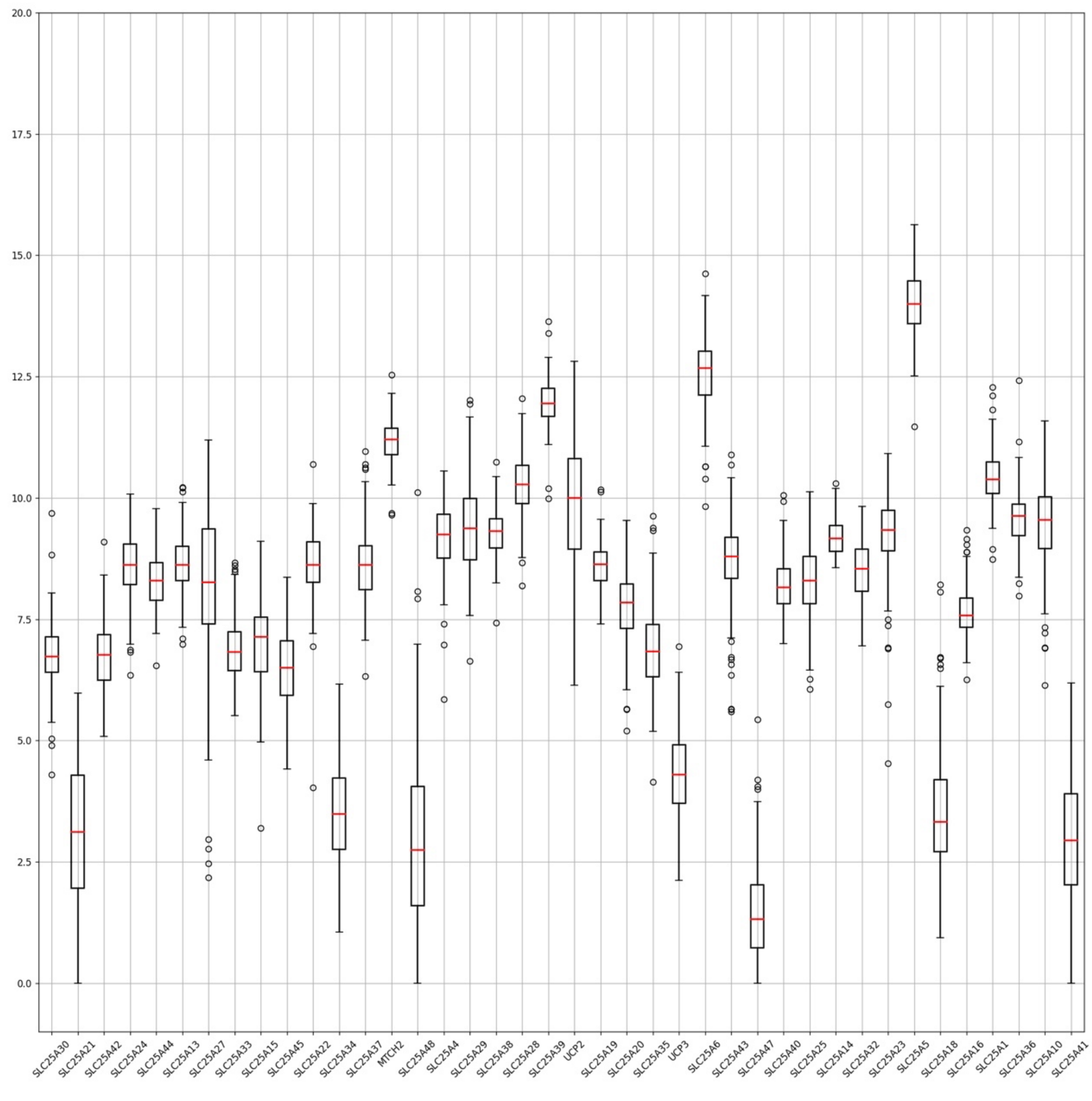

Figure S9I.

Boxplot of SLC25 transporter expression cluster 8 of TCGA tumors (log_2FPKM)

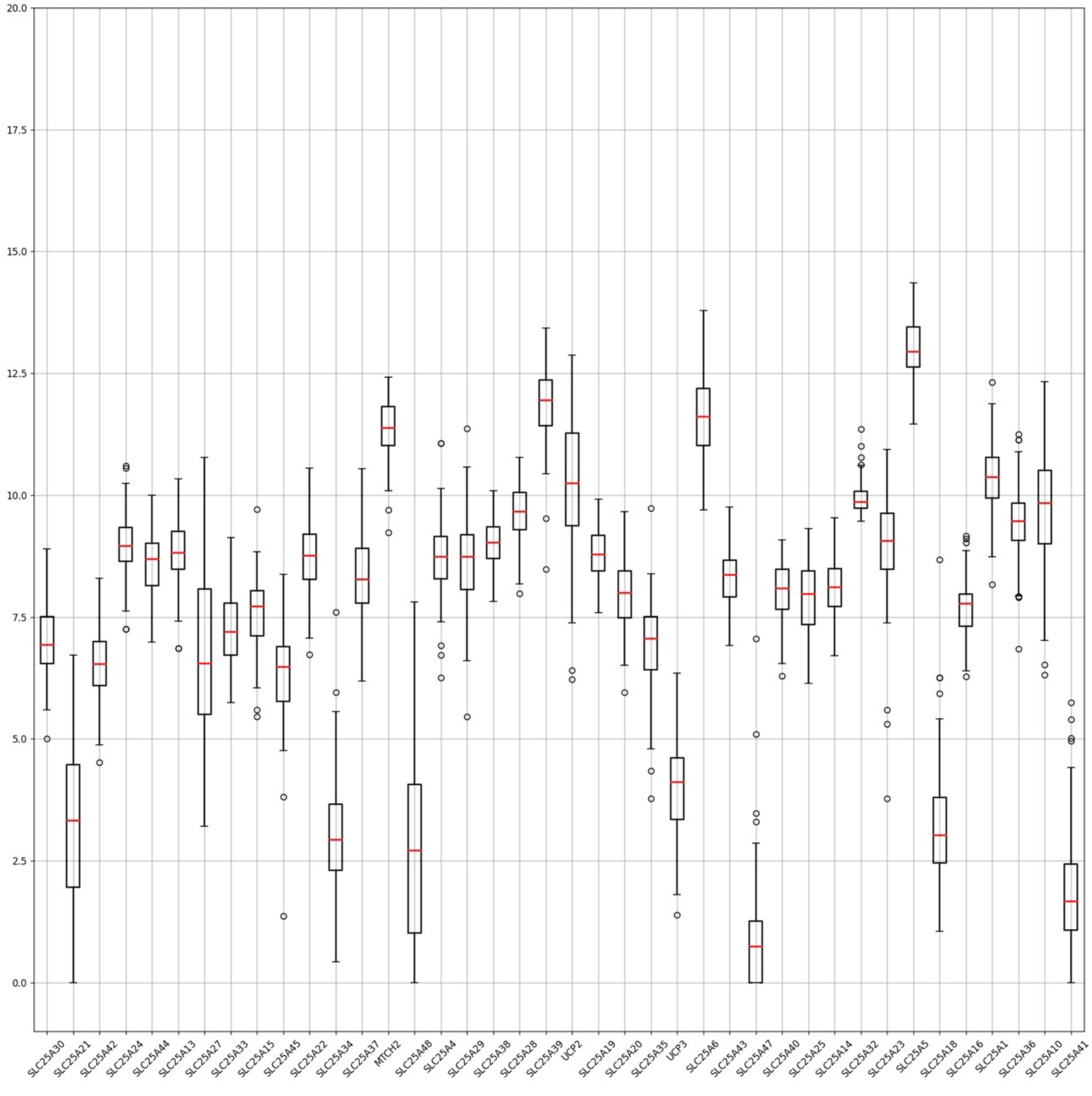

Figure S9J.

Boxplot of SLC25 transporter expression cluster 9 of TCGA tumors (log_2FPKM)

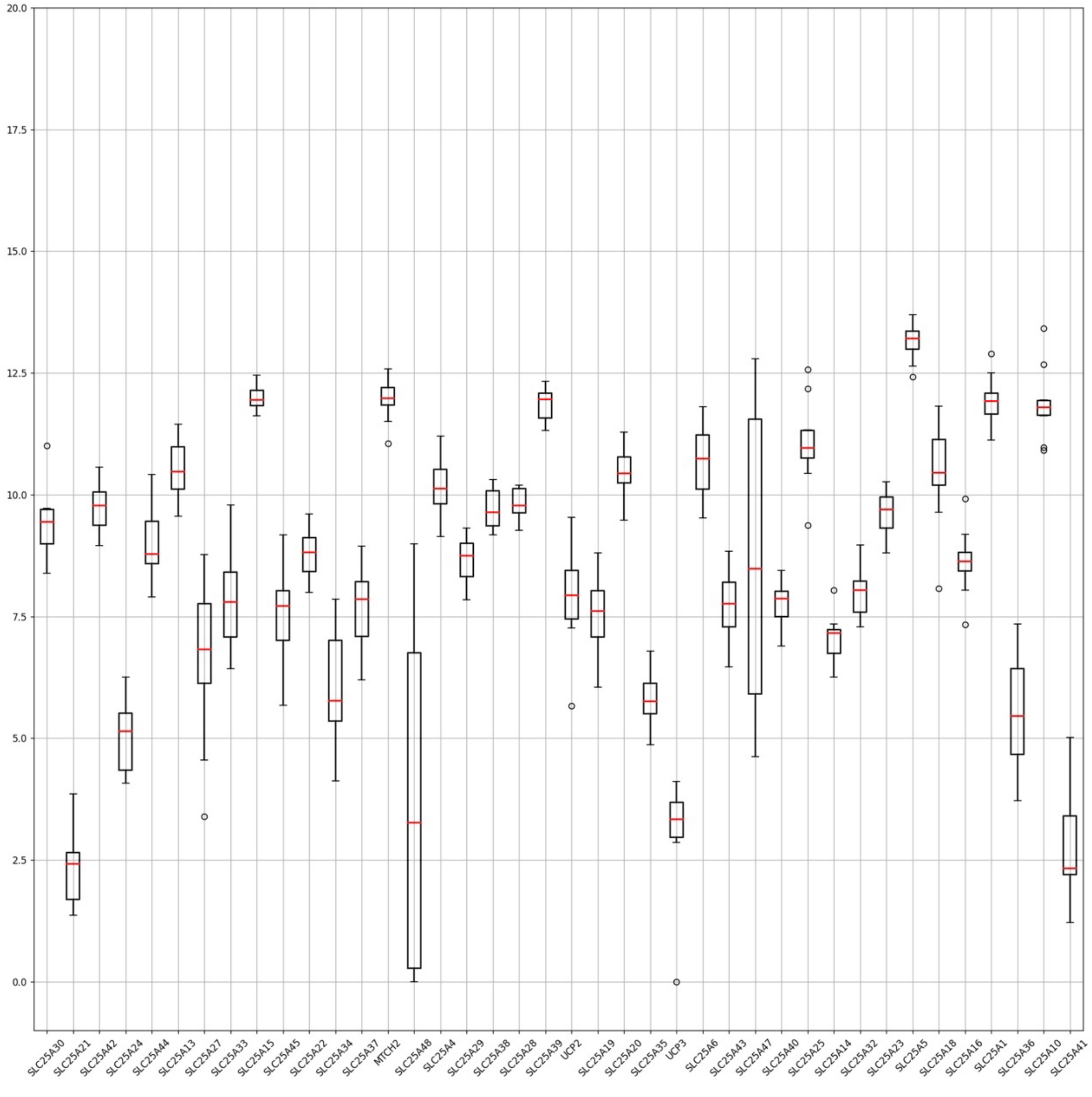

Figure S9K.

Boxplot of SLC25 transporter expression cluster 10 of TCGA tumors (log_2FPKM)

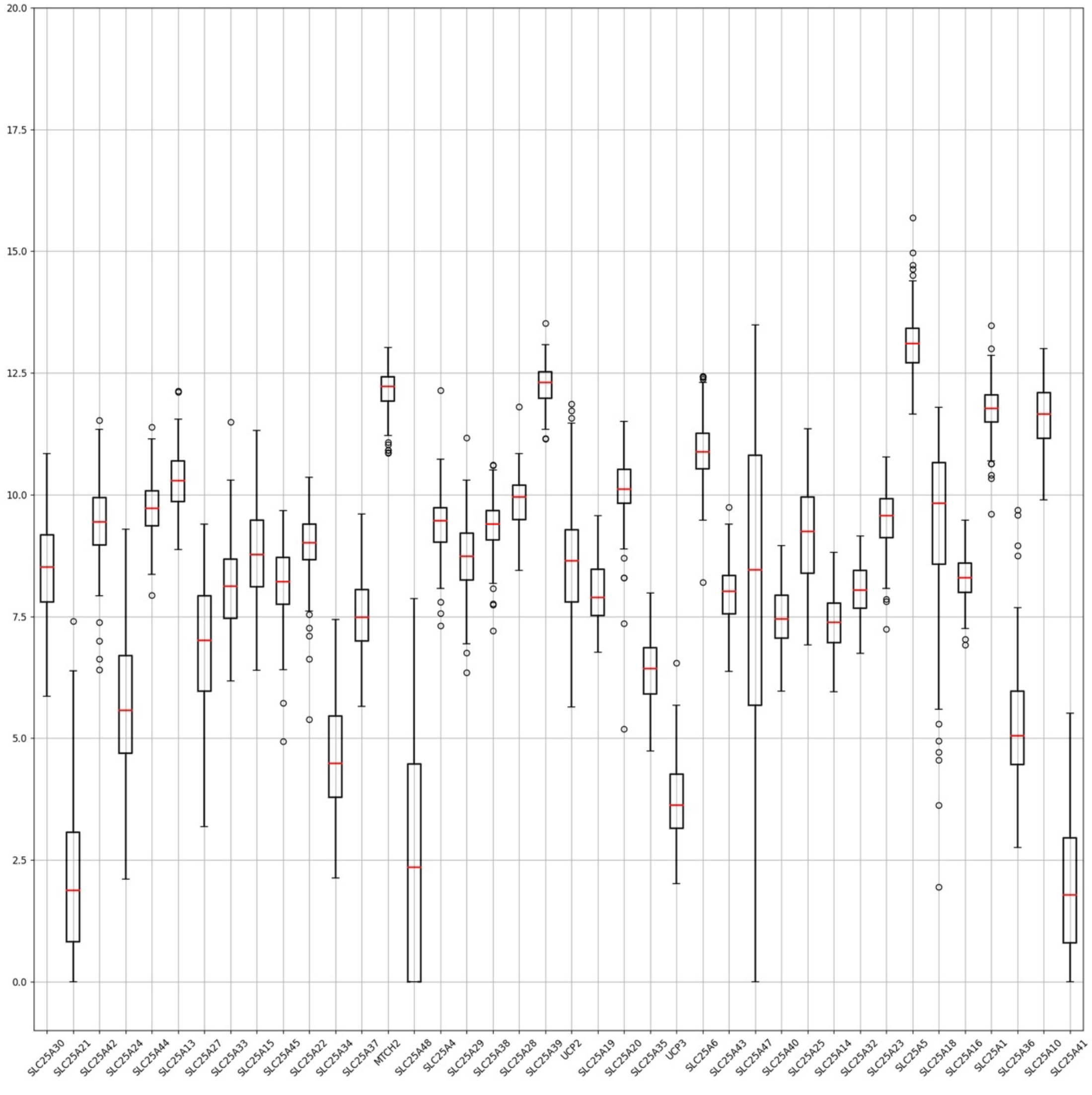

Figure S9L.

Boxplot of SLC25 transporter expression cluster 11 of TCGA tumors ($\log_2$FPKM)

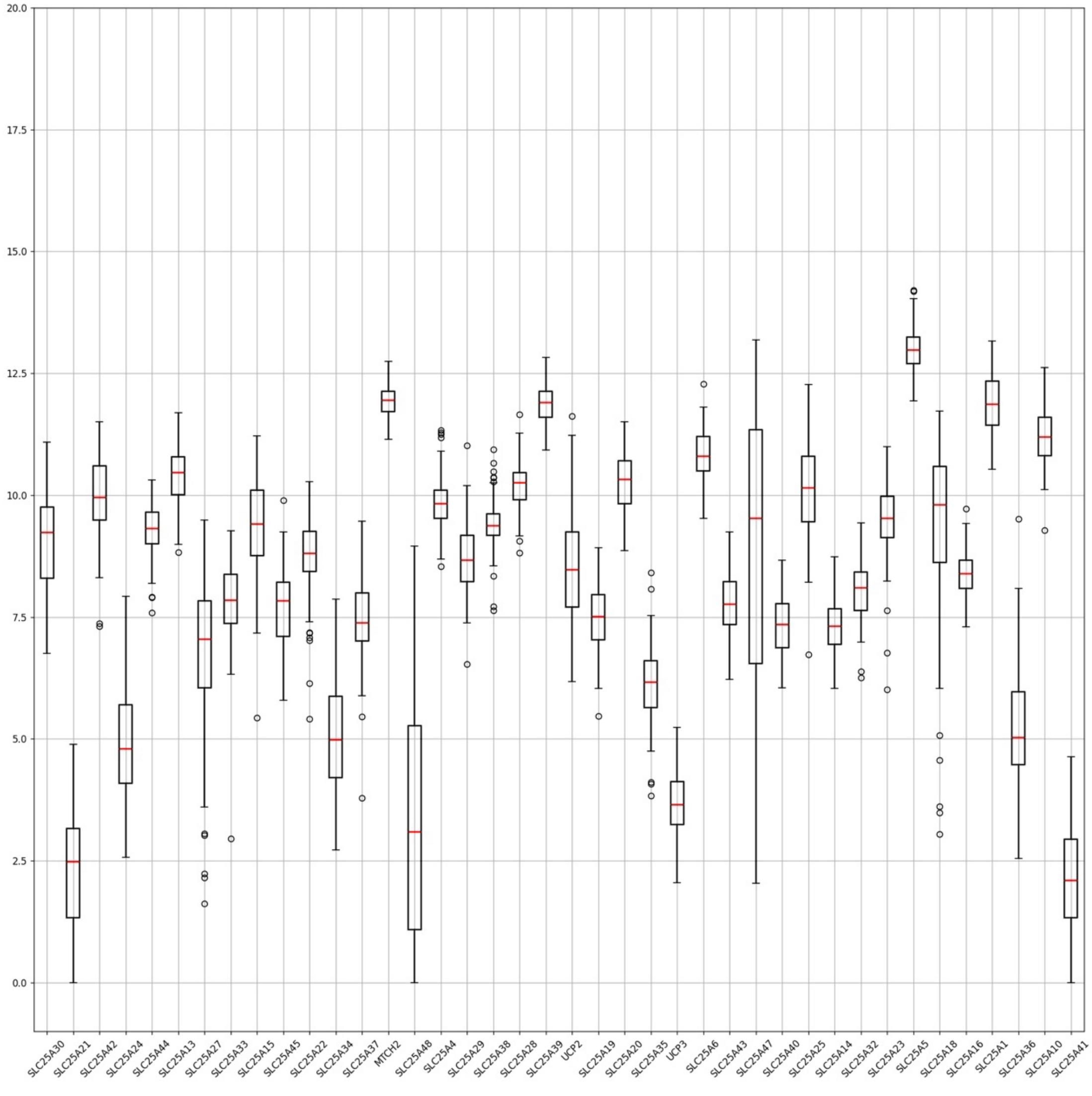

Figure S9M.

Boxplot of SLC25 transporter expression cluster 12 of TCGA tumors (log_2FPKM)

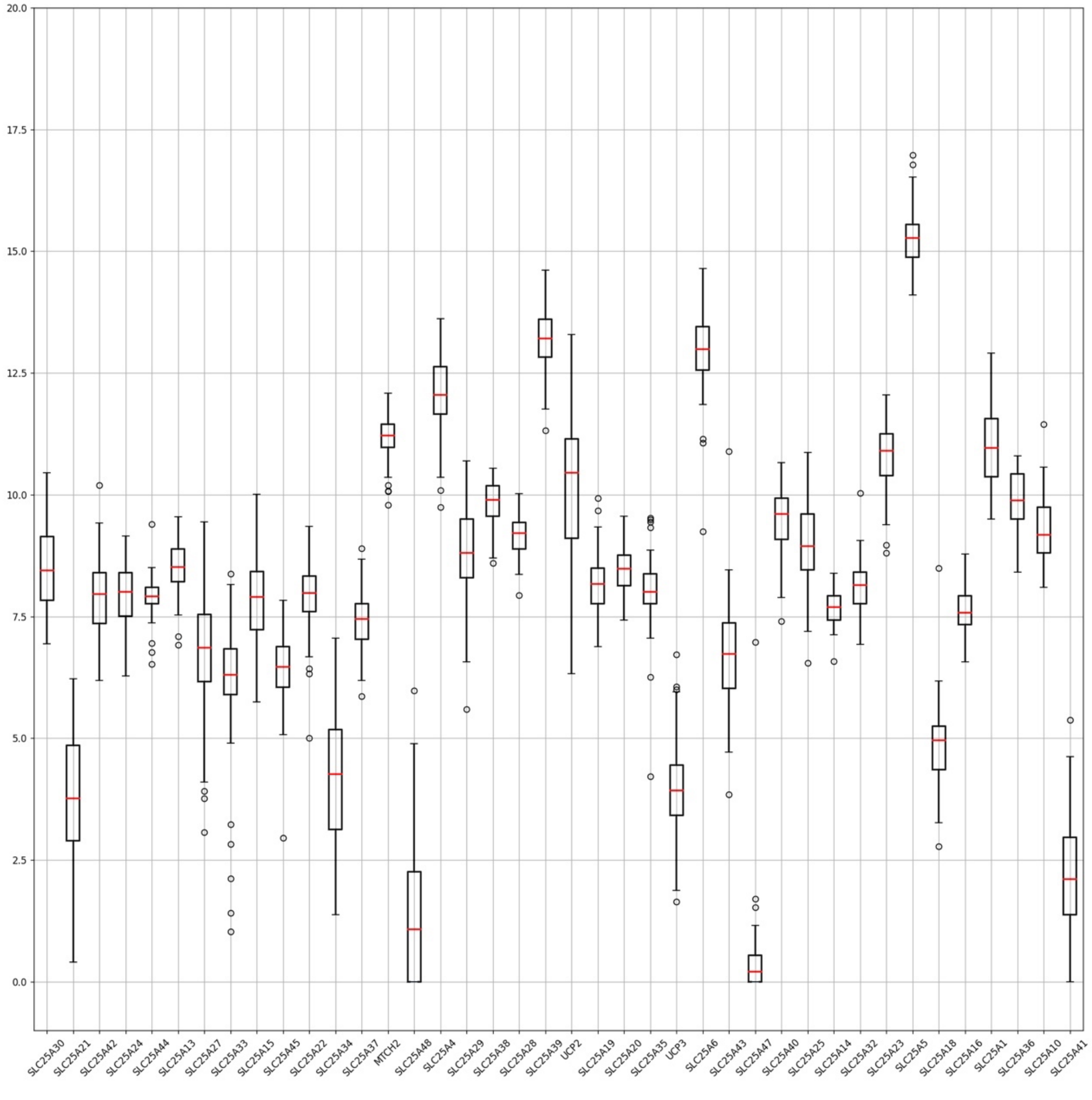

Figure S9N.

Boxplot of SLC25 transporter expression cluster 13 of TCGA tumors (log_2FPKM)

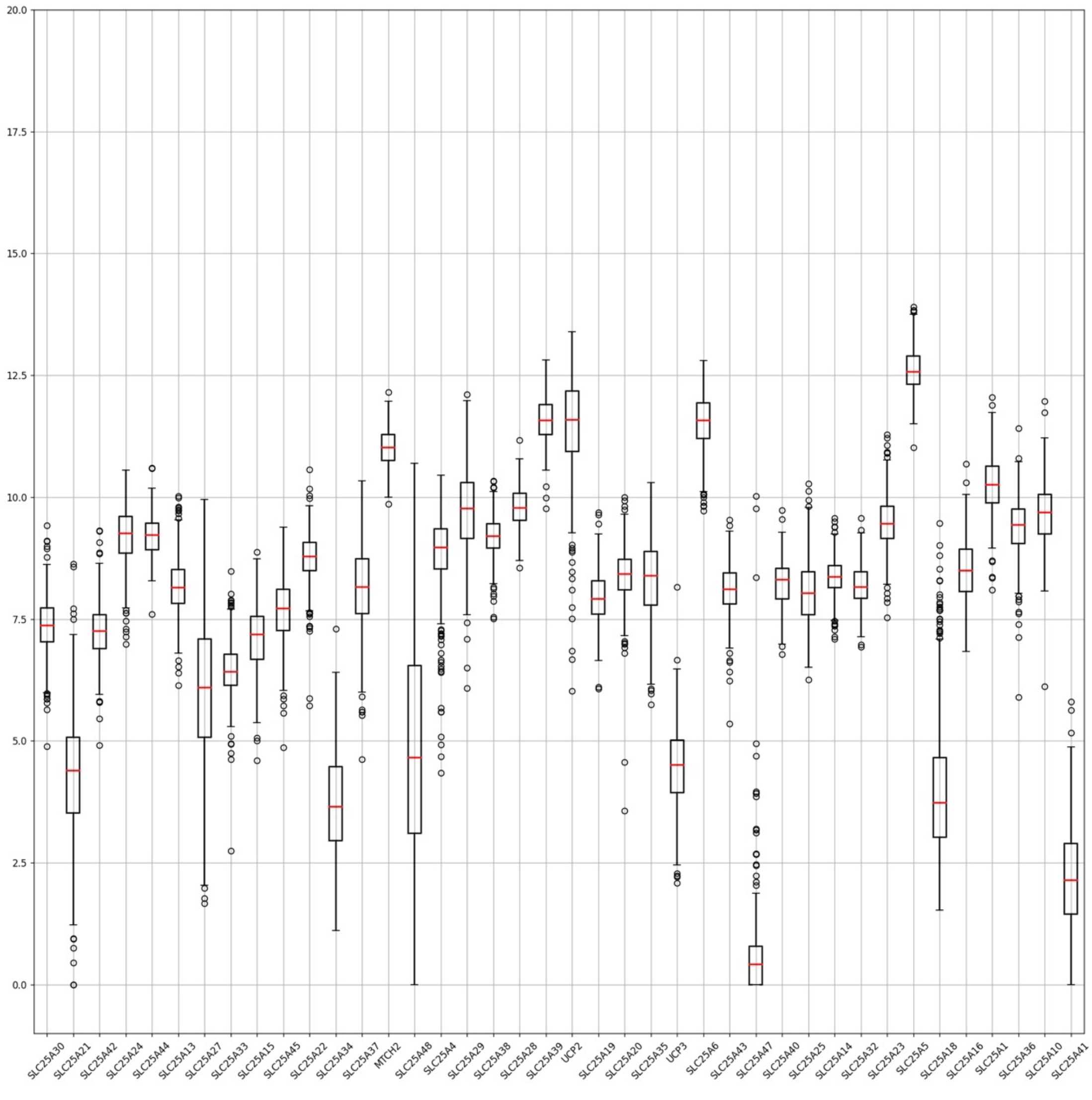

Figure S9O.

Boxplot of SLC25 transporter expression cluster 14 of TCGA tumors (log_2FPKM)

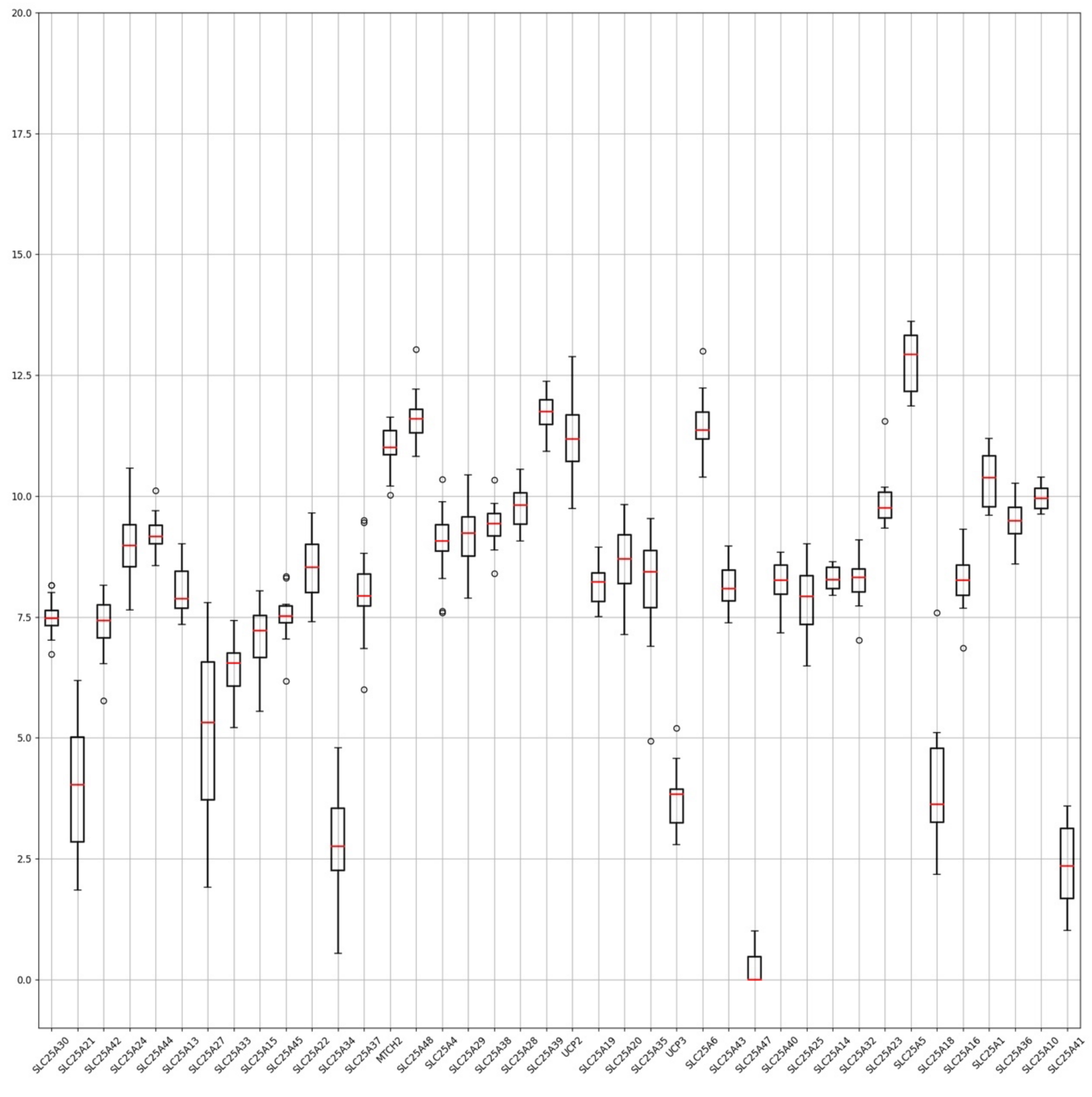

Figure S9P.

Boxplot of SLC25 transporter expression cluster 15 of TCGA tumors (log_2FPKM)

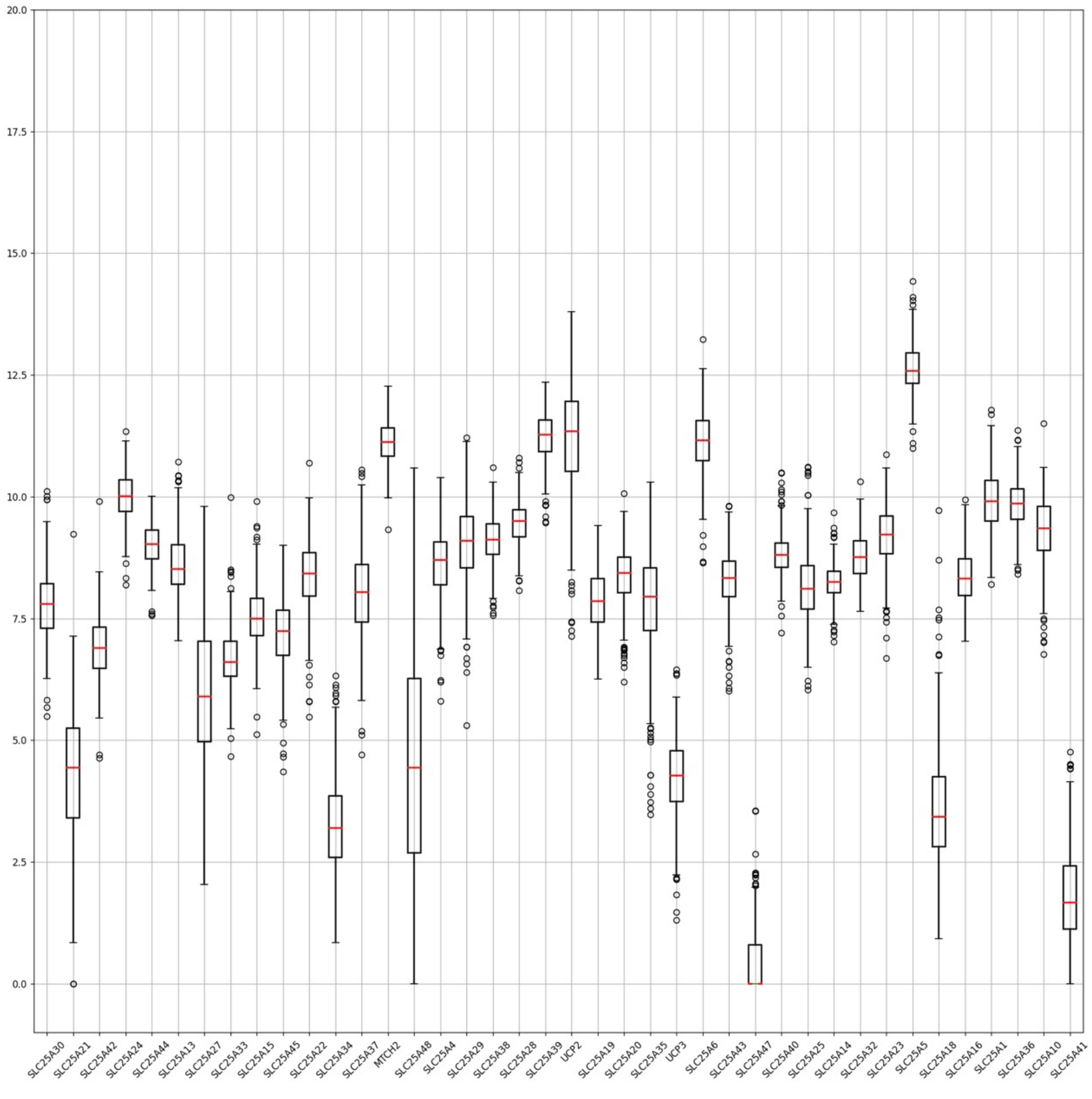

Figure S9Q.

Boxplot of SLC25 transporter expression cluster 16 of TCGA tumors (log_2FPKM)

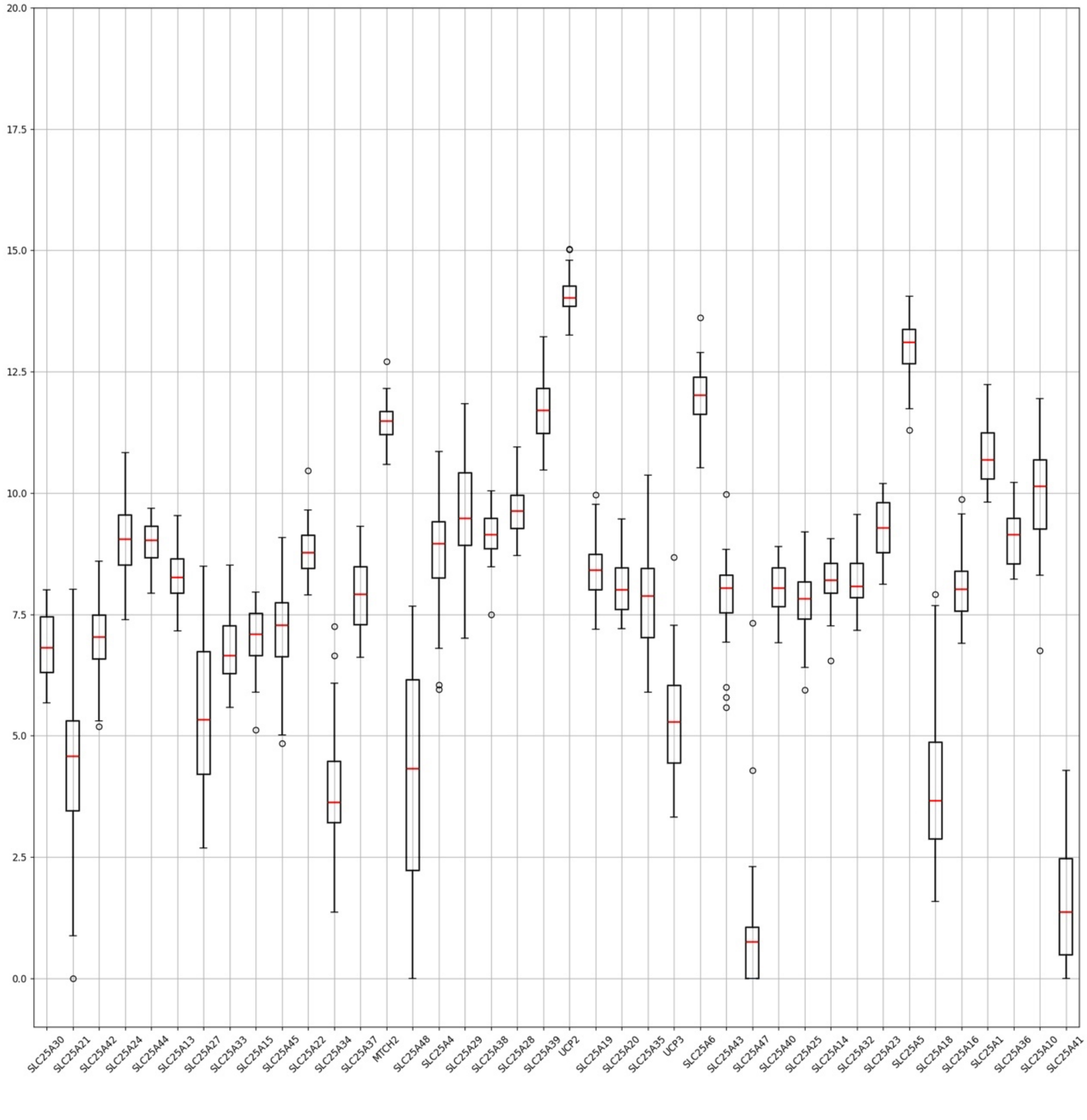

Figure S9R.

Boxplot of SLC25 transporter expression cluster 17 of TCGA tumors (log_2FPKM)

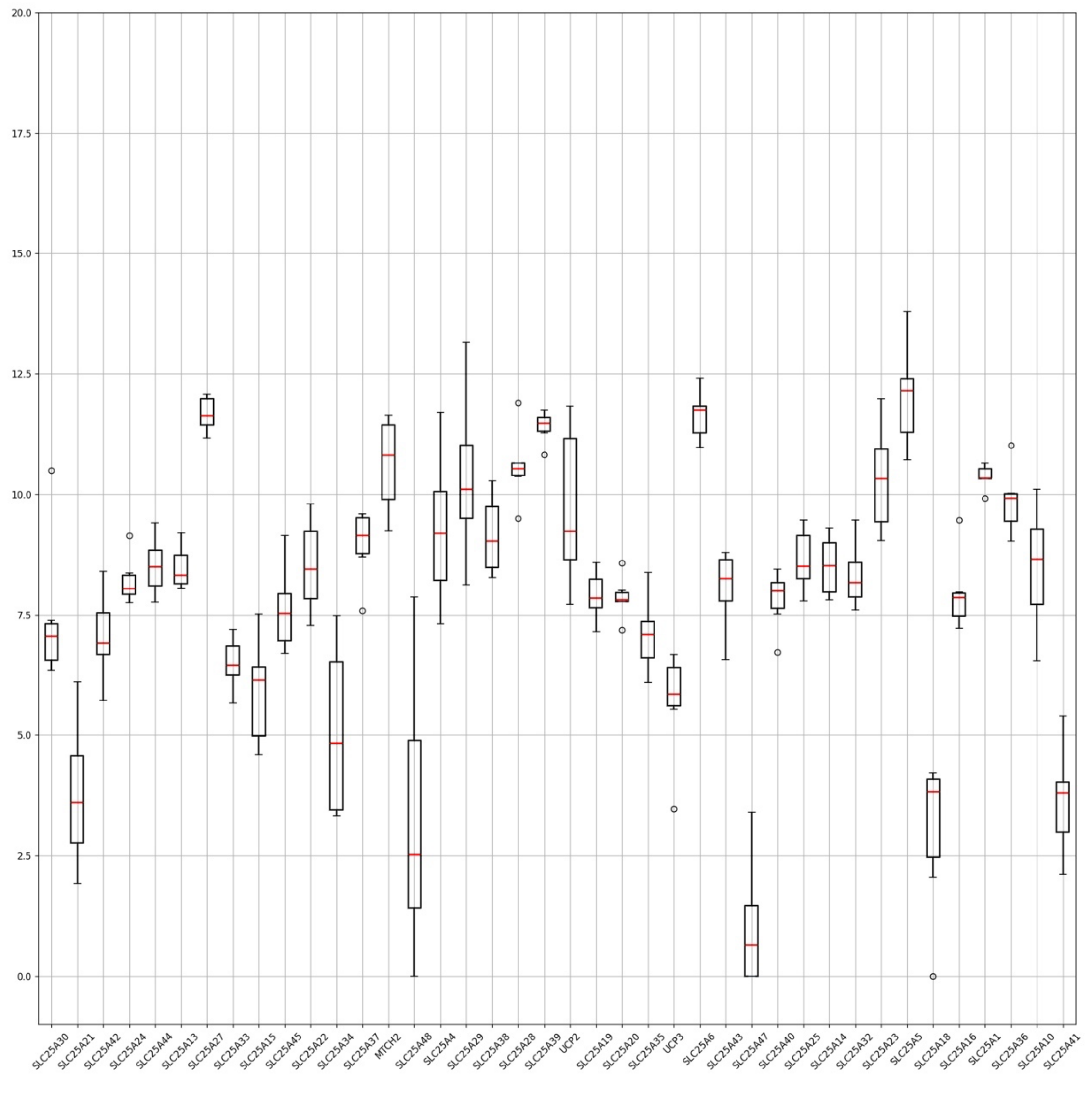

Figure S9S.

Boxplot of SLC25 transporter expression cluster 19 of TCGA tumors (log_2FPKM)

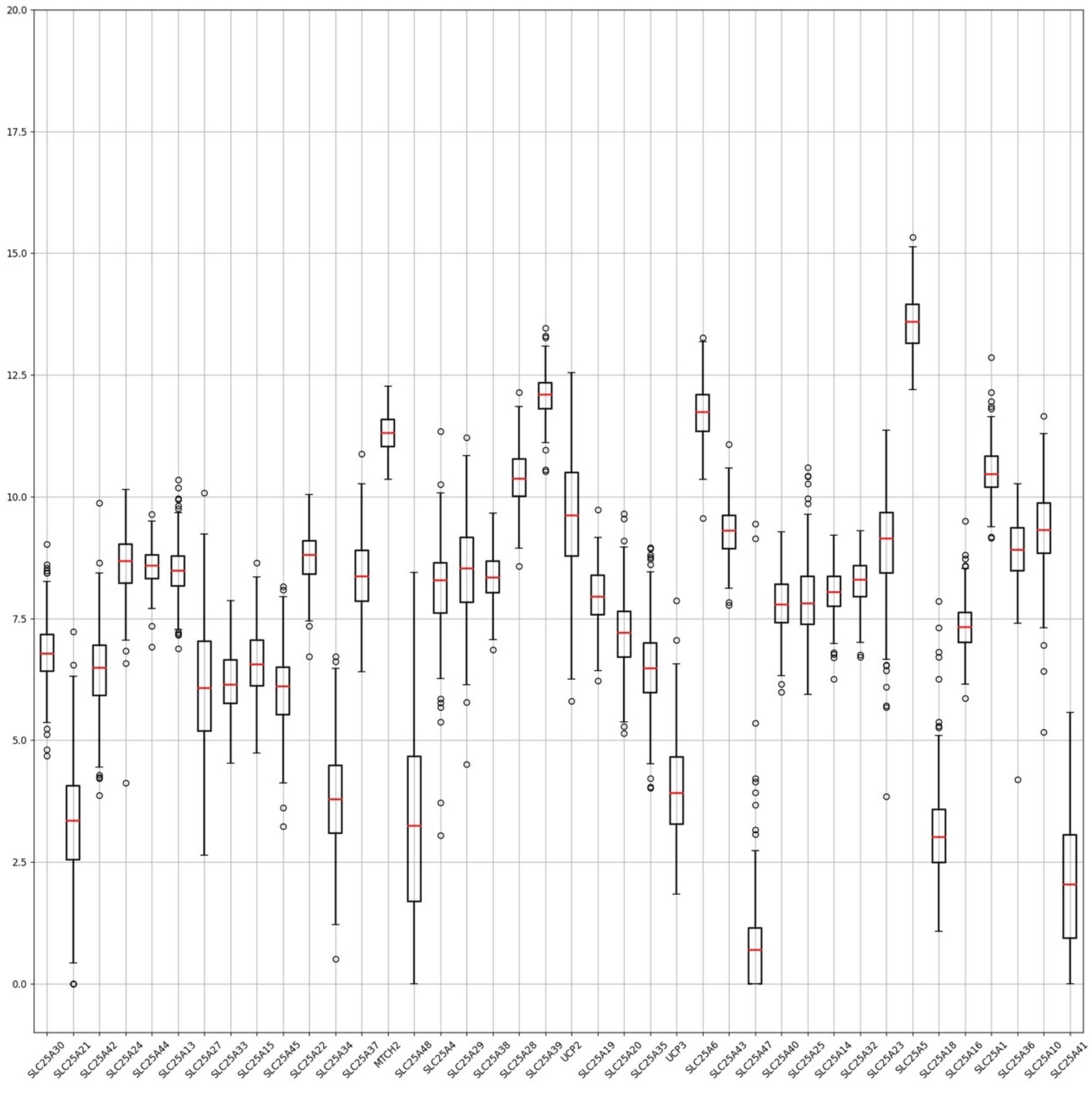

Figure S9T.

Boxplot of SLC25 transporter expression cluster 20 of TCGA tumors (log_2FPKM)

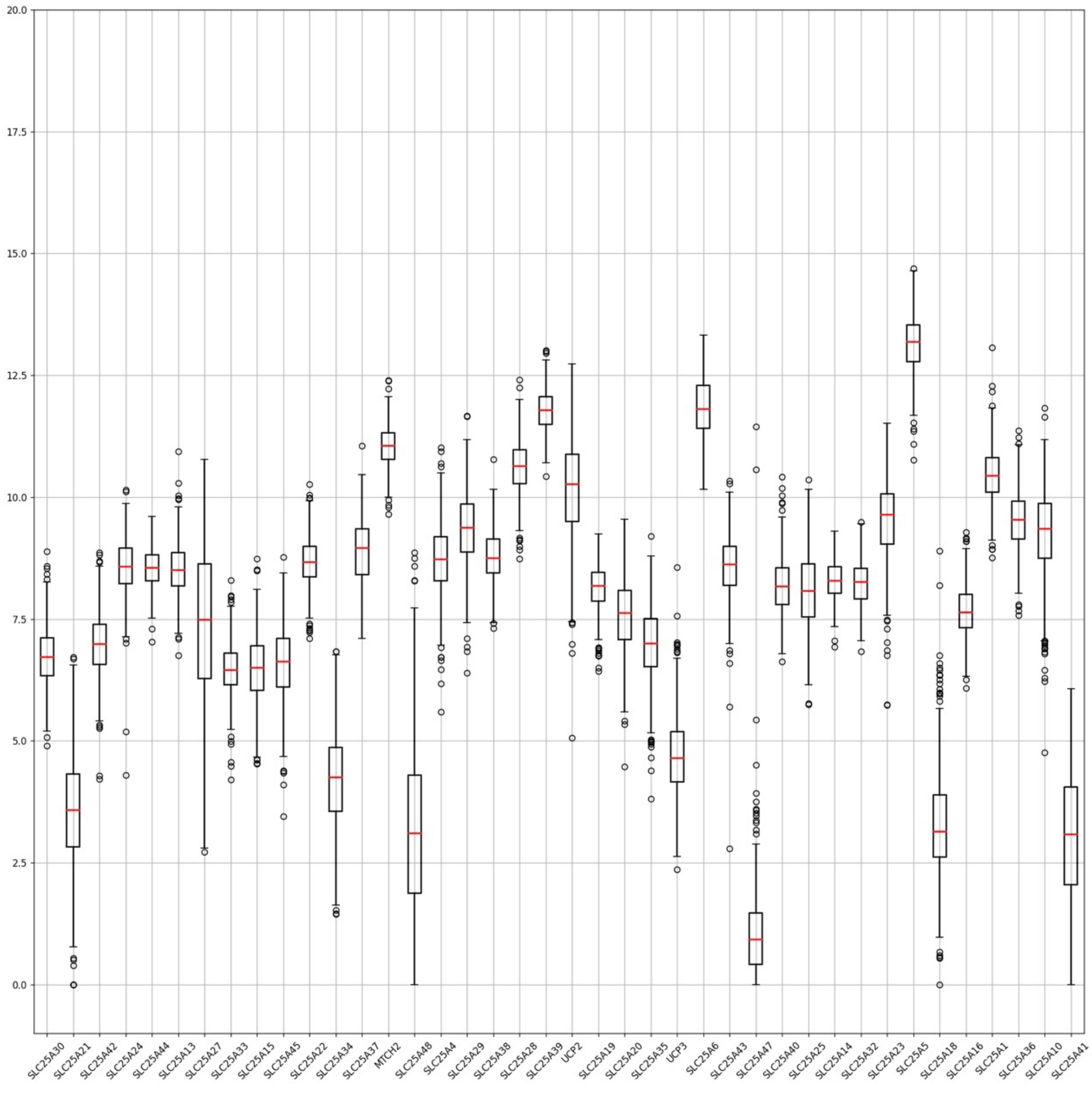

Figure S9U.

Boxplot of SLC25 transporter expression cluster 21 of TCGA tumors (log_2FPKM)

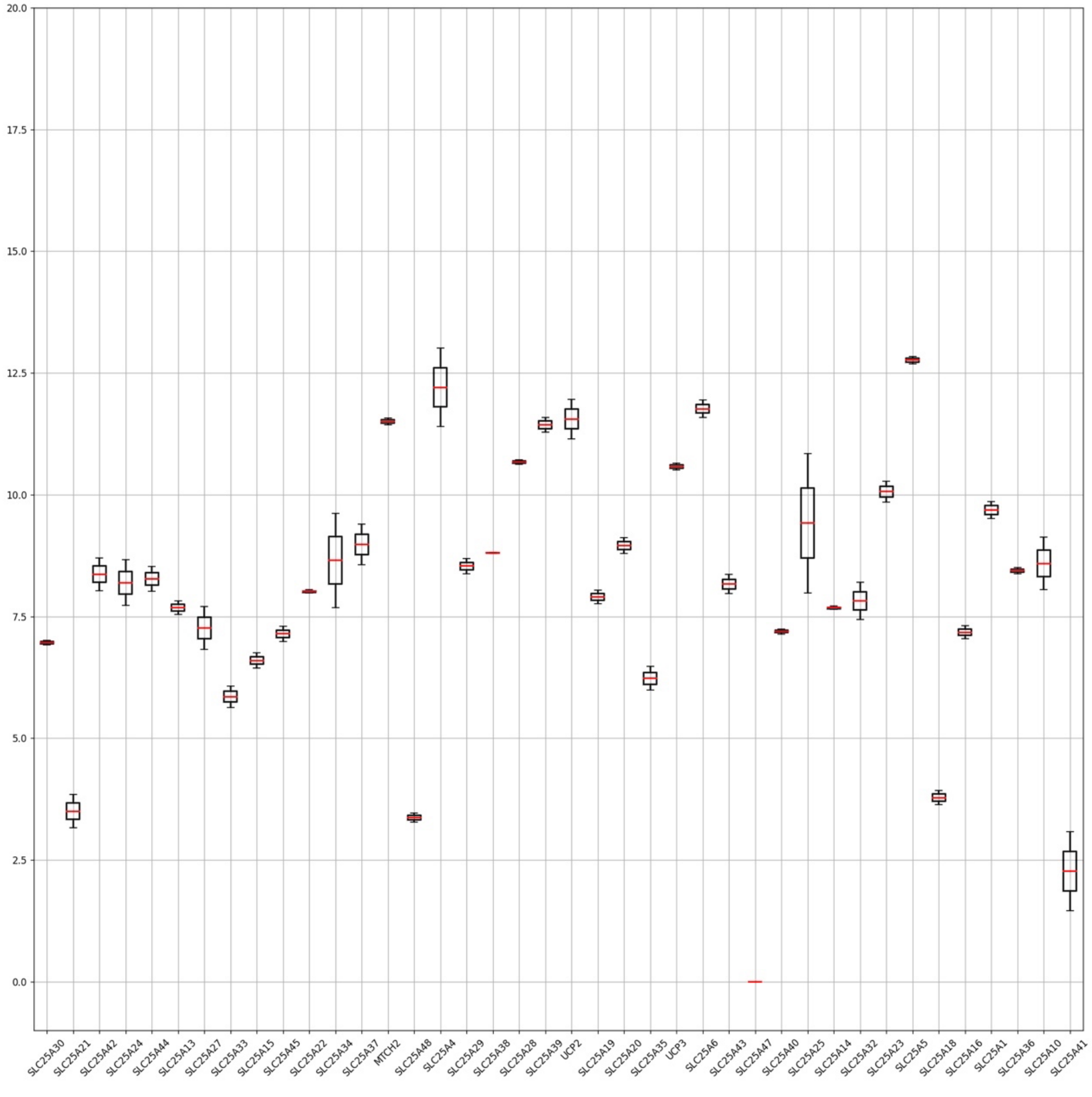

Figure S9V.

Boxplot of SLC25 transporter expression cluster 22 of TCGA tumors (log_2FPKM)

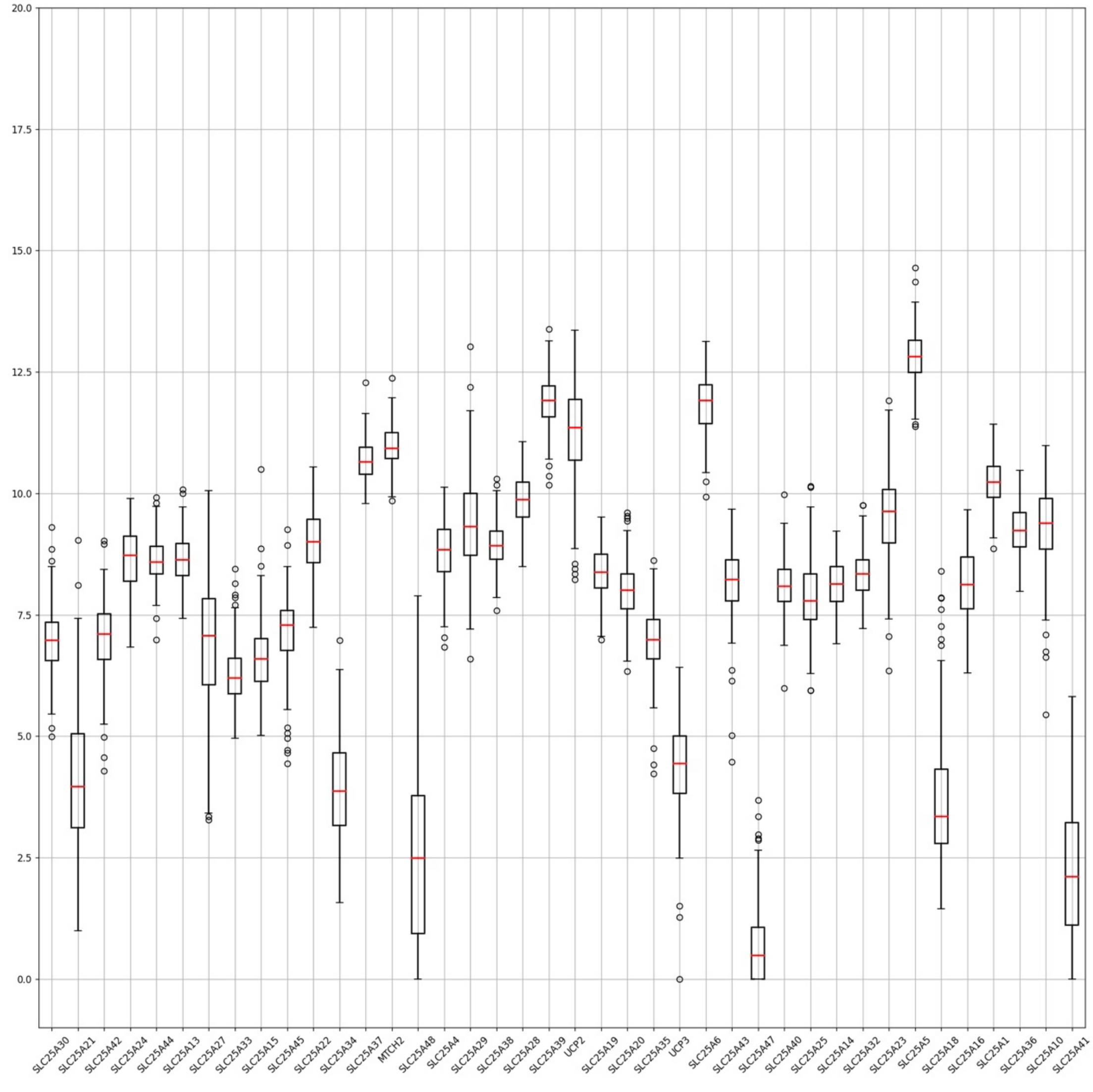

Figure S9W.

Boxplot of SLC25 transporter expression cluster 23 of TCGA tumors (log_2FPKM)

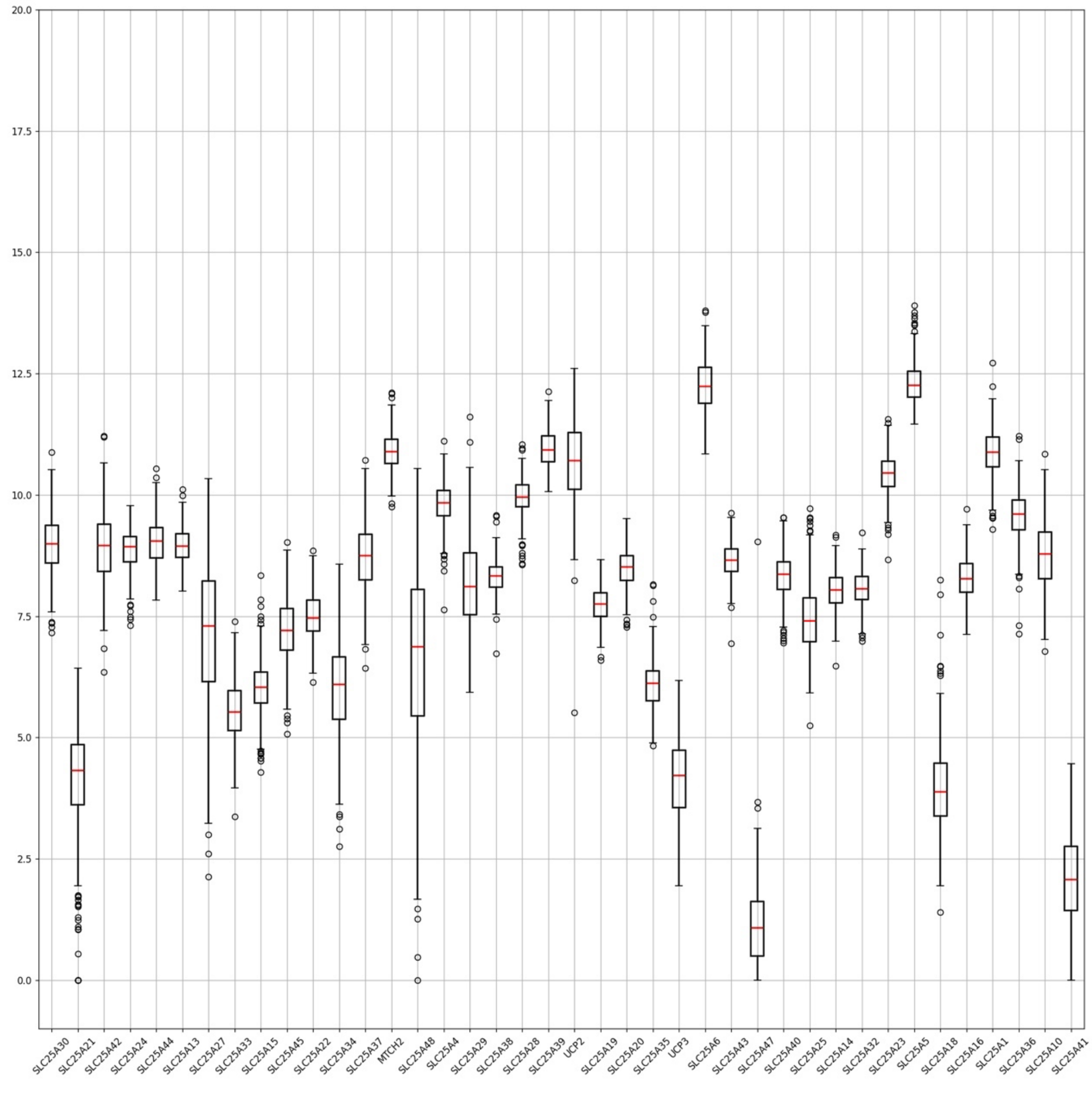

Figure S9X.

Boxplot of SLC25 transporter expression cluster 24 of TCGA tumors (log_2FPKM)

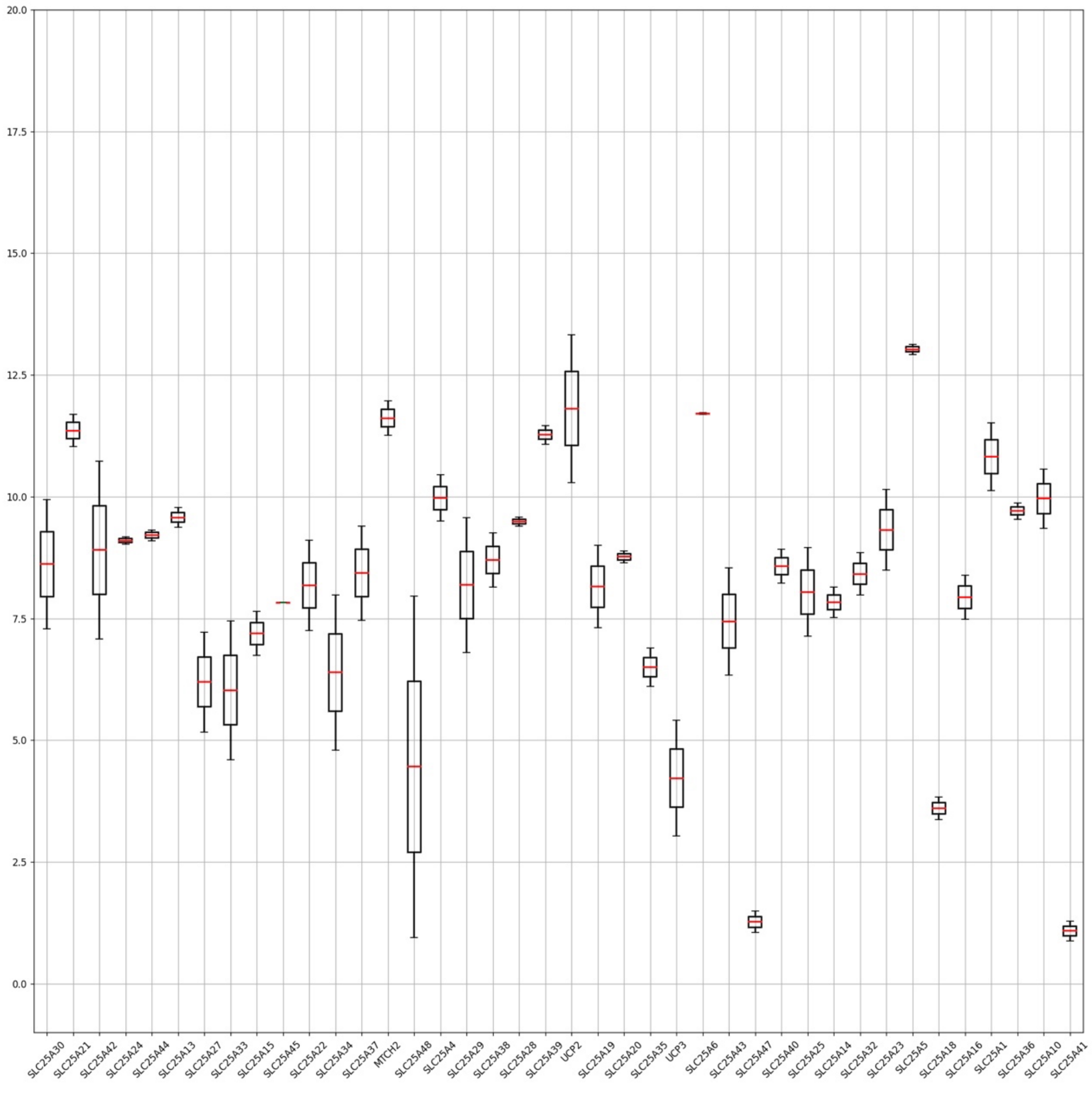

Figure S9Y.

Boxplot of SLC25 transporter expression cluster 25 of TCGA tumors (log_2FPKM)

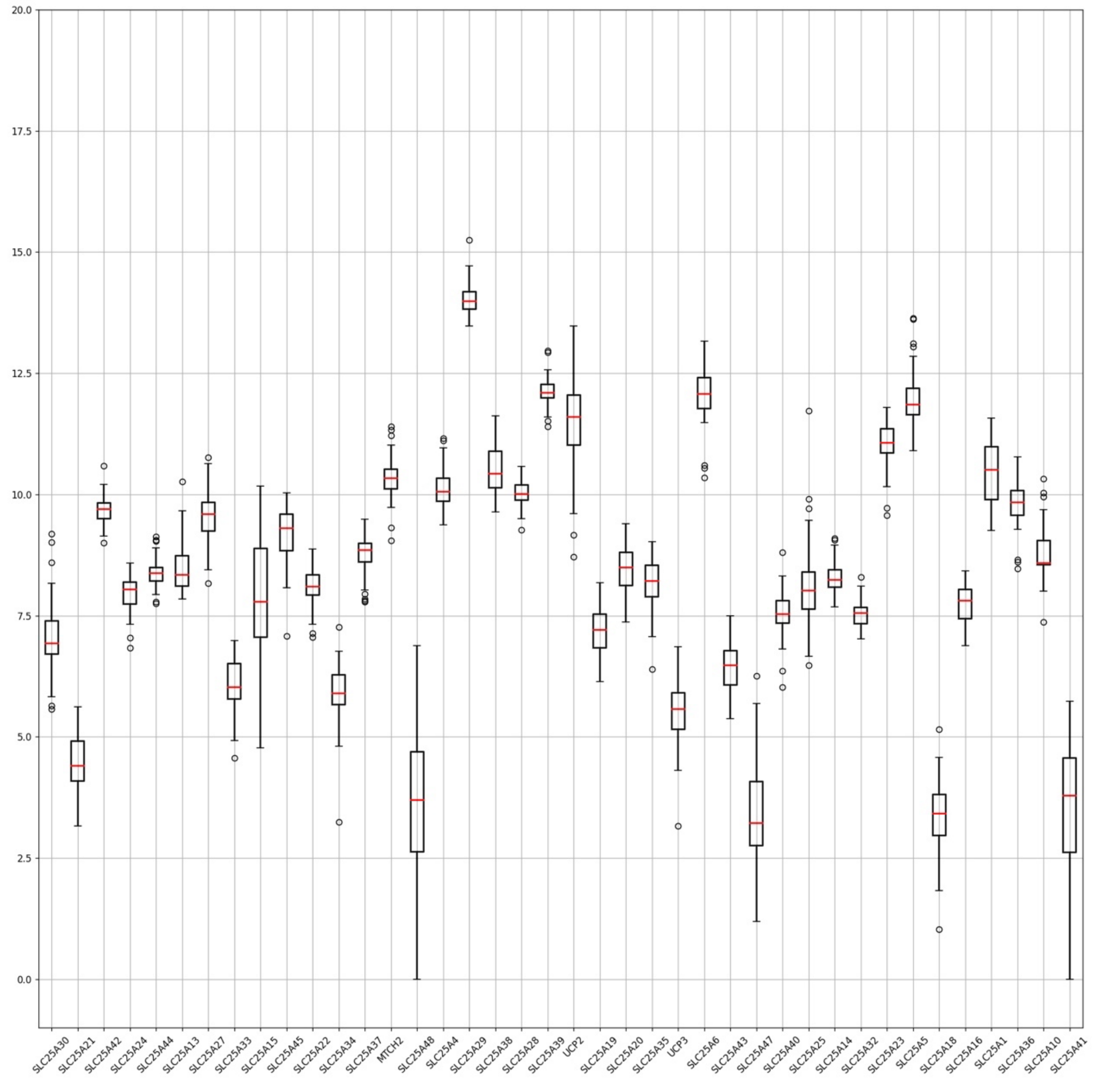

Figure S9Z.

Boxplot of SLC25 transporter expression cluster 26 of TCGA tumors (log_2FPKM)

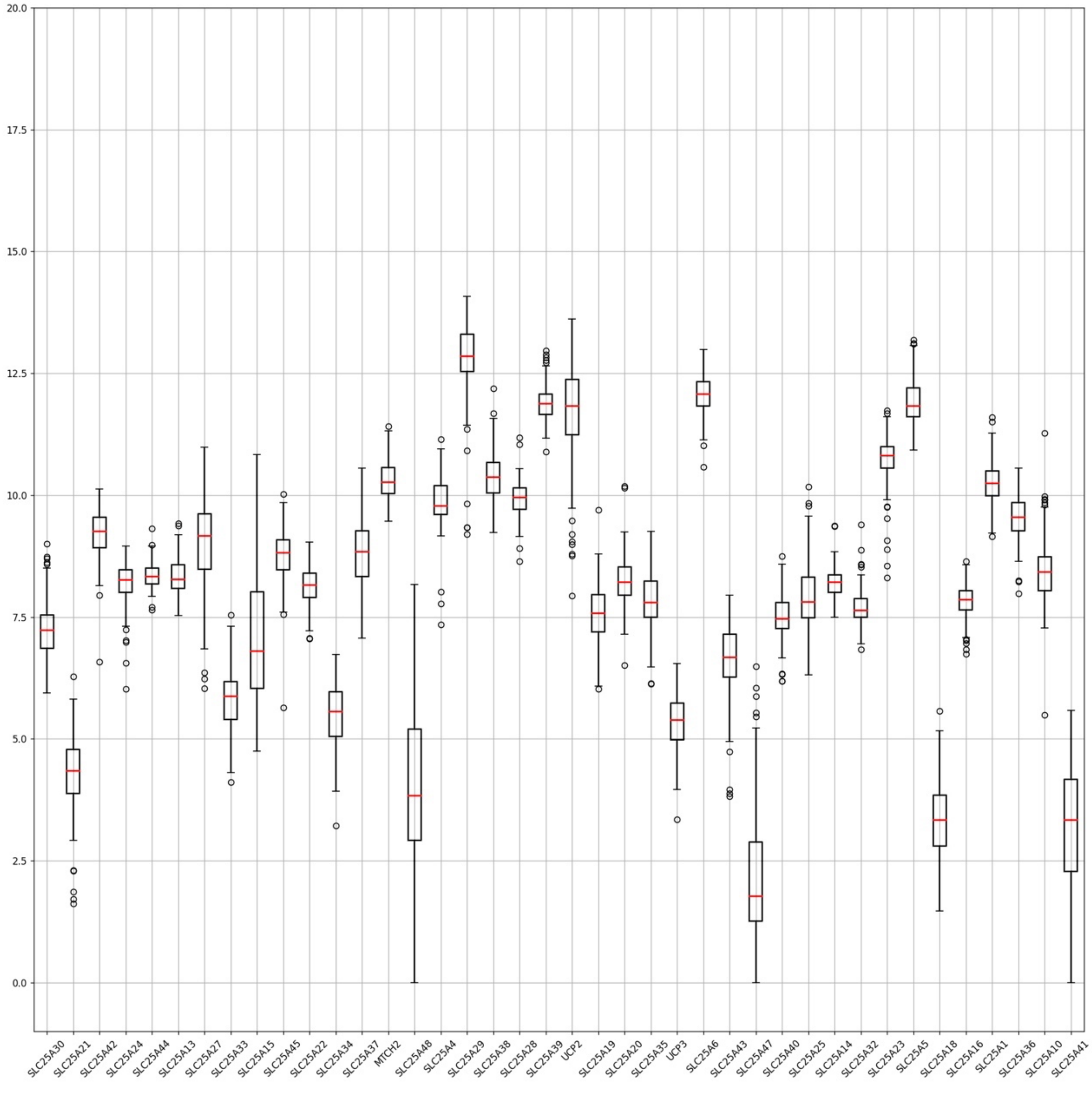

Figure S9AA.

Boxplot of SLC25 transporter expression cluster 27 of TCGA tumors (log_2FPKM)

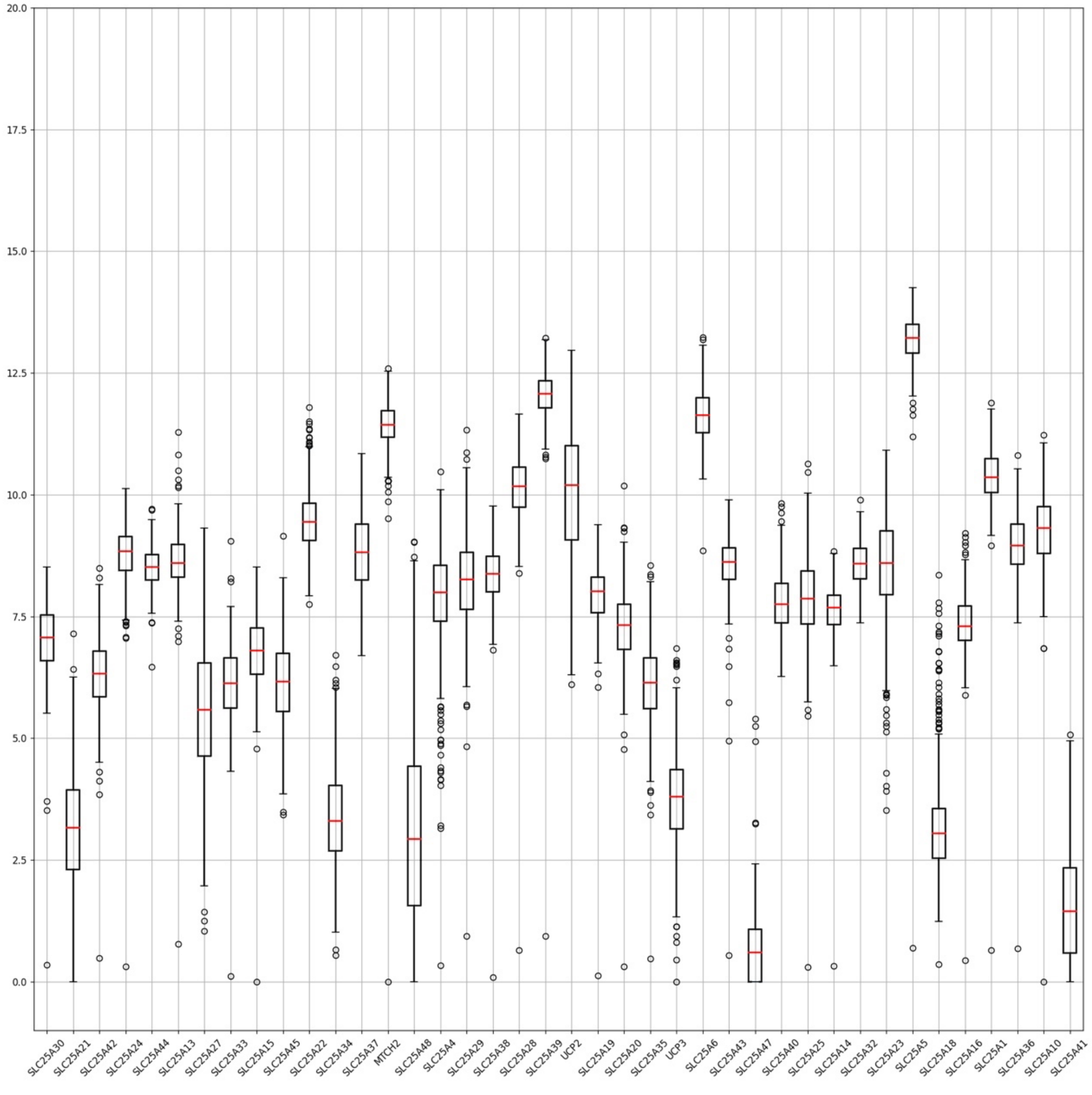

Figure S9AB.

Boxplot of SLC25 transporter expression cluster 31 of TCGA tumors (log_2FPKM)

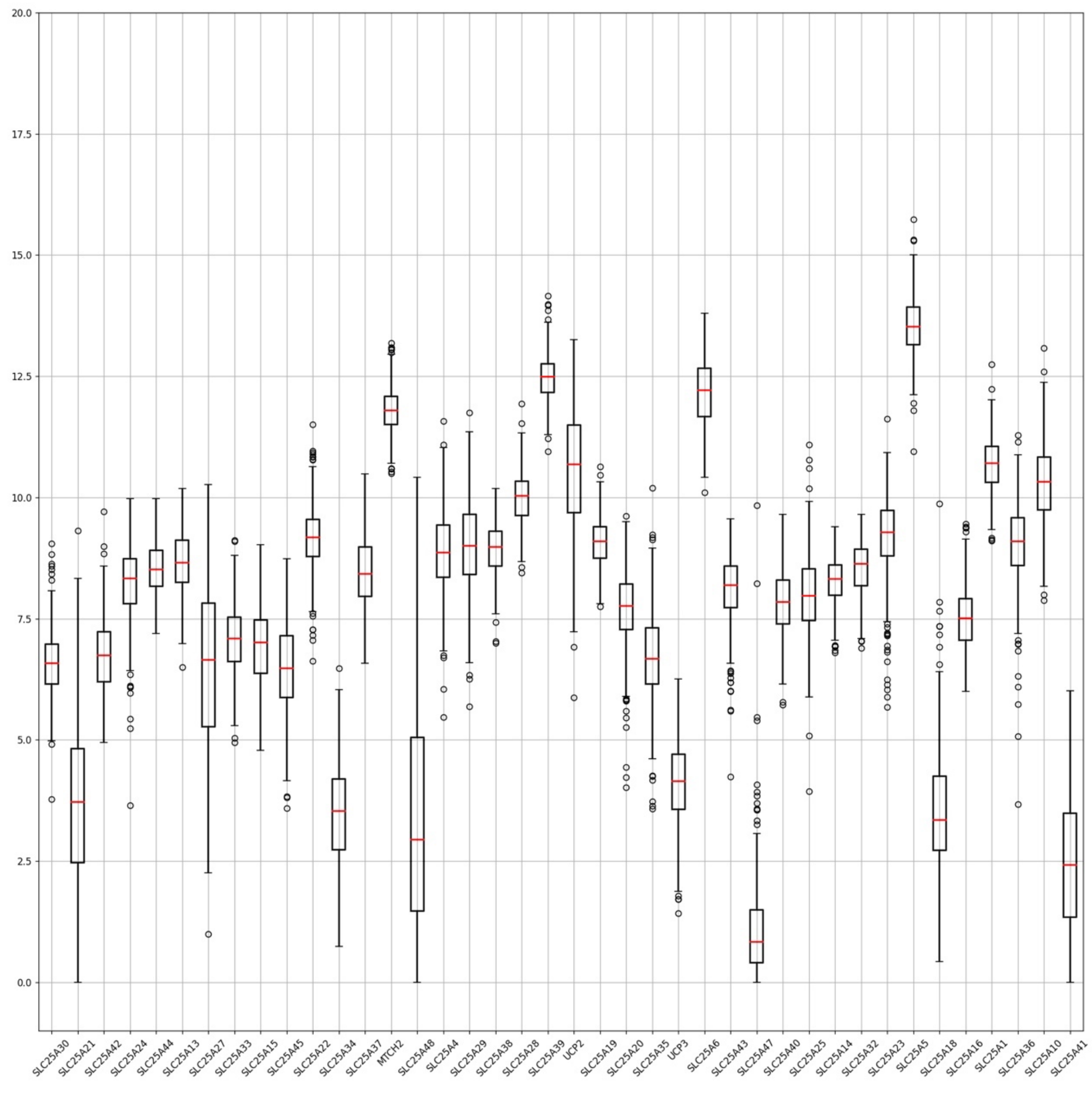

Figure S9AC.

Boxplot of SLC25 transporter expression cluster 32 of TCGA tumors (log_2FPKM)

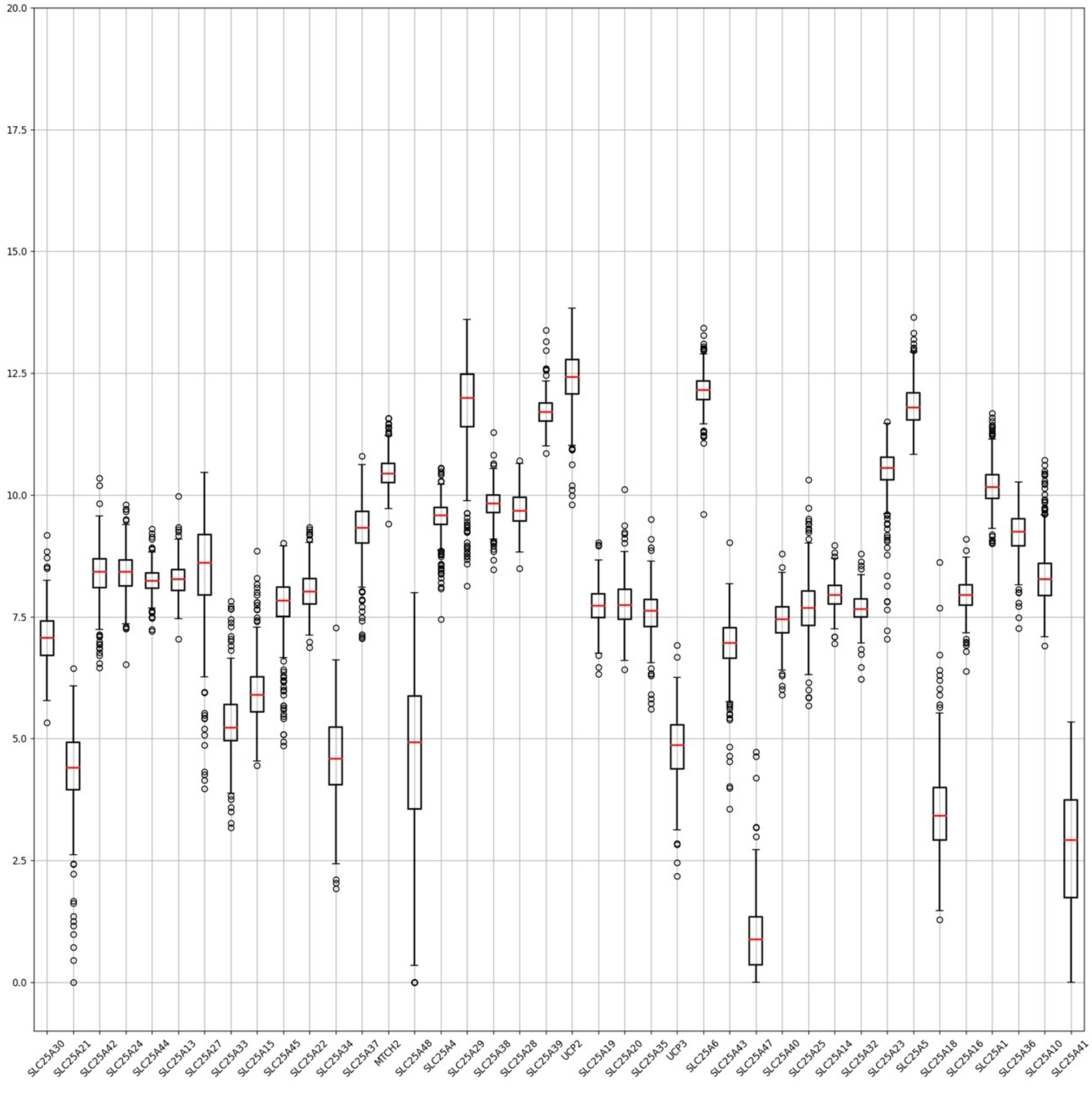

Figure S9AD.

Boxplot of SLC25 transporter expression cluster 33 of TCGA tumors (log_2FPKM)

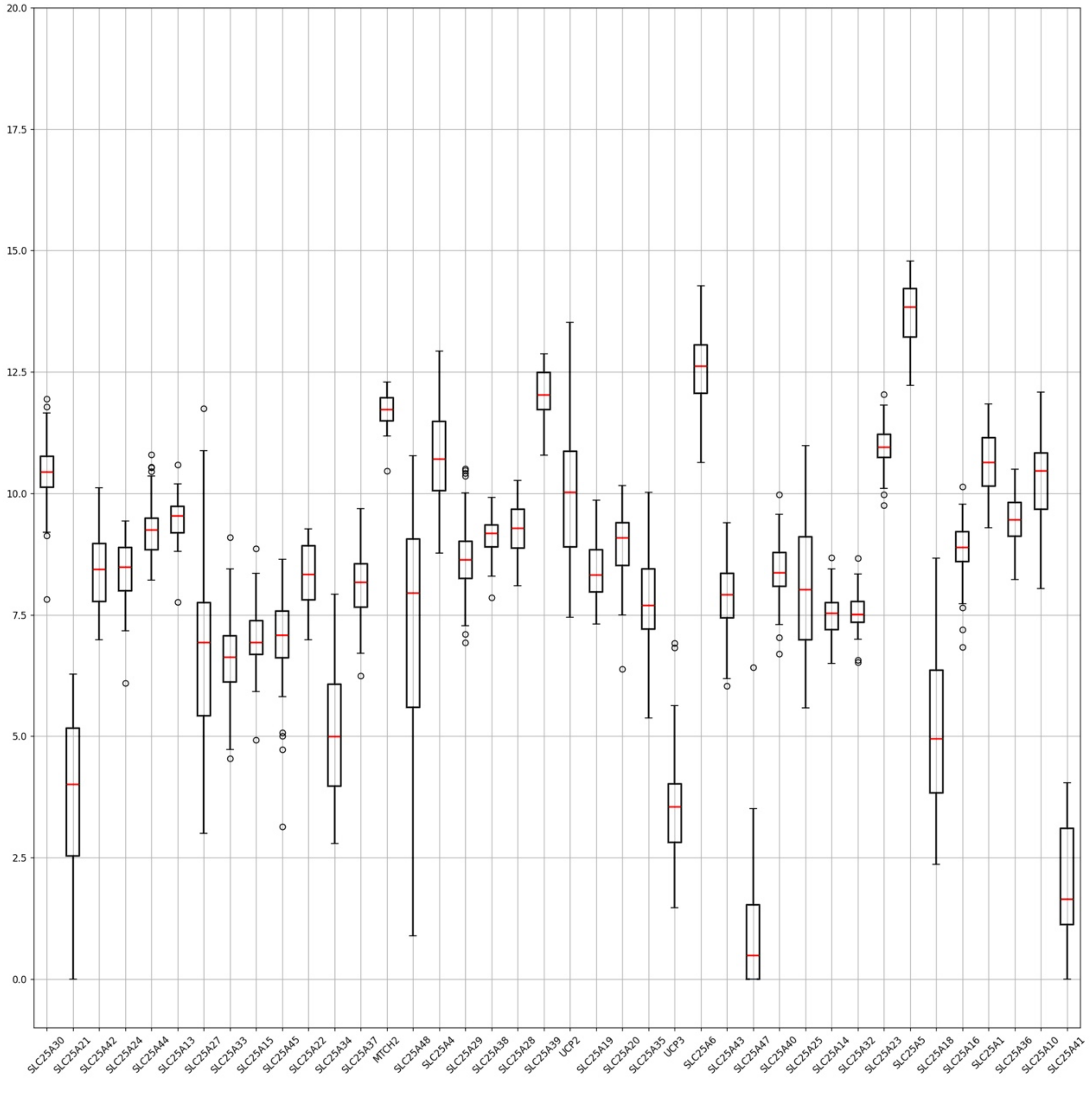

Figure S9AE.

Boxplot of SLC25 transporter expression cluster 34 of TCGA tumors (log_2FPKM)

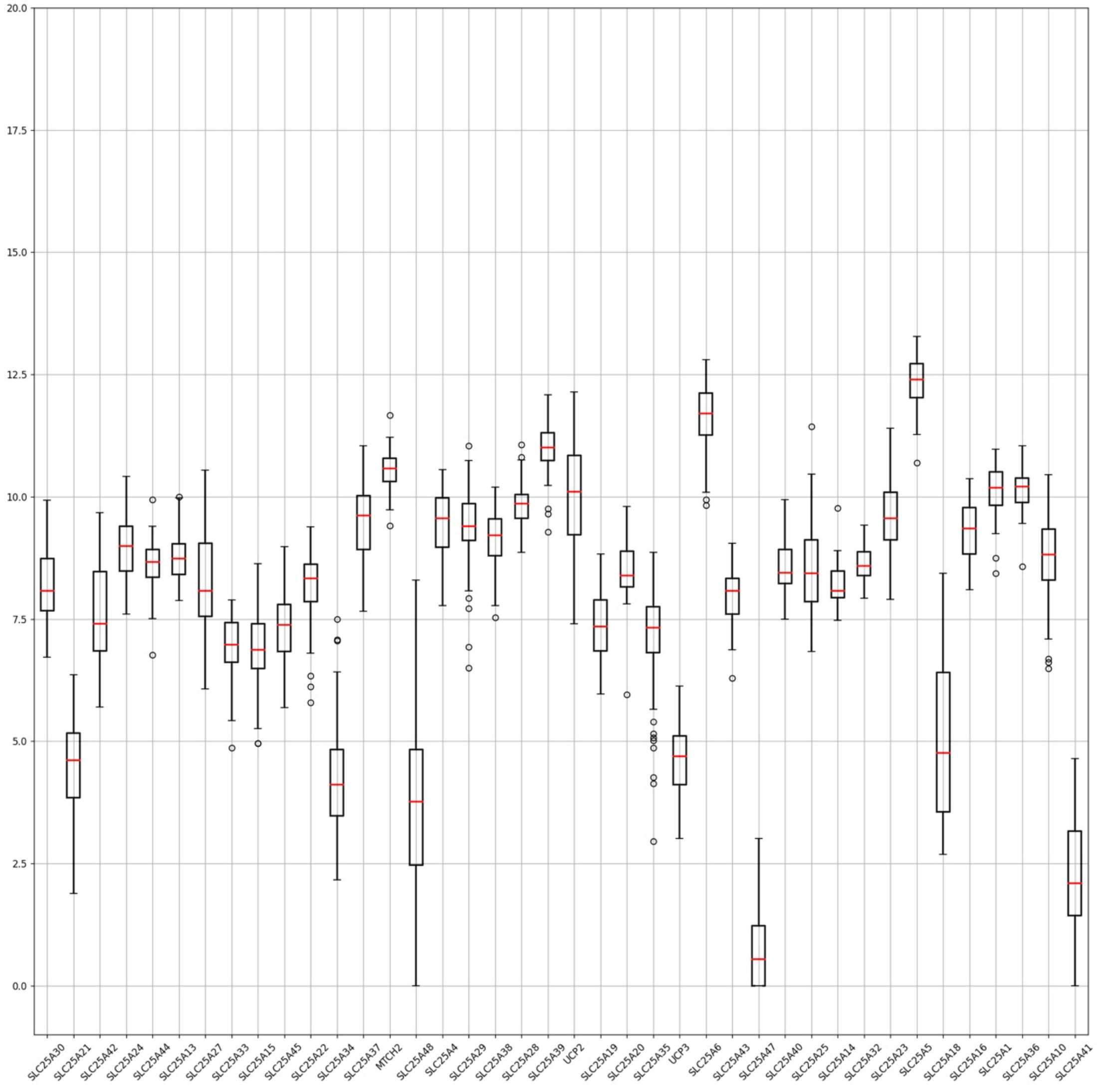

Figure S9AF.

Boxplot of SLC25 transporter expression cluster 36 of TCGA tumors (log_2FPKM)

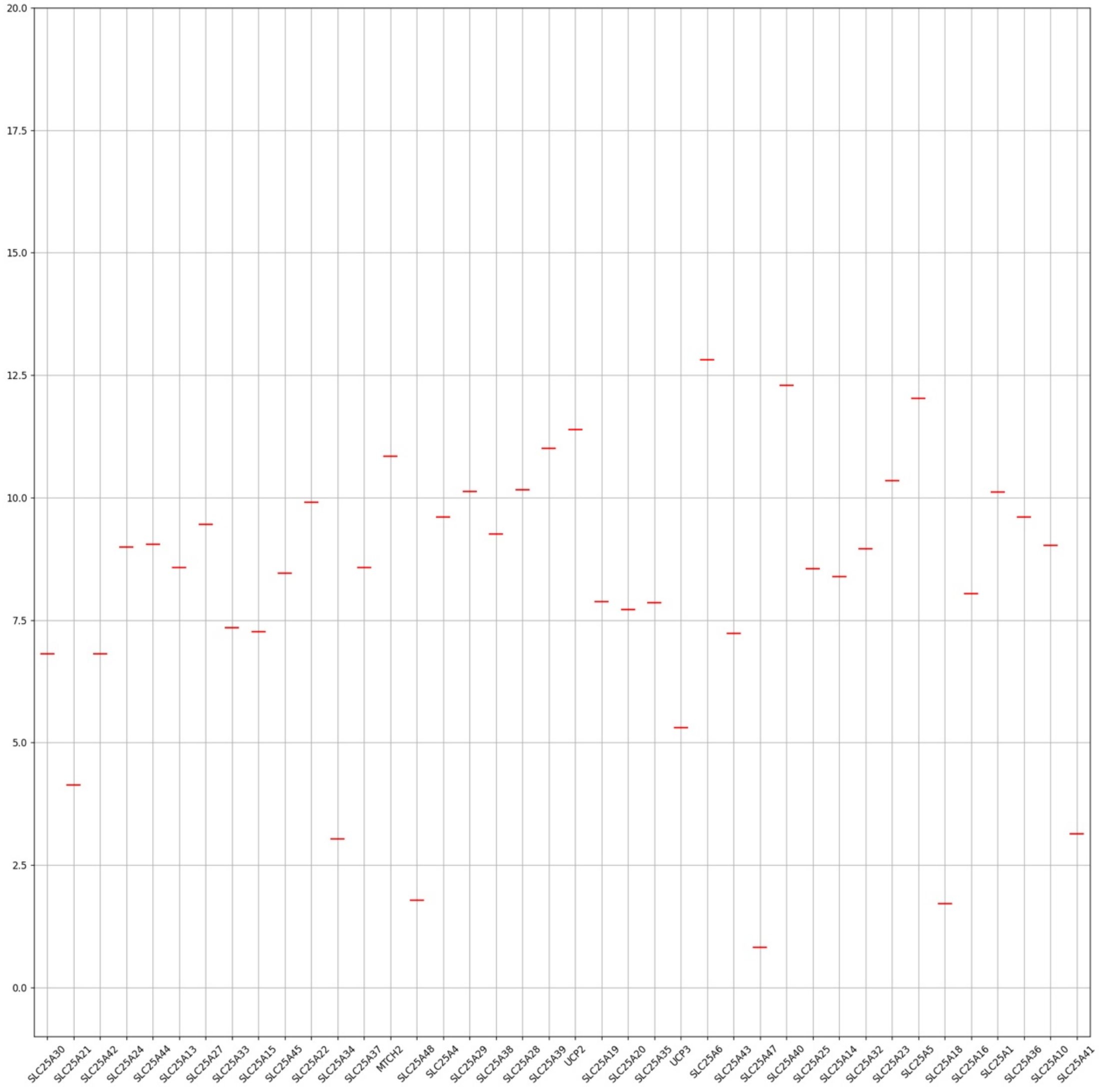

Figure S9AG.

Boxplot of SLC25 transporter expression cluster 37 of TCGA tumors (log_2FPKM)

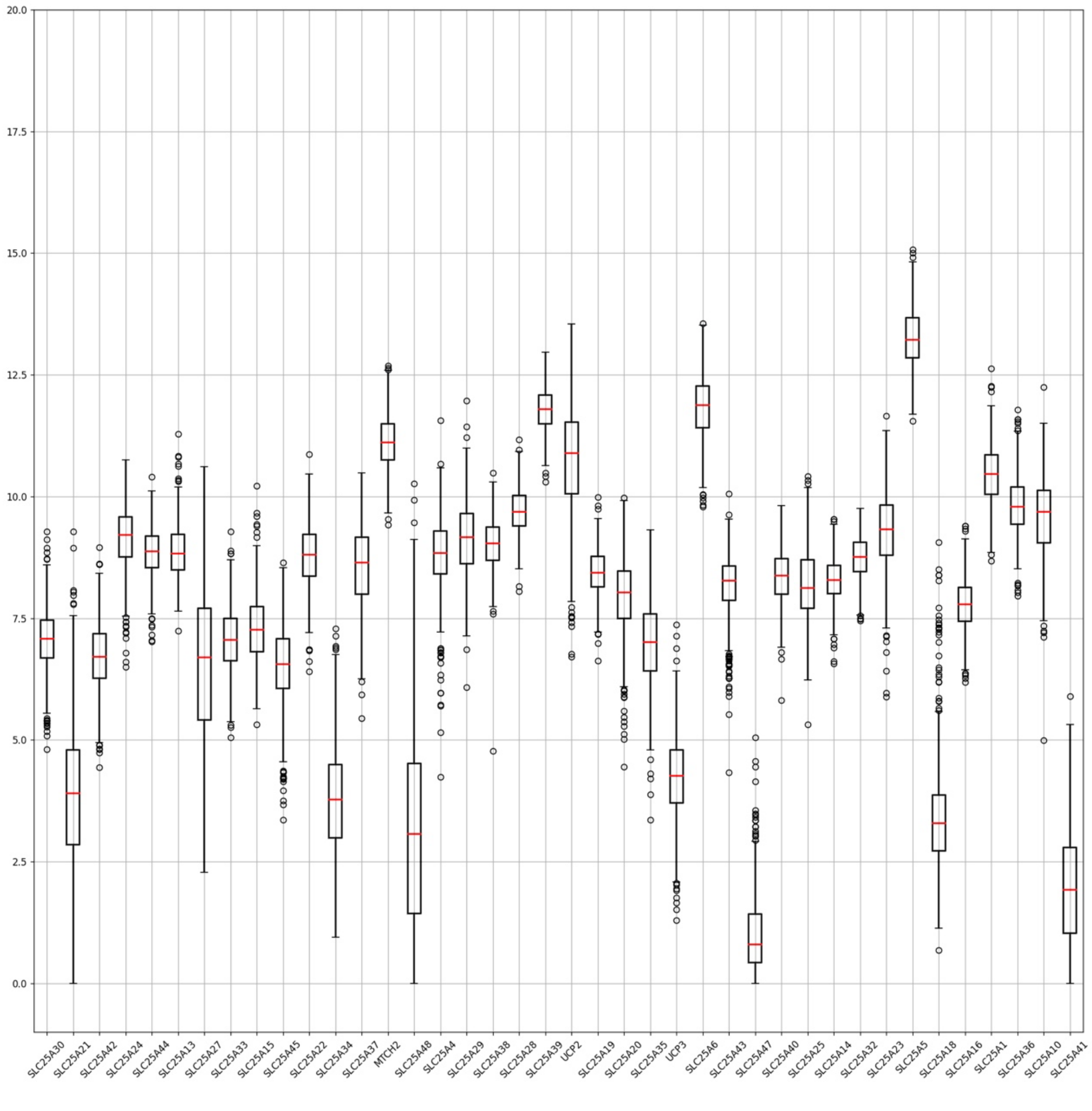

Figure S9AH.

Boxplot of SLC25 transporter expression cluster 38 of TCGA tumors (log_2FPKM)

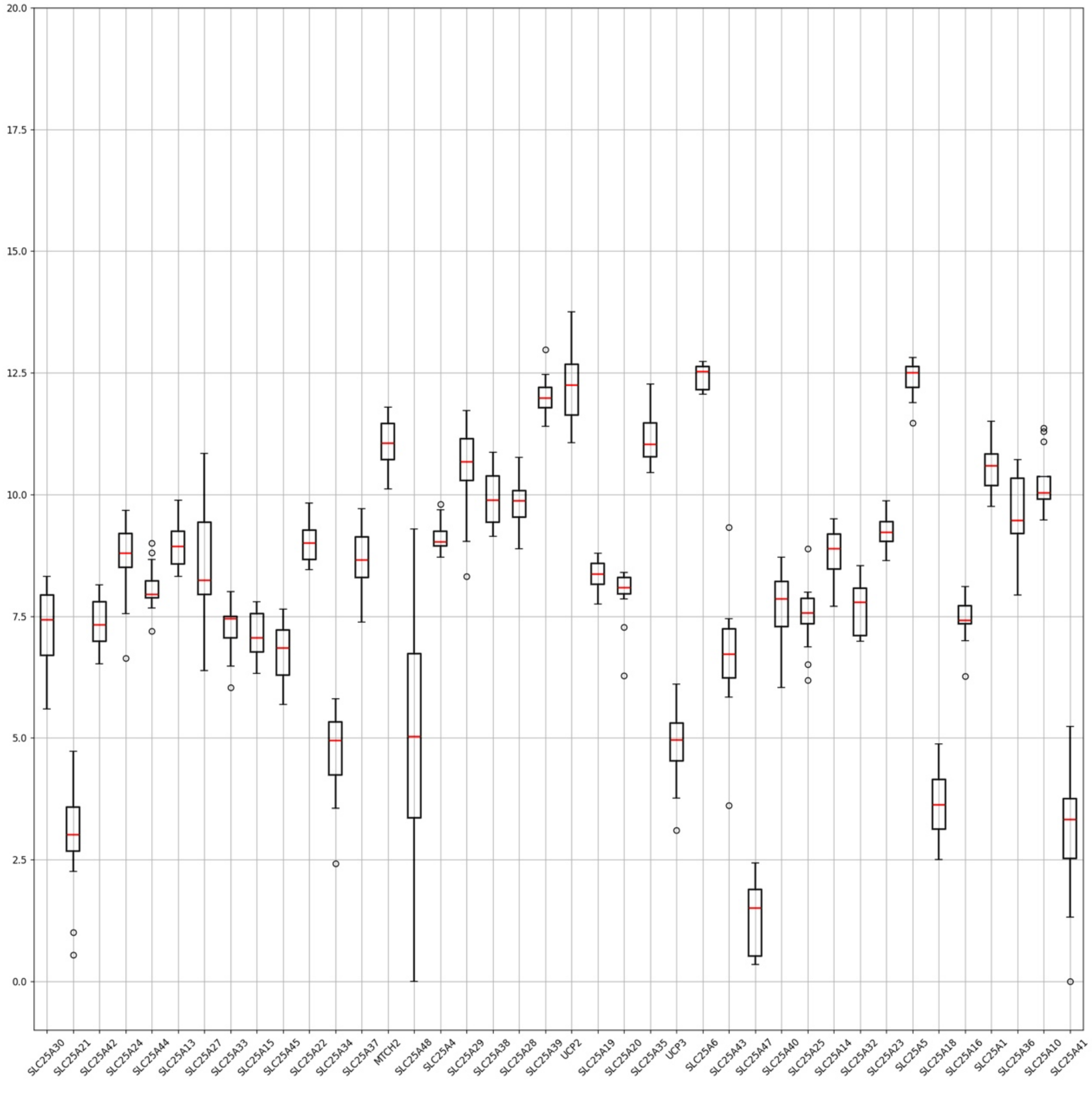

Figure S9AI.

Boxplot of SLC25 transporter expression cluster 39 of TCGA tumors (log_2FPKM)

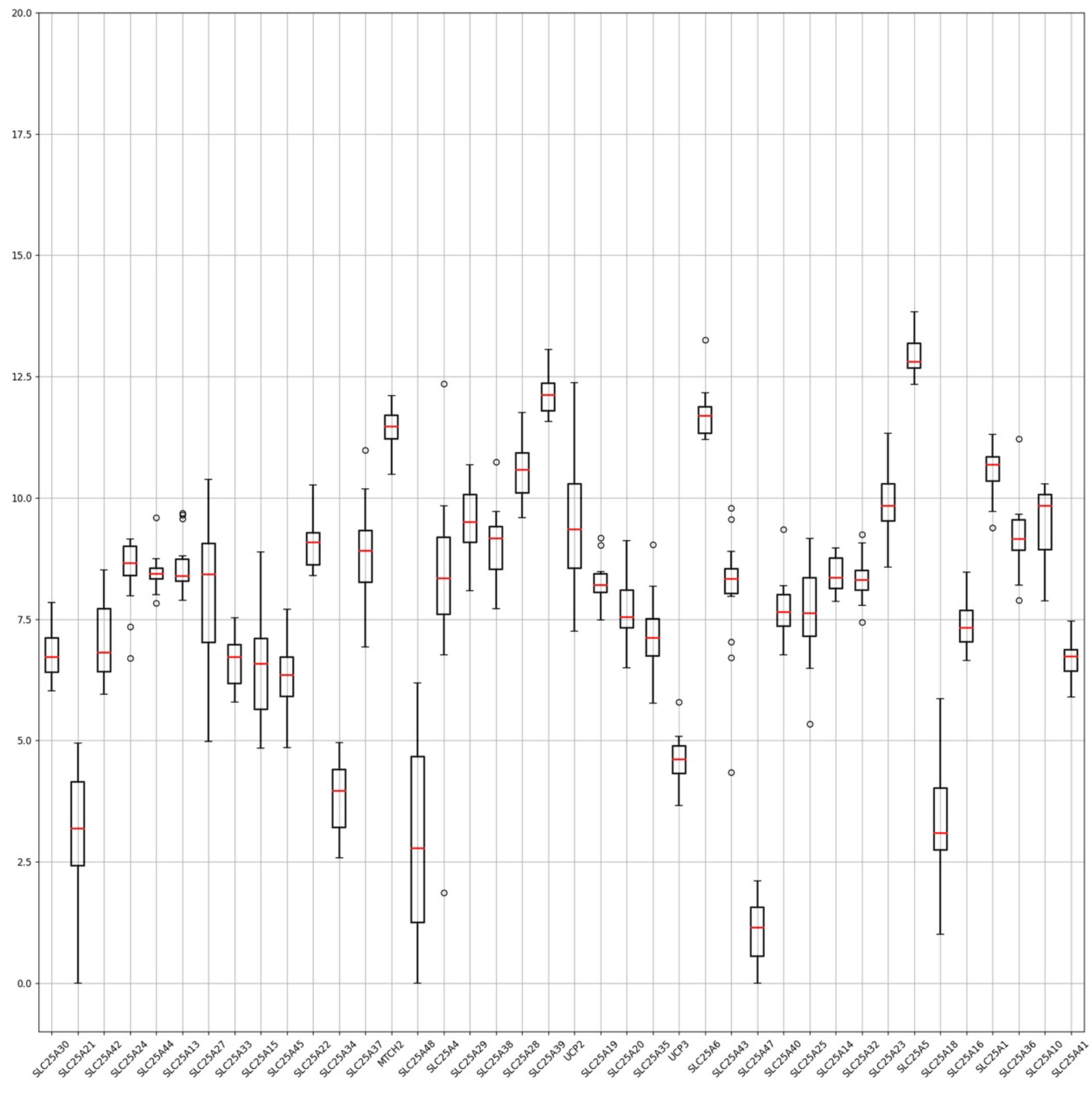

Figure S9AJ.

Boxplot of SLC25 transporter expression cluster 40 of TCGA tumors (log_2FPKM)

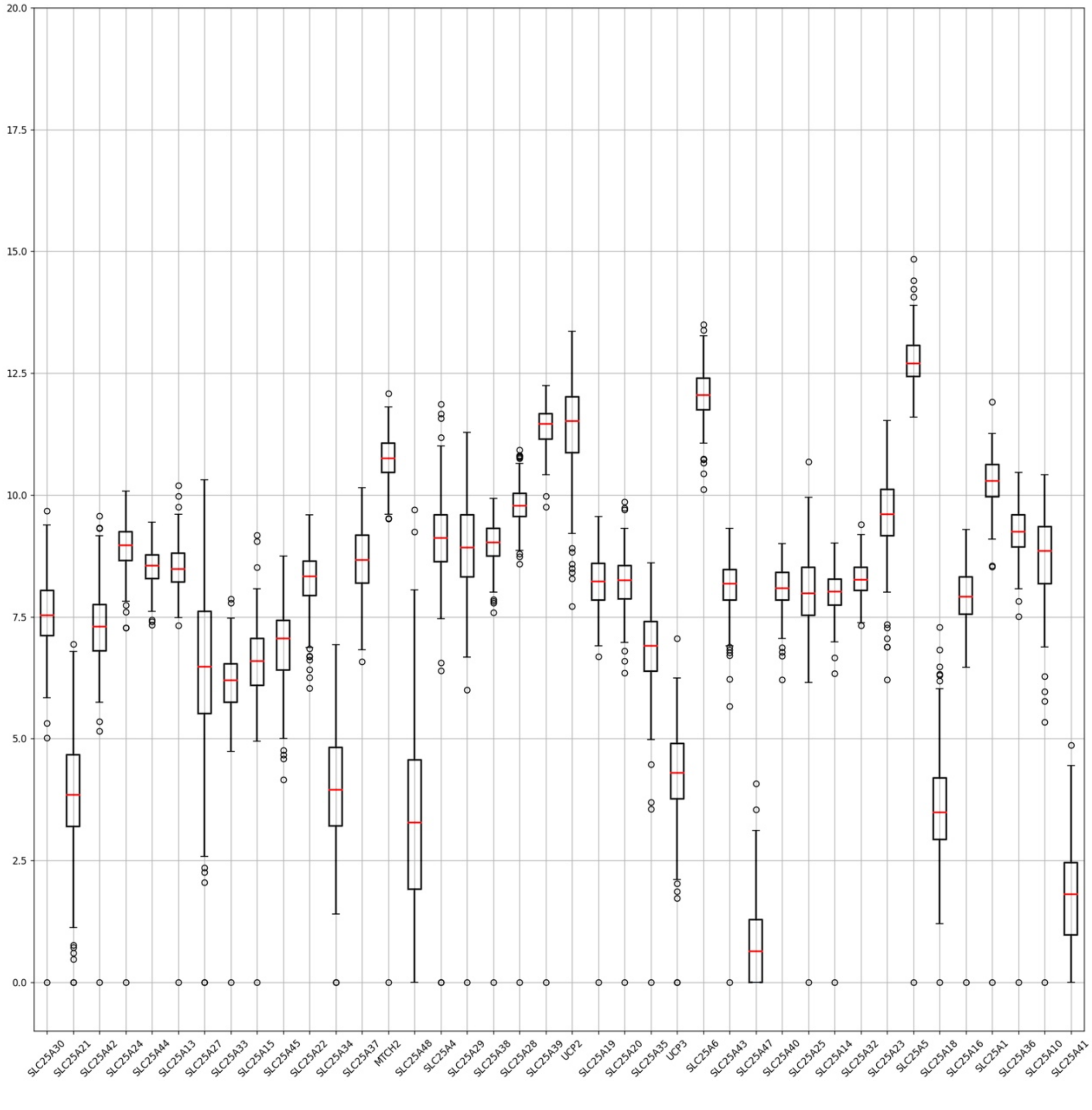

Figure S9AK.

Boxplot of SLC25 transporter expression cluster 41 of TCGA tumors (log_2FPKM)

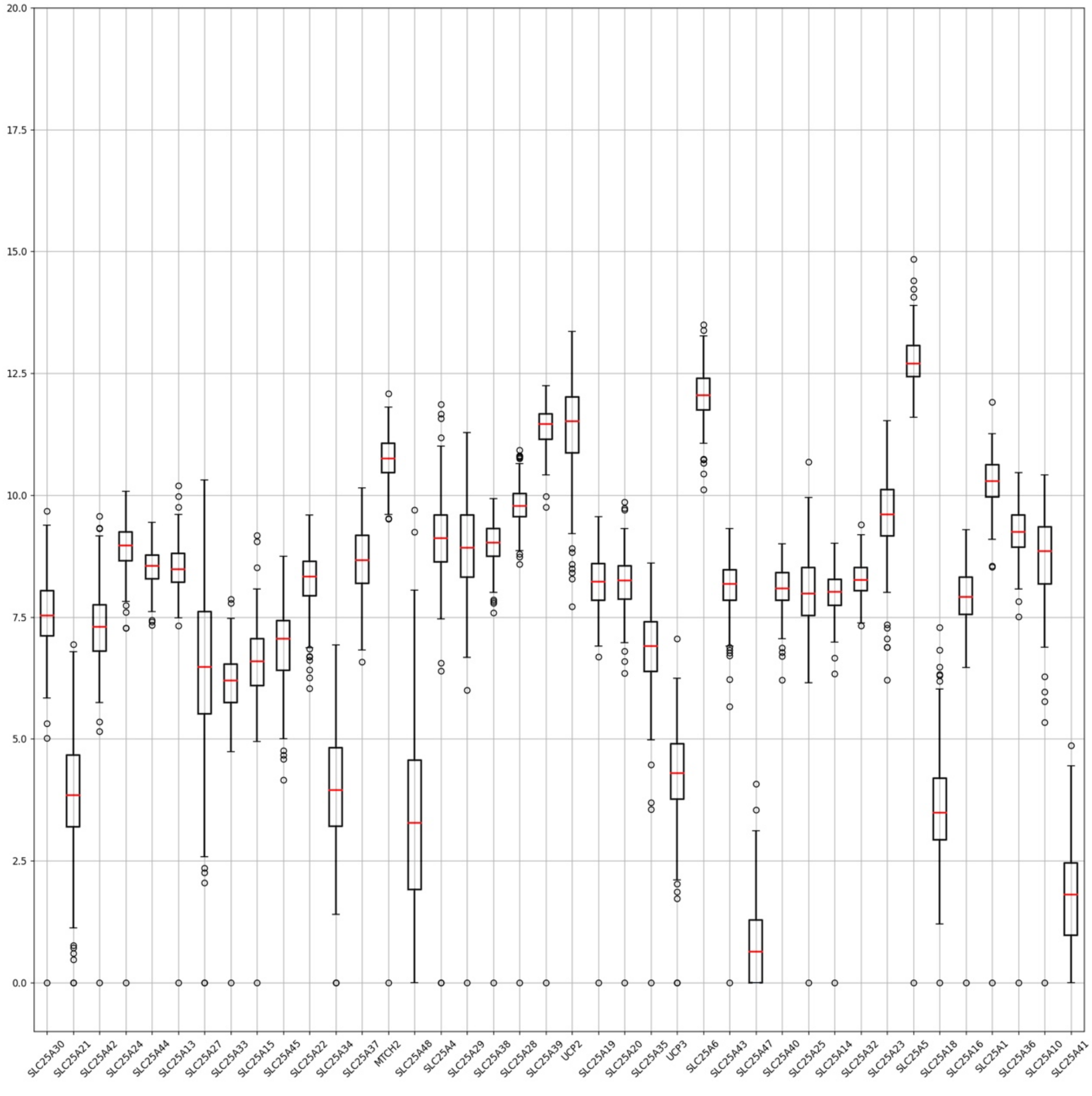

Figure S9AL.

Boxplot of SLC25 transporter expression cluster 42 of TCGA tumors (log_2FPKM)

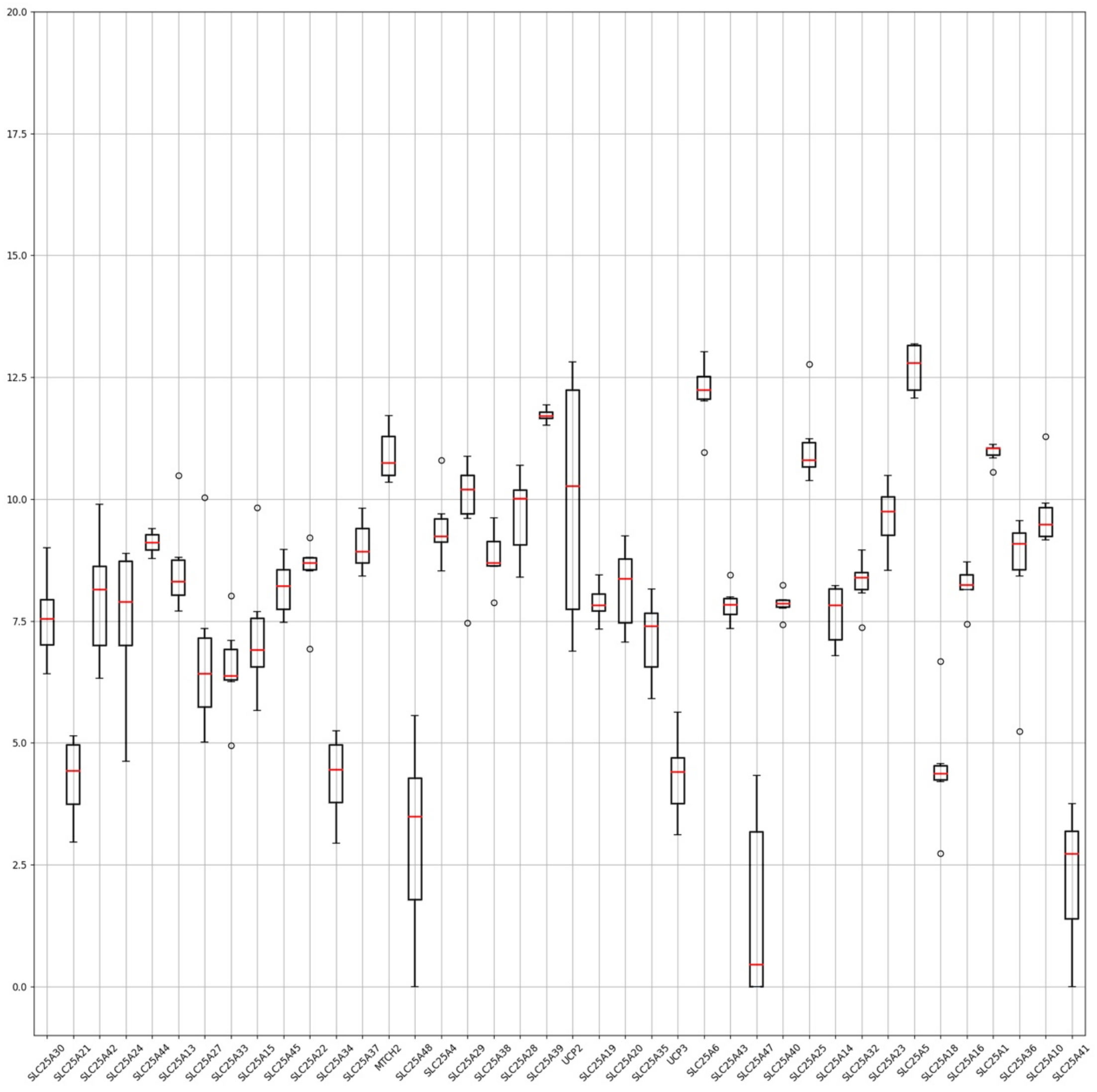

Figure S9AM.

Boxplot of SLC25 transporter expression cluster 43 of TCGA tumors (log_2FPKM)

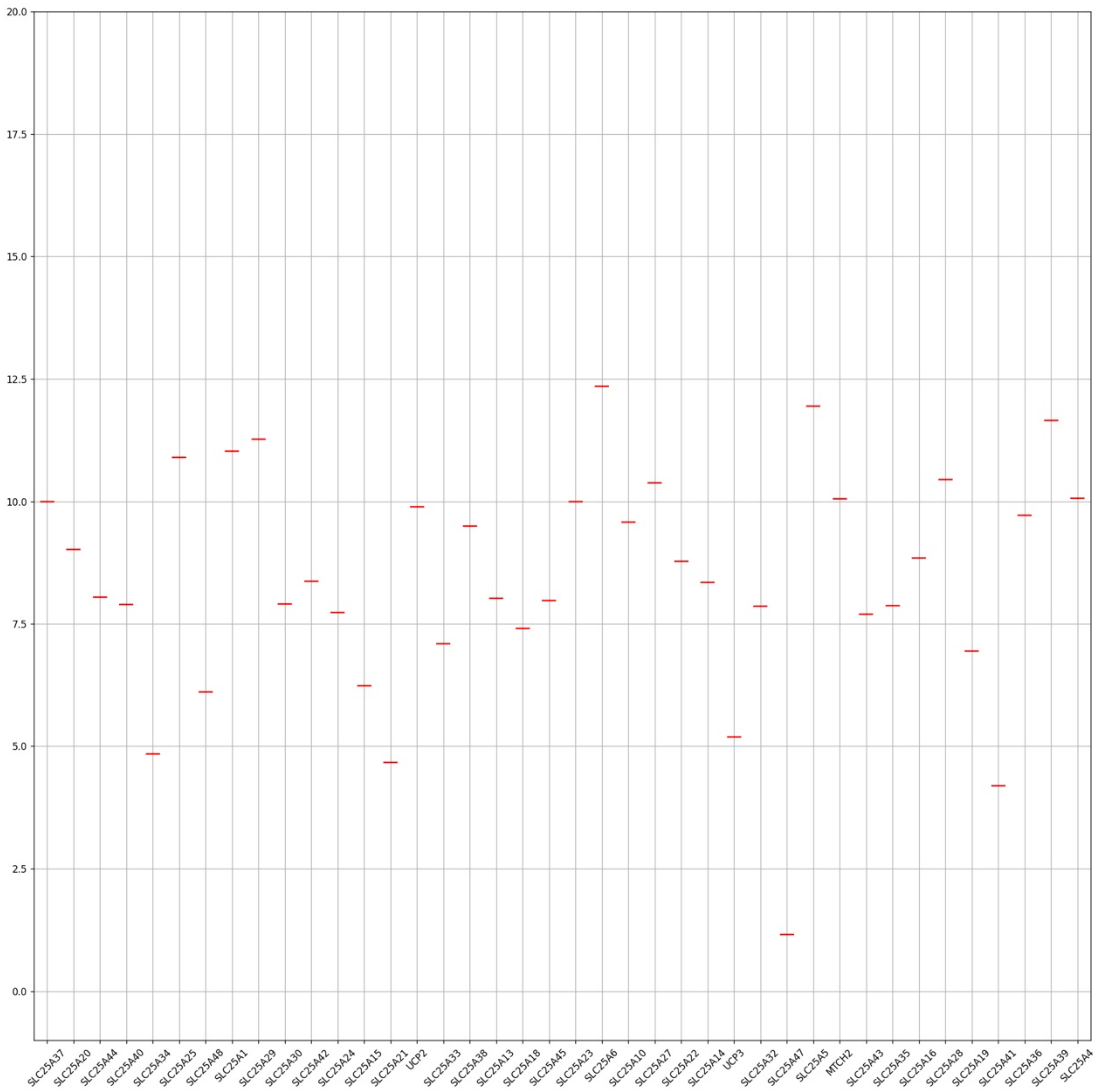

Figure S10A.

Boxplot of SLC25 transporter expression cluster 0 of GTEx normal tissues (log_2FPKM)

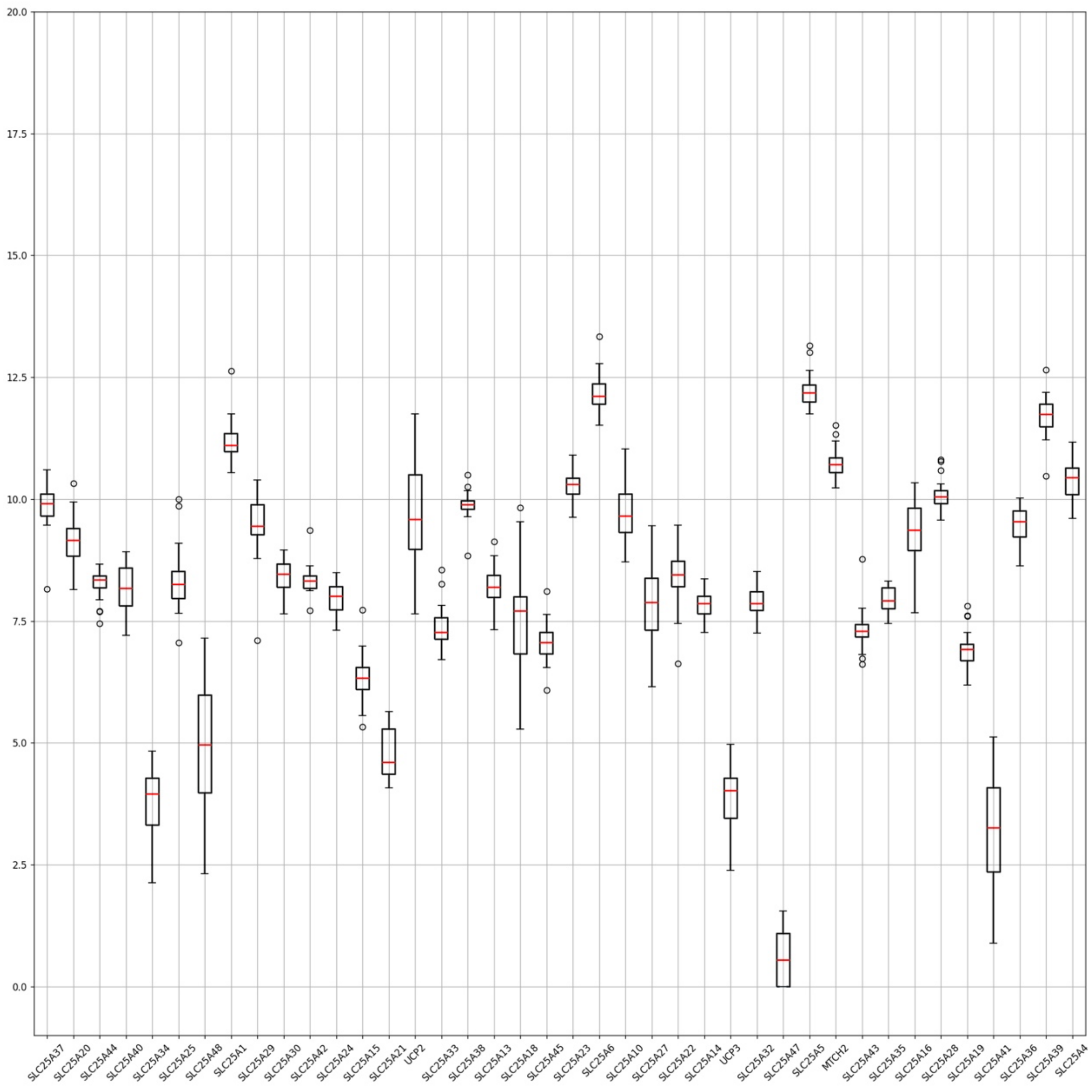

Figure S10B.

Boxplot of SLC25 transporter expression cluster 1 of GTEx normal tissues (log_2FPKM)

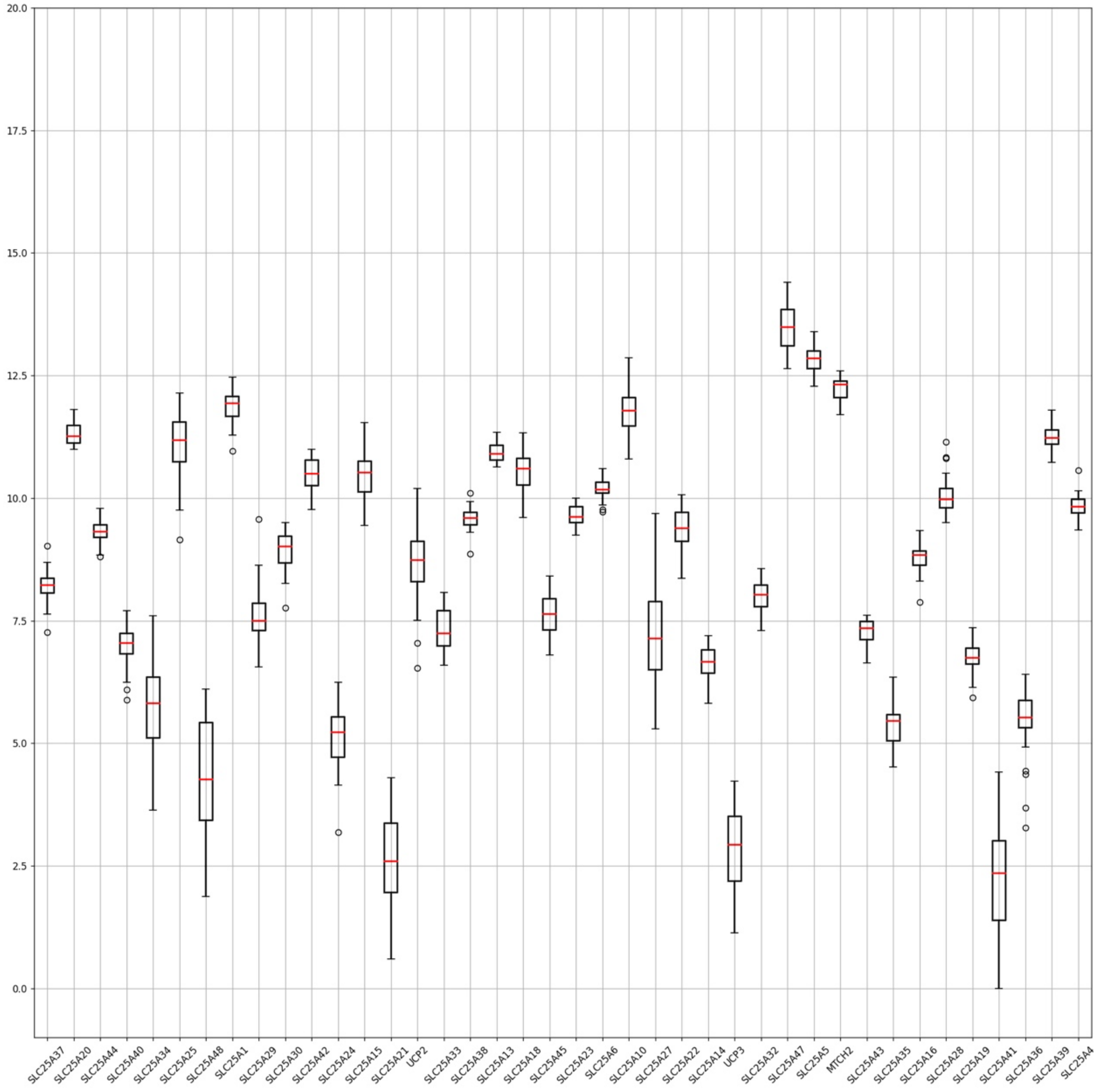

Figure S10C.

Boxplot of SLC25 transporter expression cluster 2 of GTEx normal tissues (log_2FPKM)

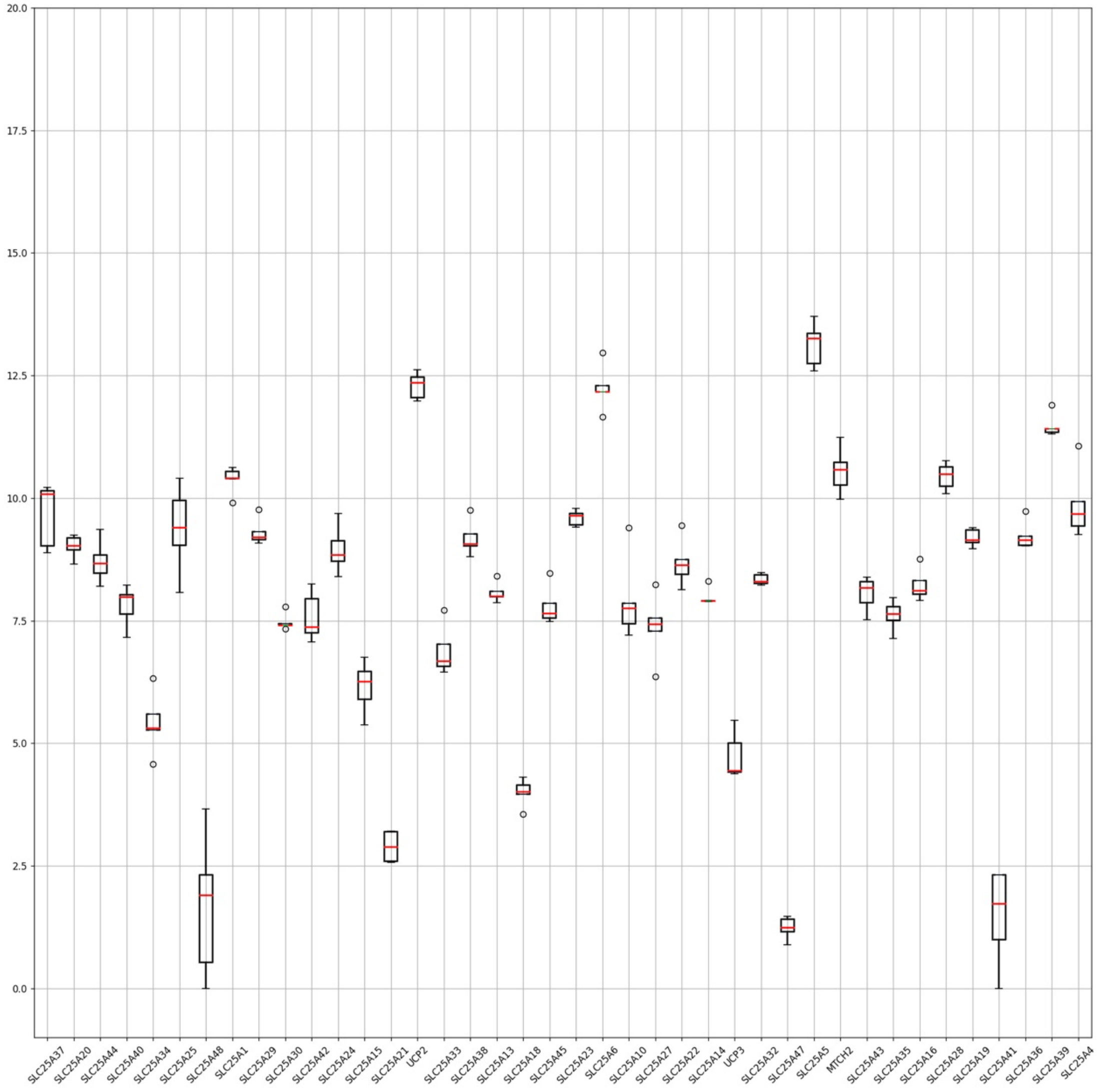

Figure S10D.

Boxplot of SLC25 transporter expression cluster 3 of GTEx normal tissues (log_2FPKM)

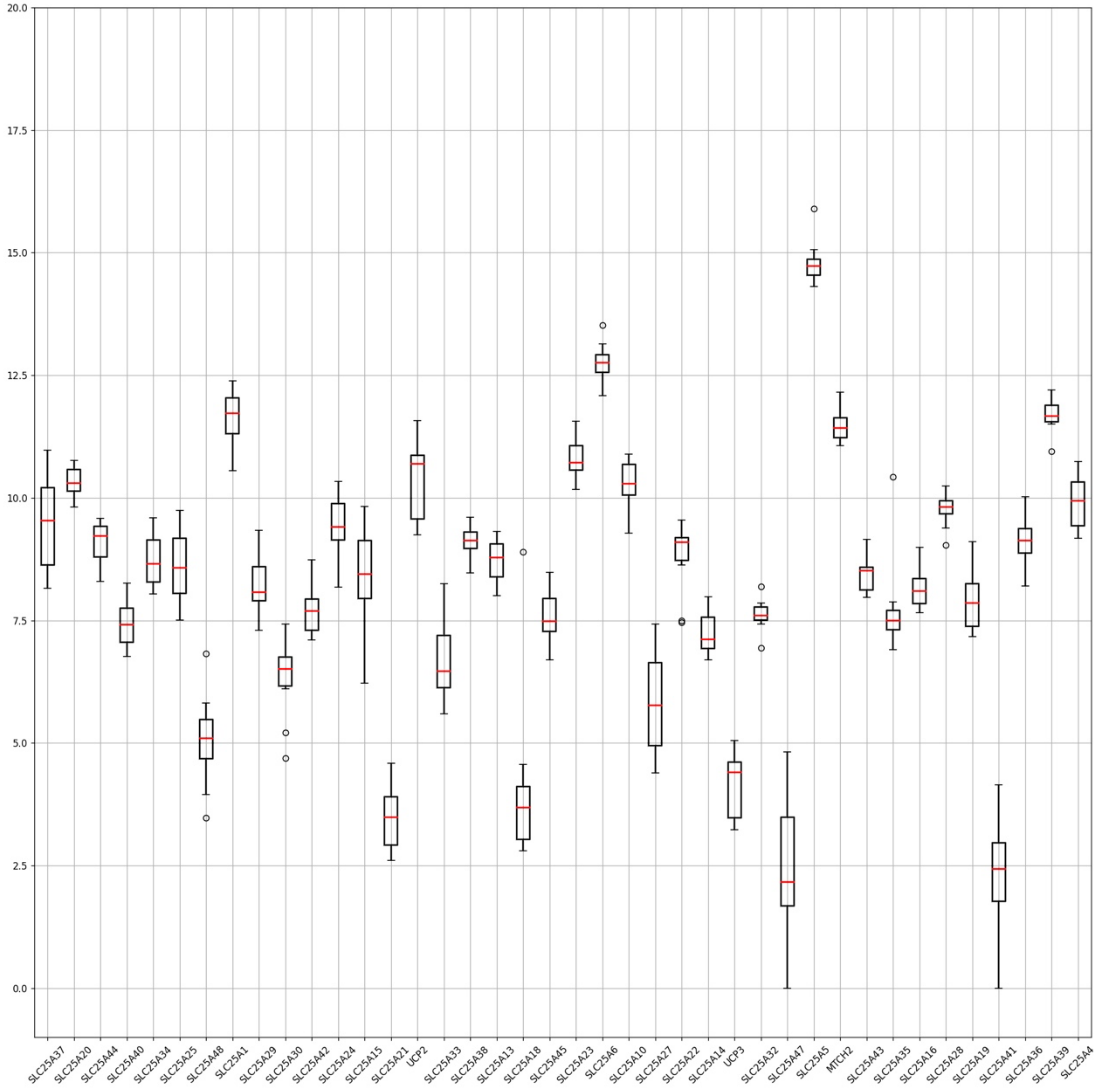

Figure S10E.

Boxplot of SLC25 transporter expression cluster 4 of GTEx normal tissues (log_2FPKM)

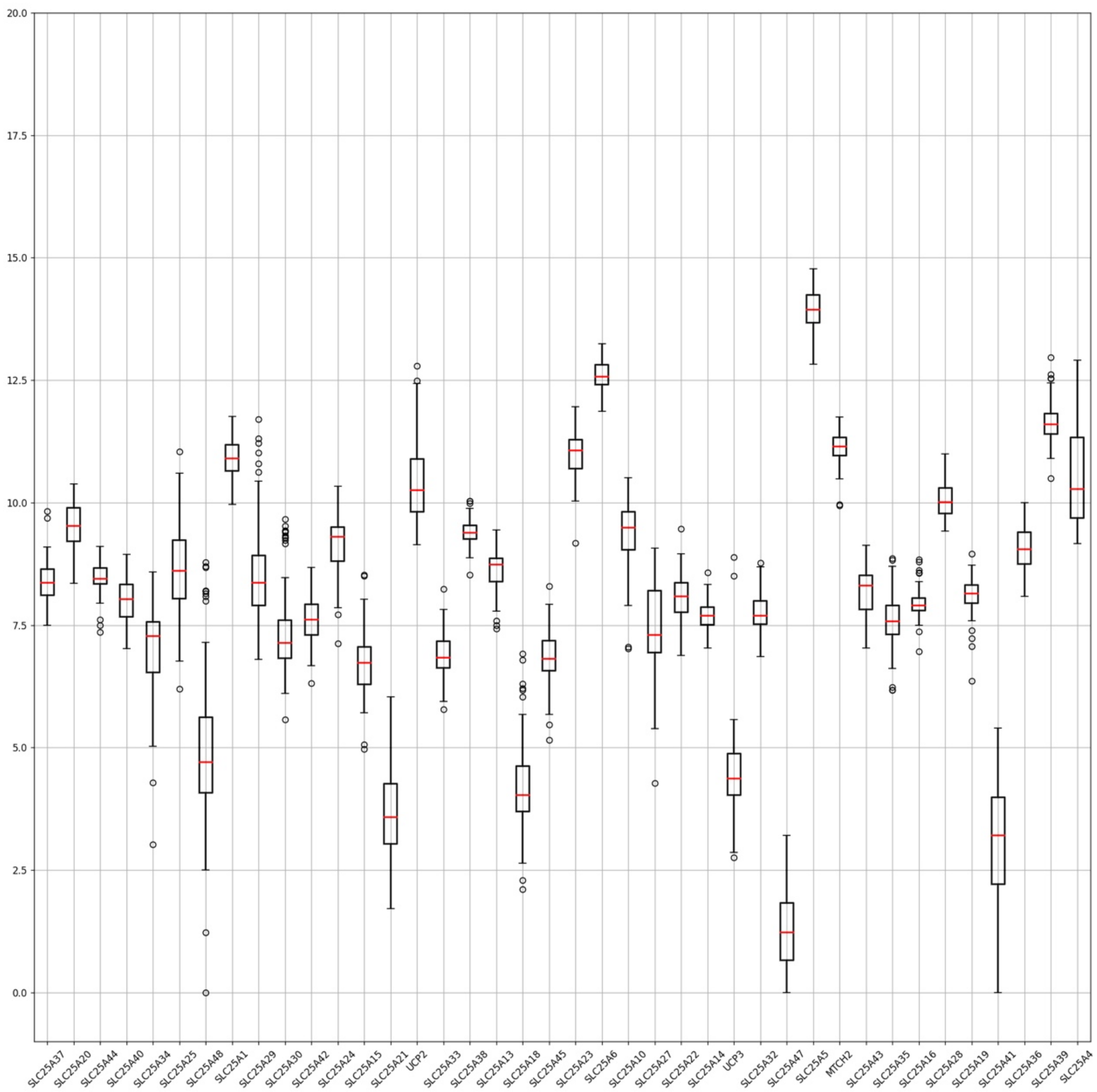

Figure S10F.

Boxplot of SLC25 transporter expression cluster 5 of GTEx normal tissues (log_2FPKM)

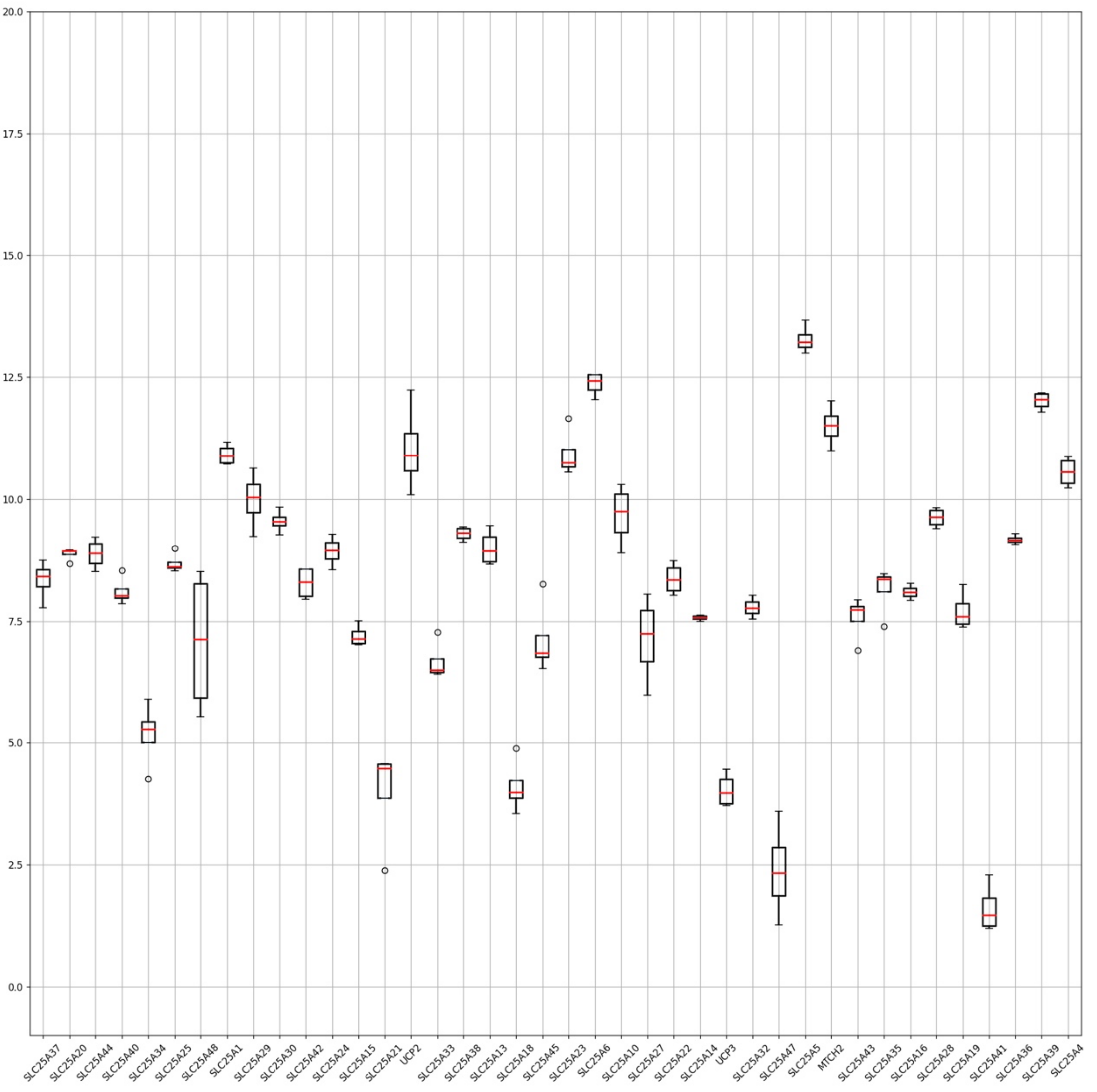

Figure S10G.

Boxplot of SLC25 transporter expression cluster 6 of GTEx normal tissues (log_2FPKM)

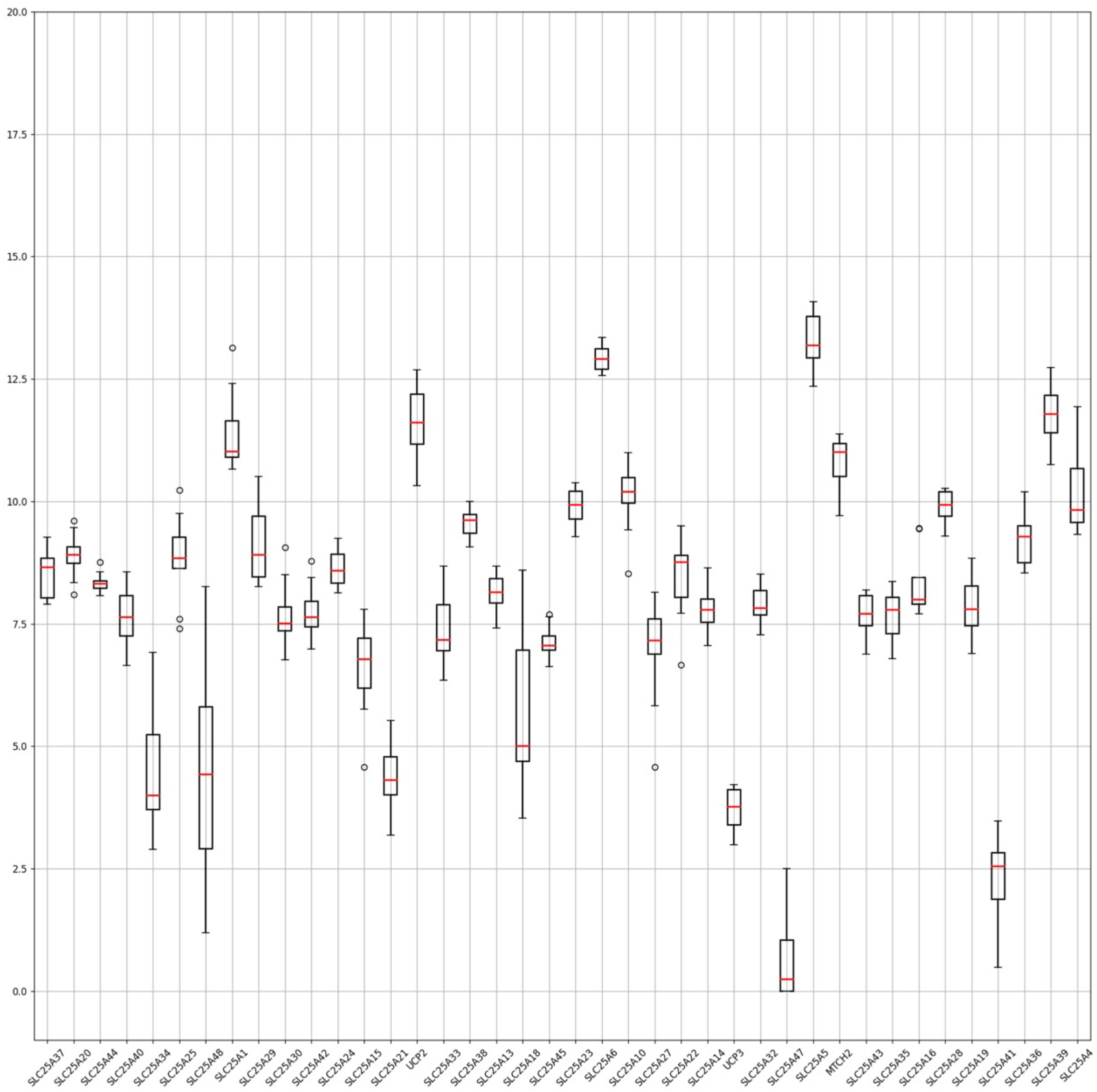

Figure S10H.

Boxplot of SLC25 transporter expression cluster 7 of GTEx normal tissues (log_2FPKM)

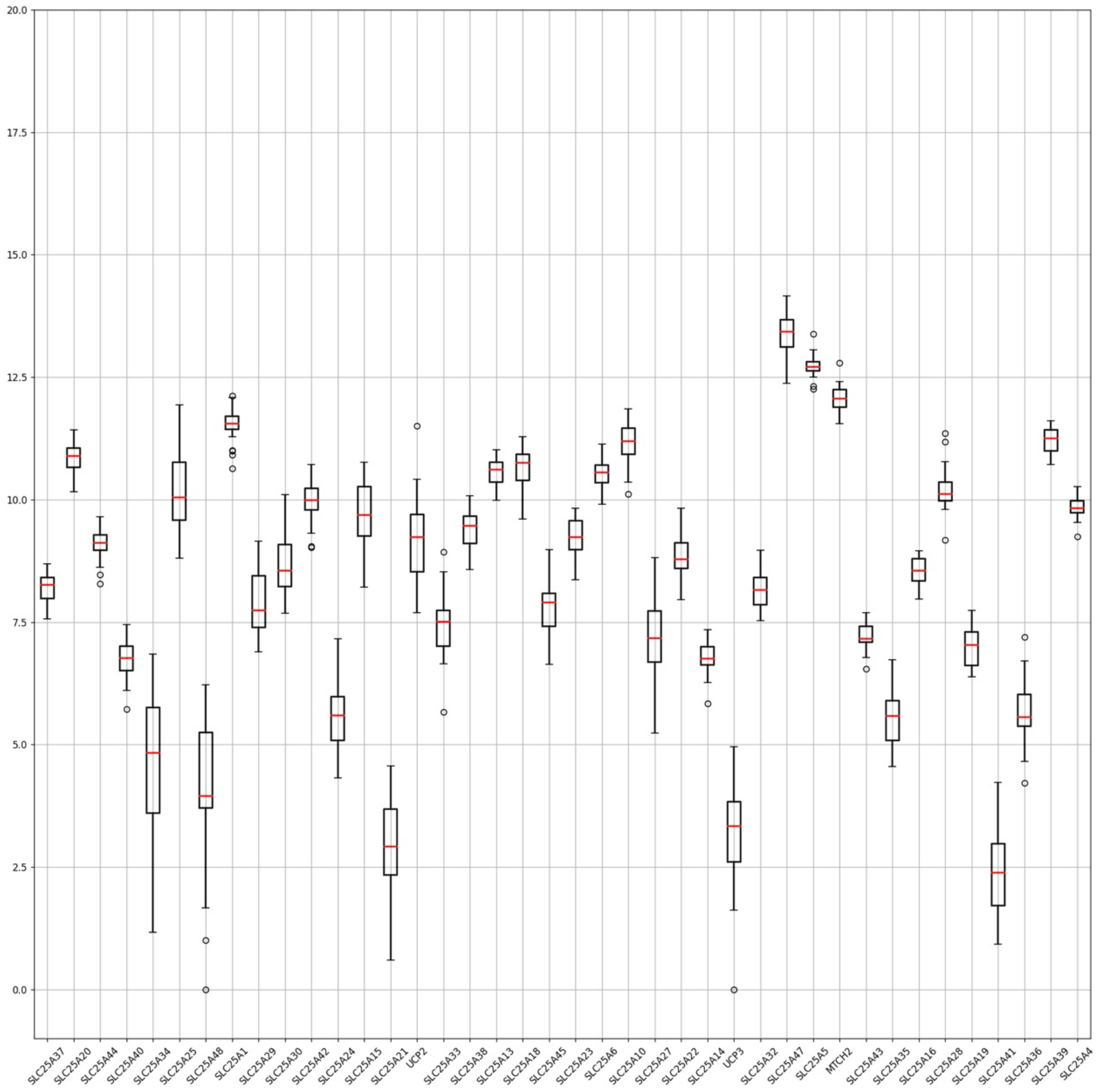

Figure S10I.

Boxplot of SLC25 transporter expression cluster 12 of GTEx normal tissues (log_2FPKM)

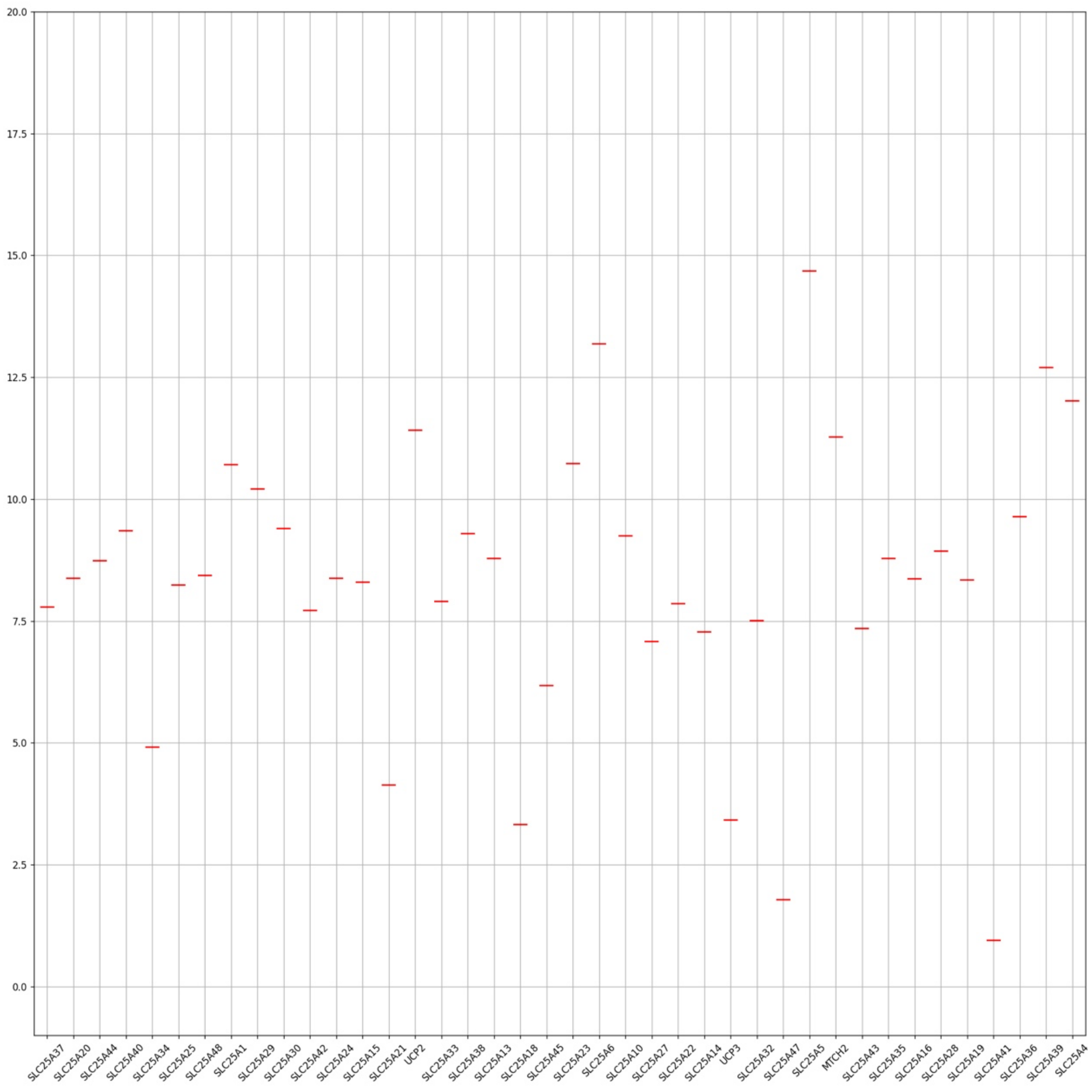

Figure S10J.

Boxplot of SLC25 transporter expression cluster 13 of GTEx normal tissues (log_2FPKM)

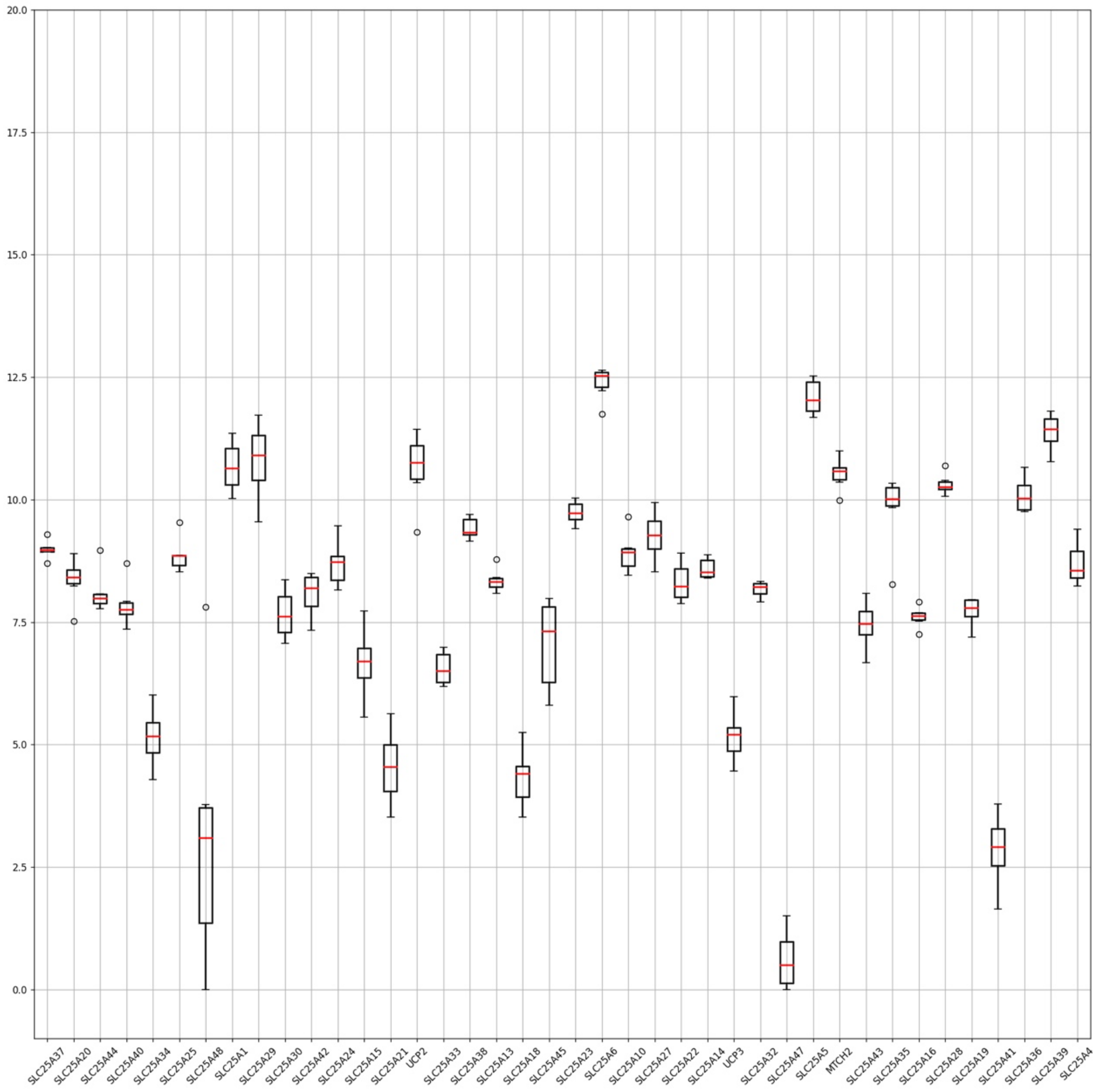

Figure S10K.

Boxplot of SLC25 transporter expression cluster 14 of GTEx normal tissues (log_2FPKM)

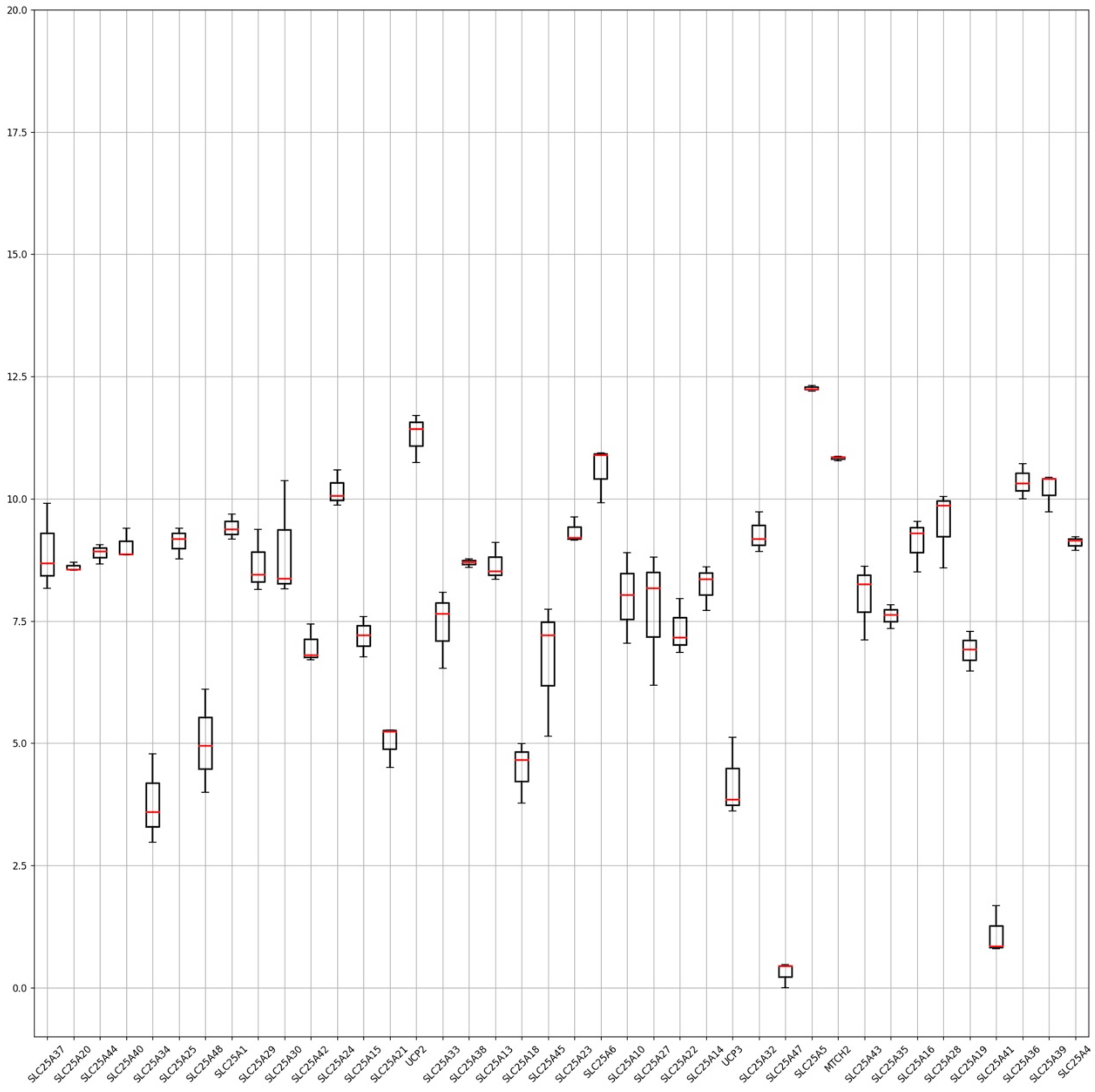

Figure S10L.

Boxplot of SLC25 transporter expression cluster 16 of GTEx normal tissues (log_2FPKM)

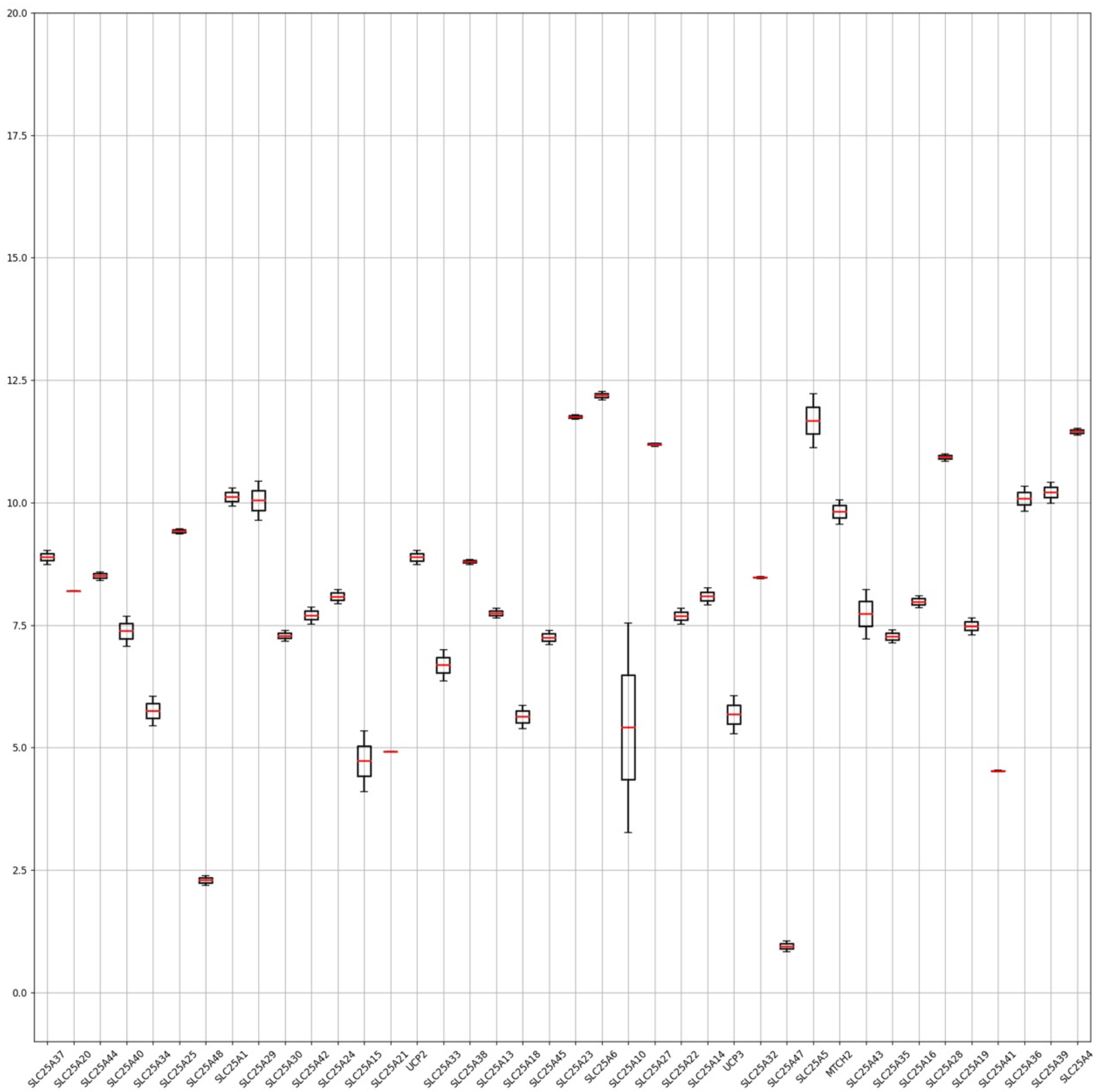

Figure S10M.

Boxplot of SLC25 transporter expression cluster 19 of GTEx normal tissues (log_2FPKM)

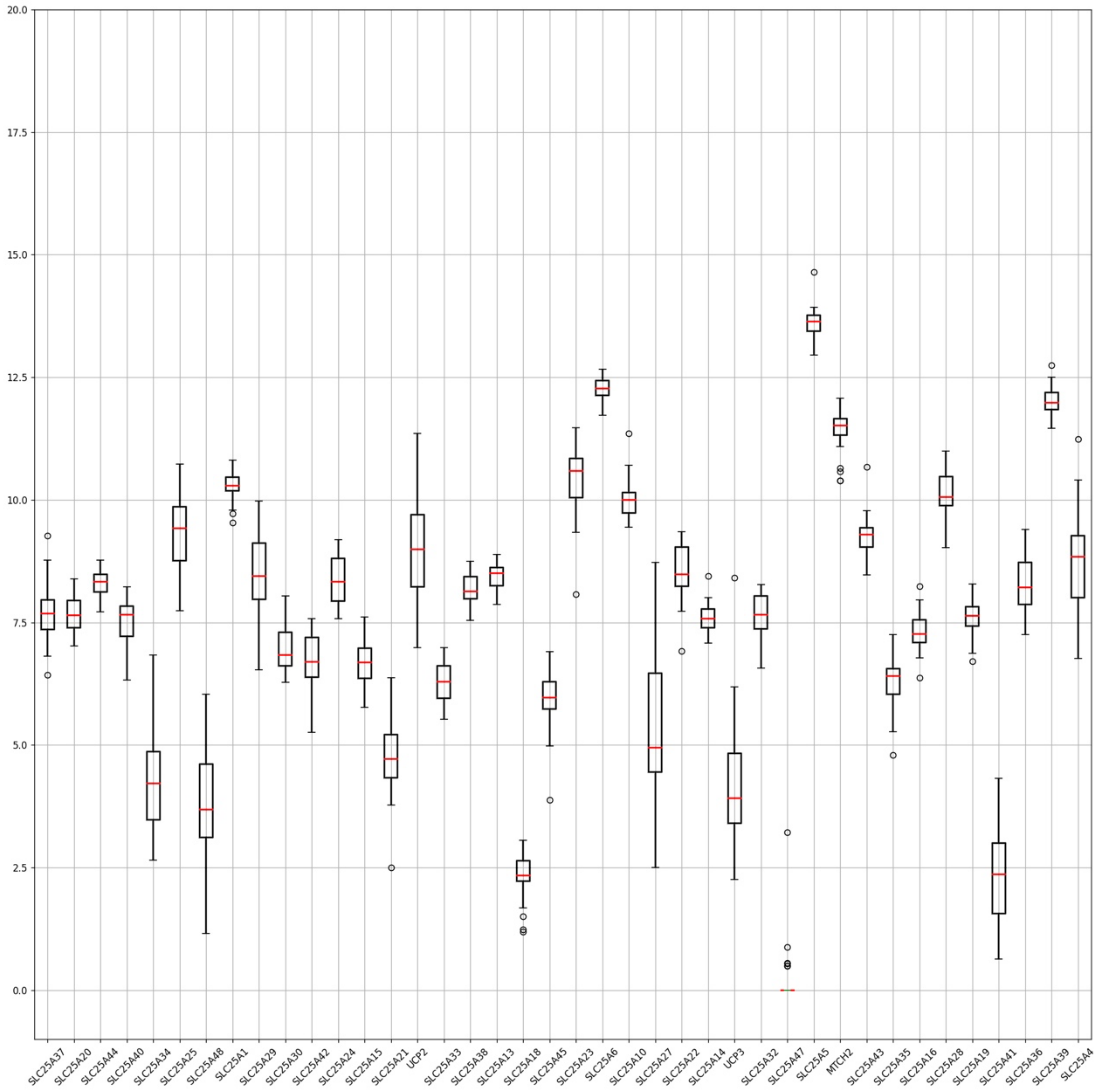

Figure S10N.

Boxplot of SLC25 transporter expression cluster 20 of GTEx normal tissues ($\log_2$FPKM)

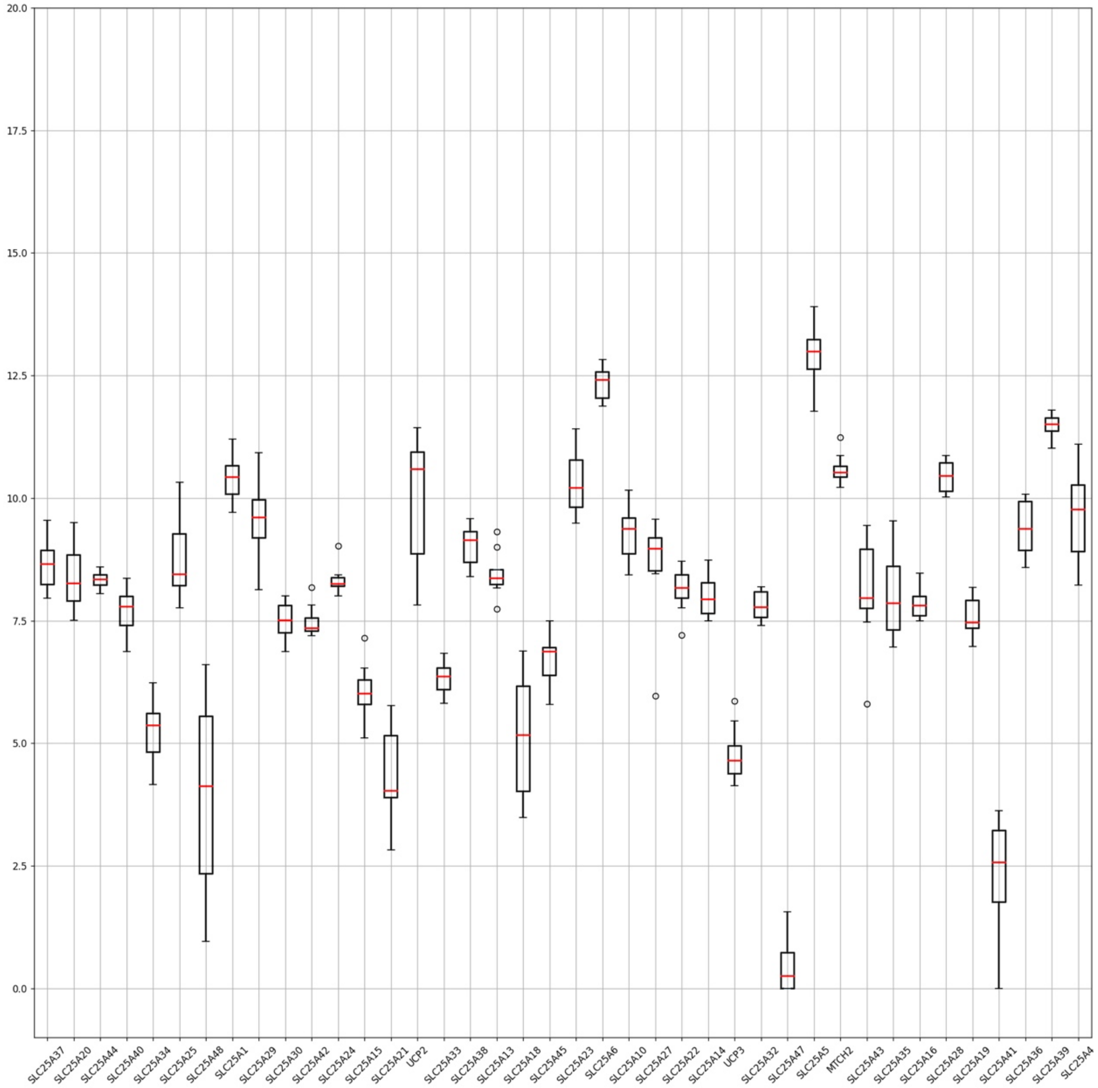

Figure S10O.

Boxplot of SLC25 transporter expression cluster 21 of GTEx normal tissues (log_2FPKM)

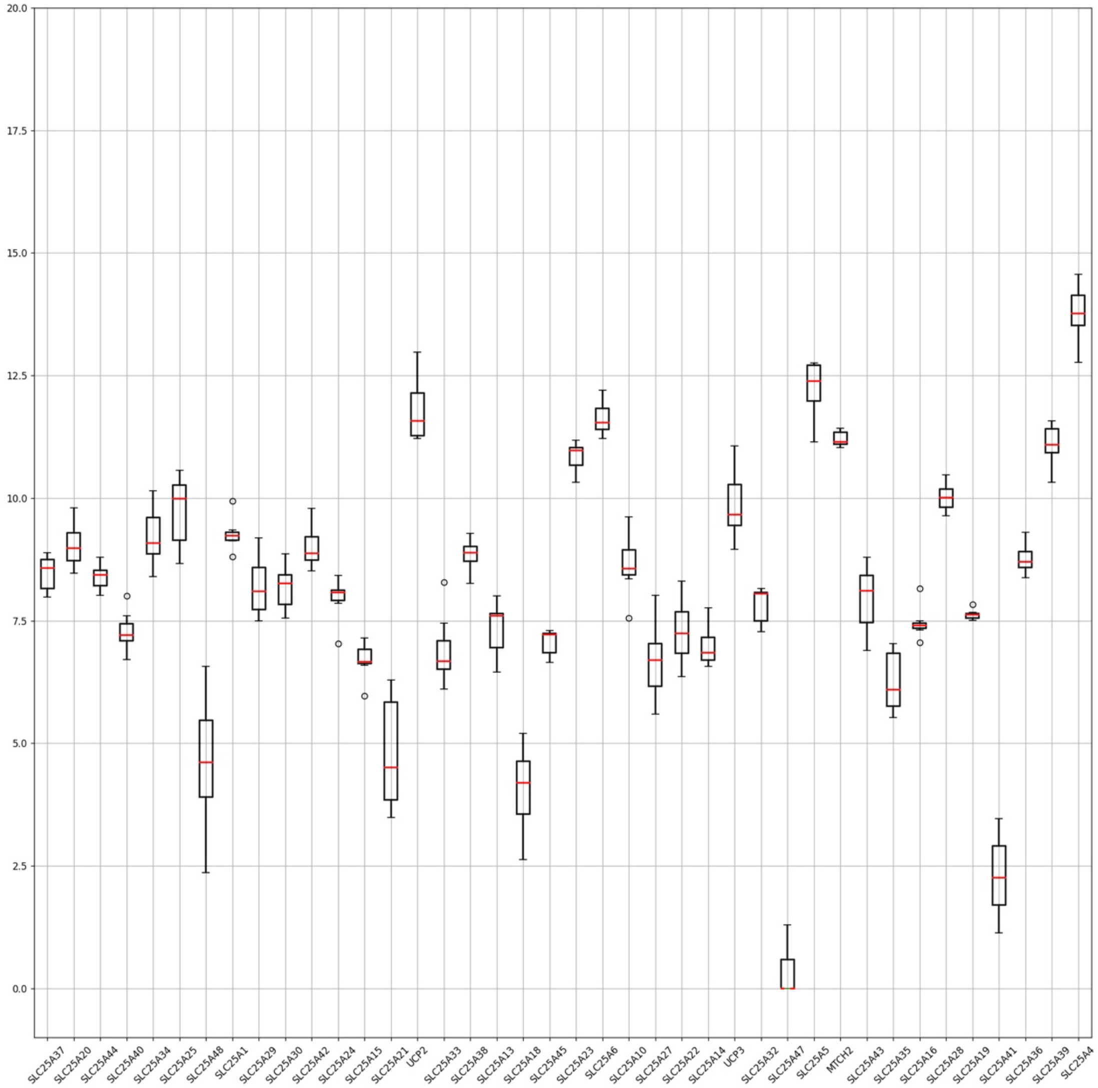

Figure S10P.

Boxplot of SLC25 transporter expression cluster 22 of GTEx normal tissues (log_2FPKM)

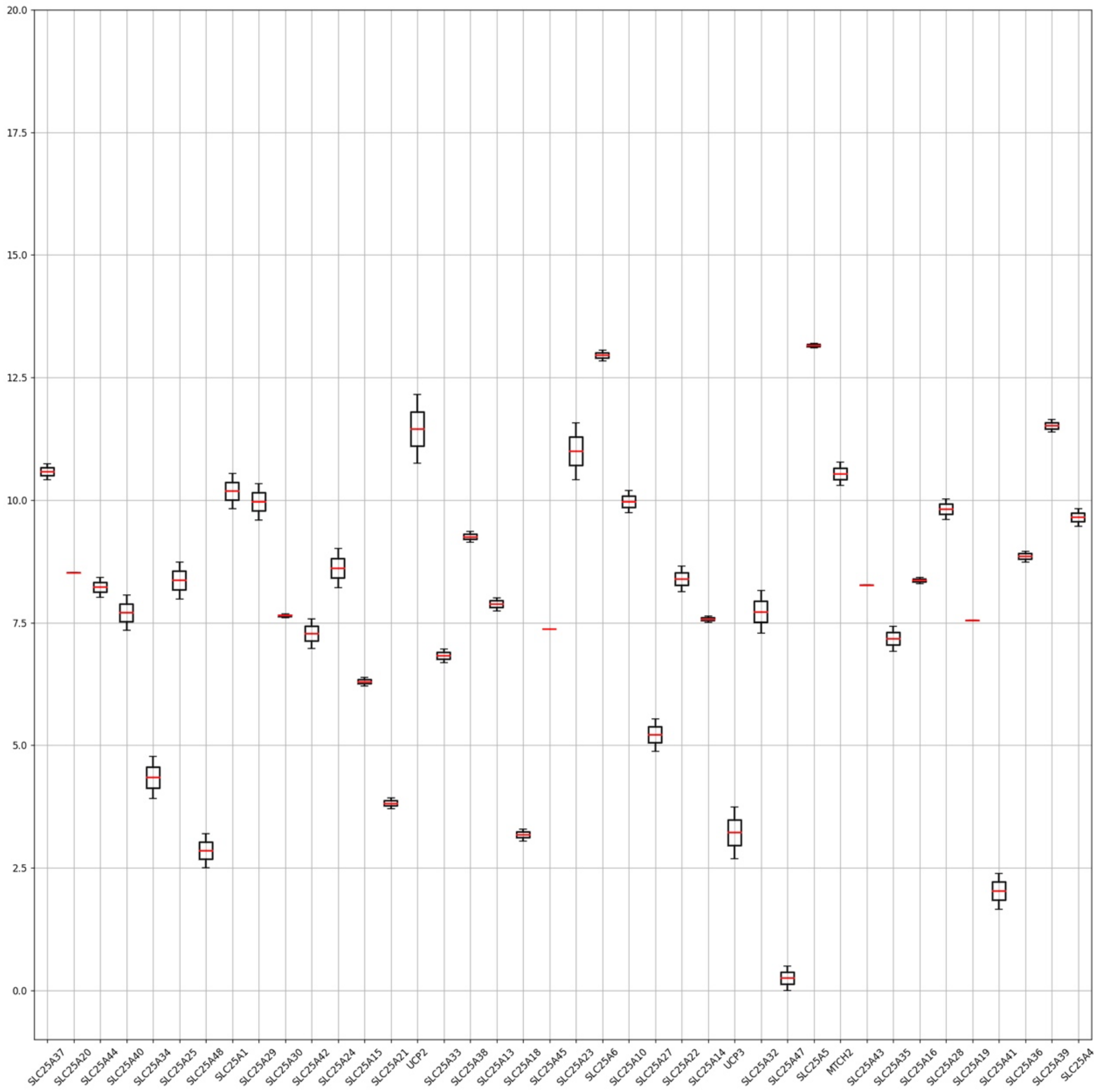

Figure S10Q.

Boxplot of SLC25 transporter expression cluster 23 of GTEx normal tissues (log_2FPKM)

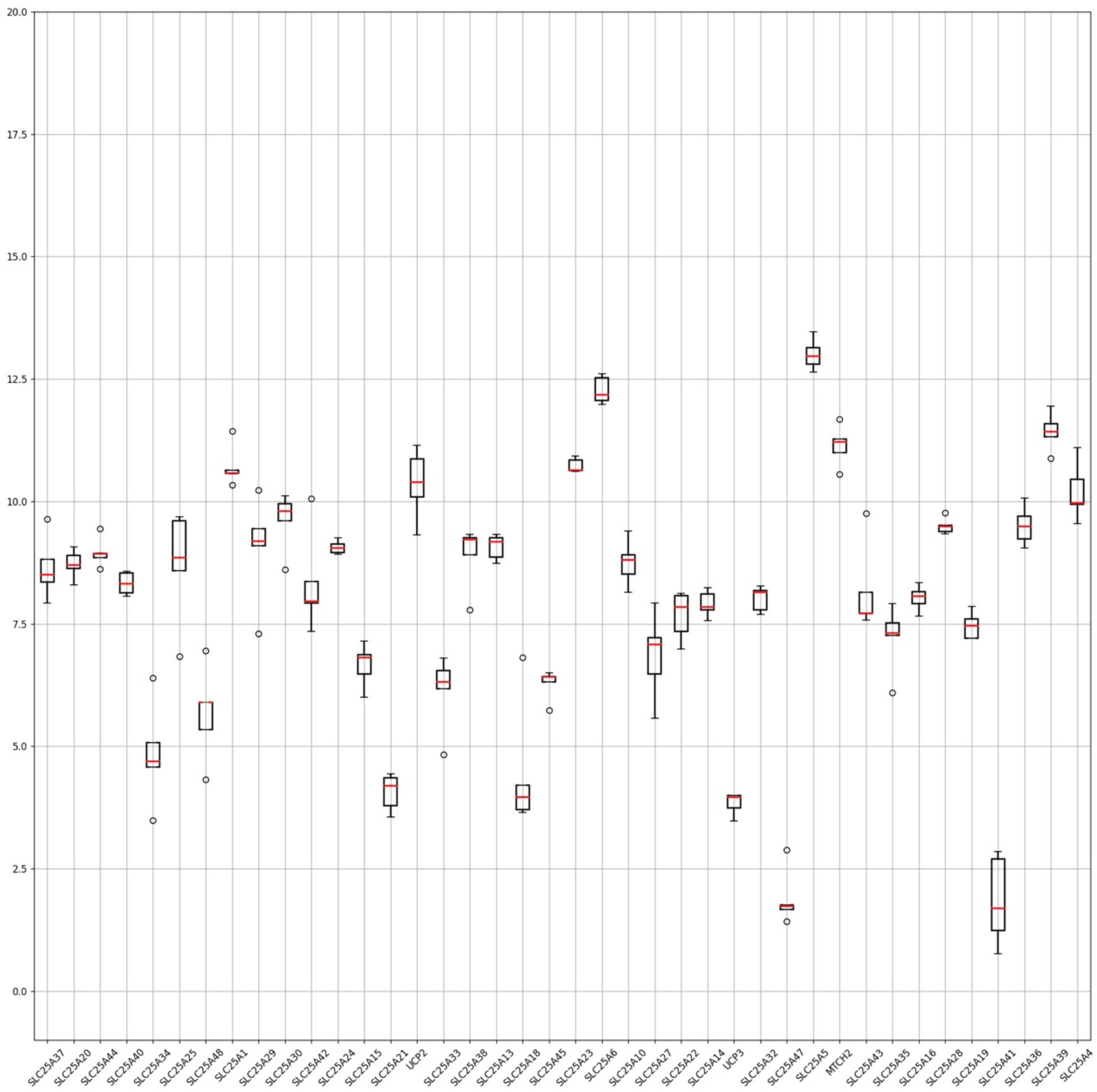

Figure S10R.

Boxplot of SLC25 transporter expression cluster 24 of GTEx normal tissues (log_2FPKM)

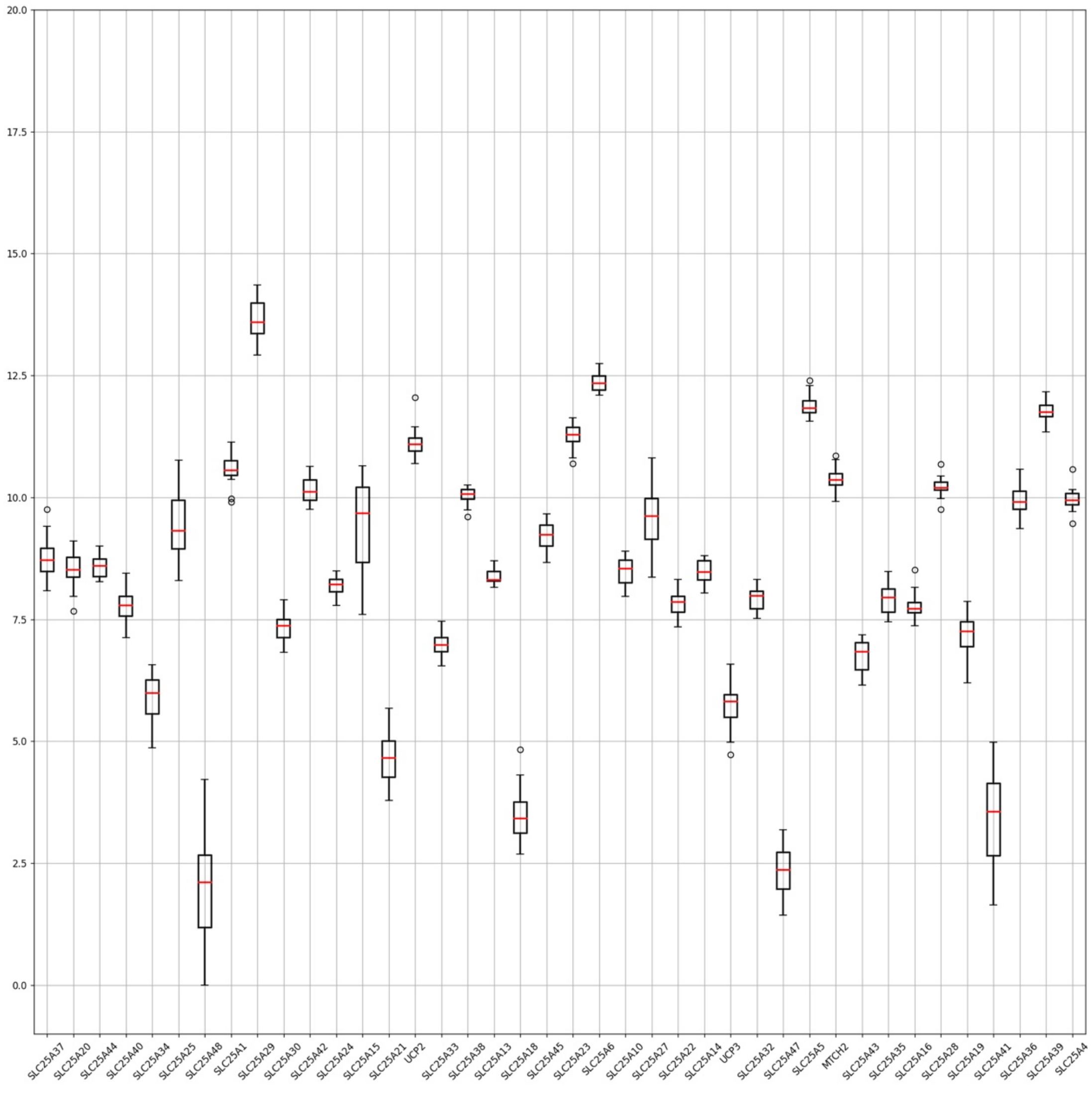

Figure S10S.

Boxplot of SLC25 transporter expression cluster 26 of GTEx normal tissues (log_2FPKM)

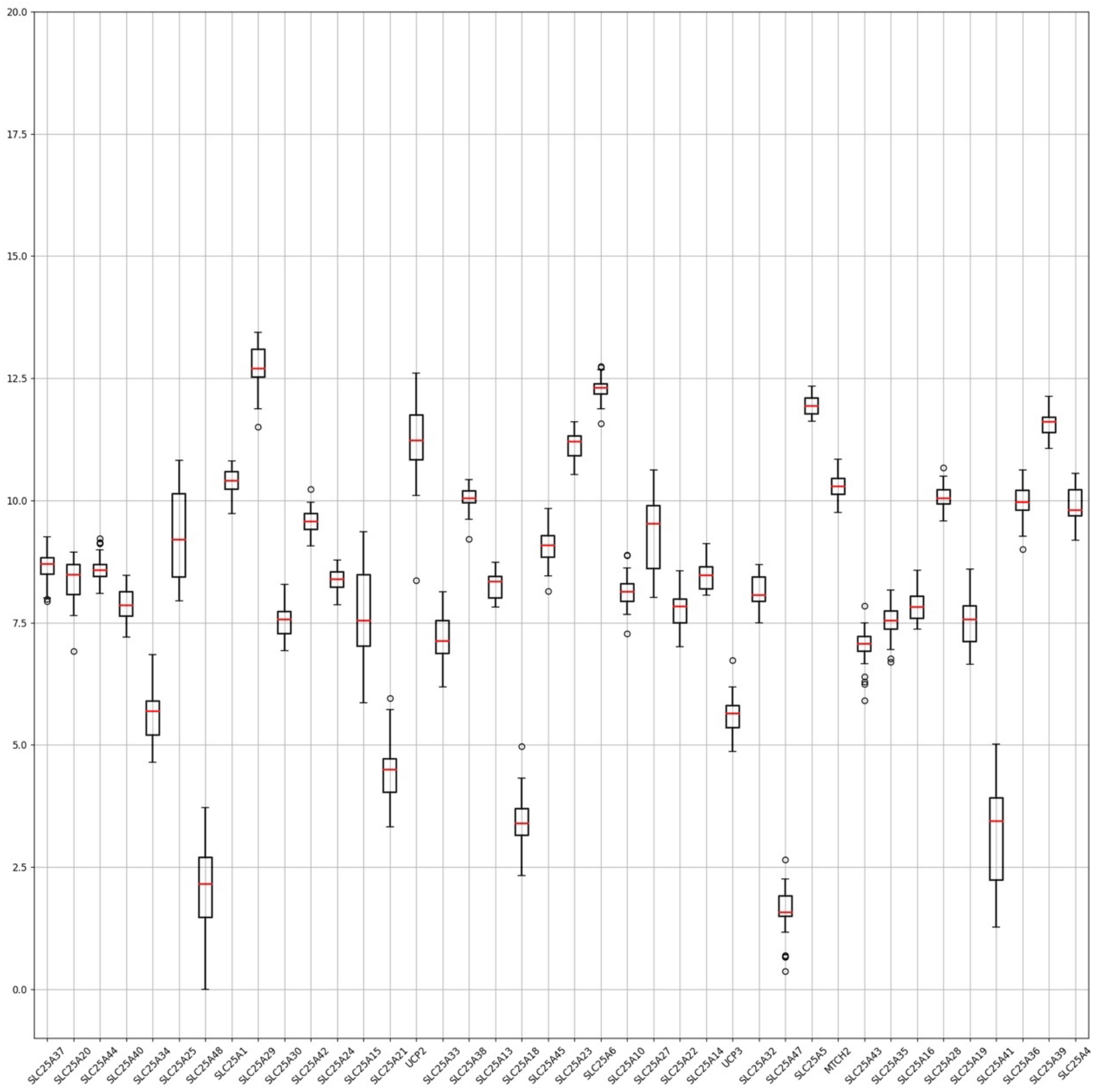

Figure S10T.

Boxplot of SLC25 transporter expression cluster 27 of GTEx normal tissues (log_2FPKM)

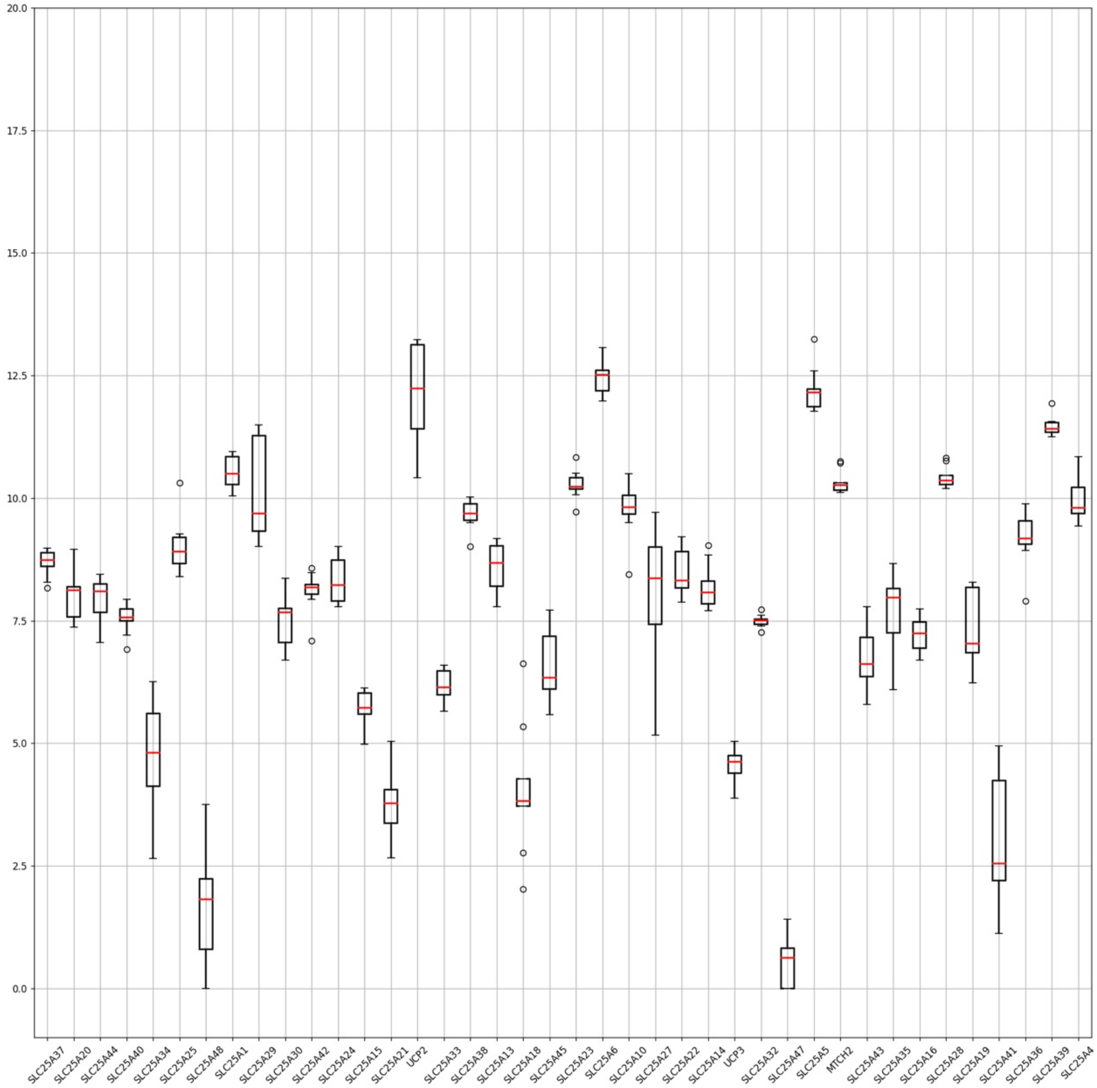

Figure S10U.

Boxplot of SLC25 transporter expression cluster 33 of GTEx normal tissues (log_2FPKM)

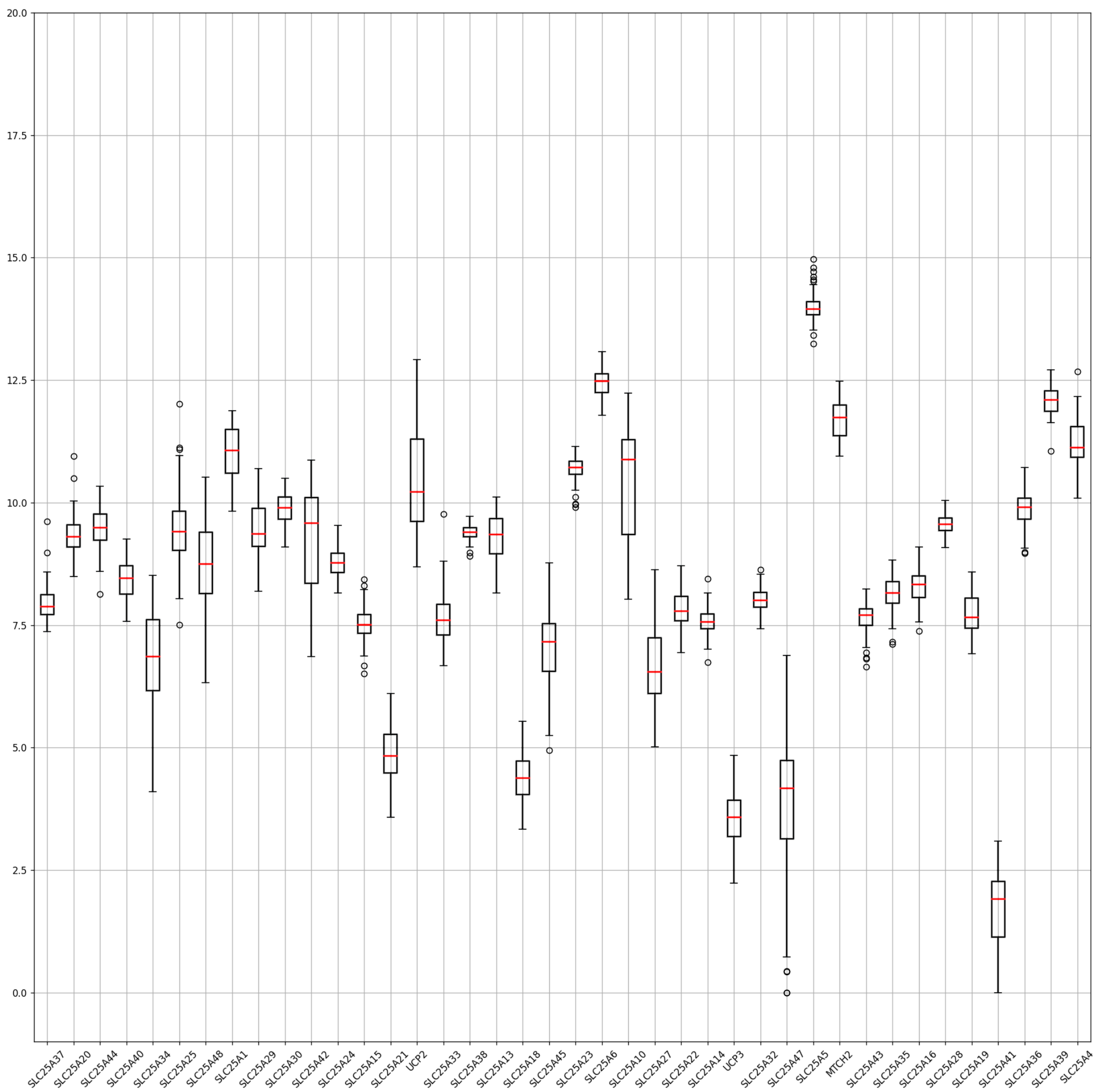

Figure S10V.

Boxplot of SLC25 transporter expression cluster 34 of GTEx normal tissues (log_2FPKM)

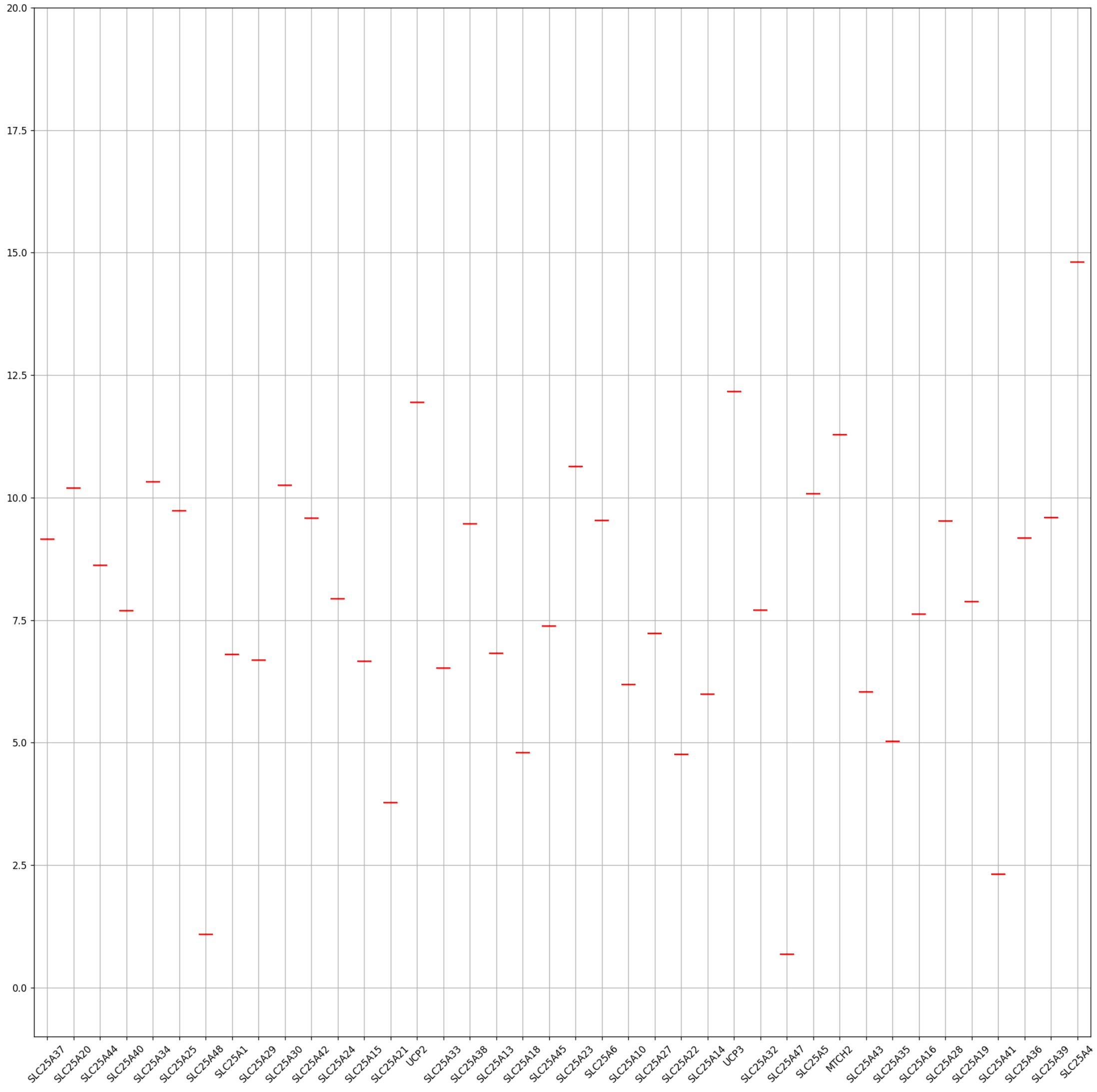

Figure S10W.

Boxplot of SLC25 transporter expression cluster 35 of GTEx normal tissues (log_2FPKM)

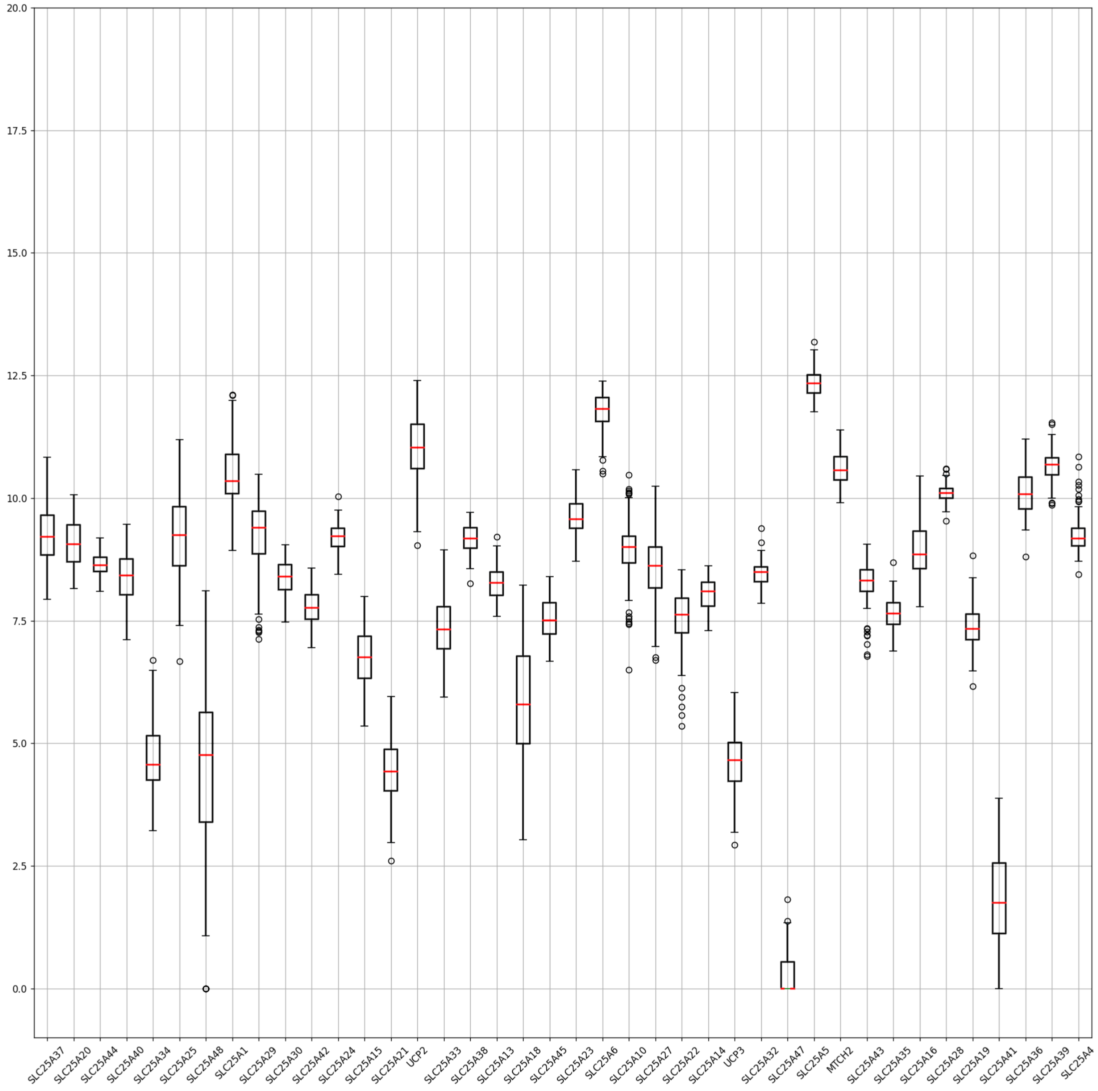

Figure S10X.

Boxplot of SLC25 transporter expression cluster 36 of GTEx normal tissues (log_2FPKM)

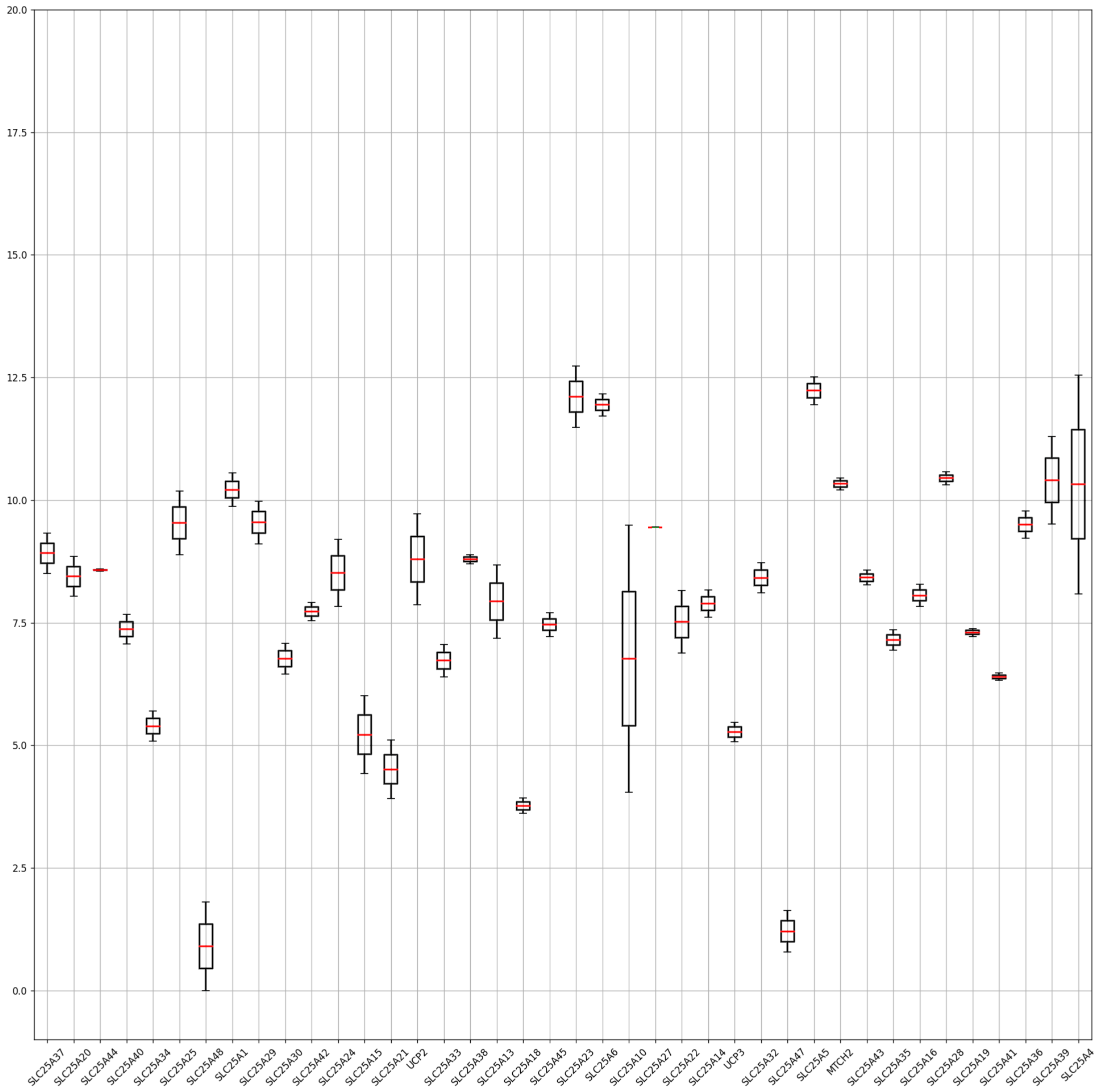

Figure S10Y.

Boxplot of SLC25 transporter expression cluster 40 of GTEx normal tissues (log_2FPKM)

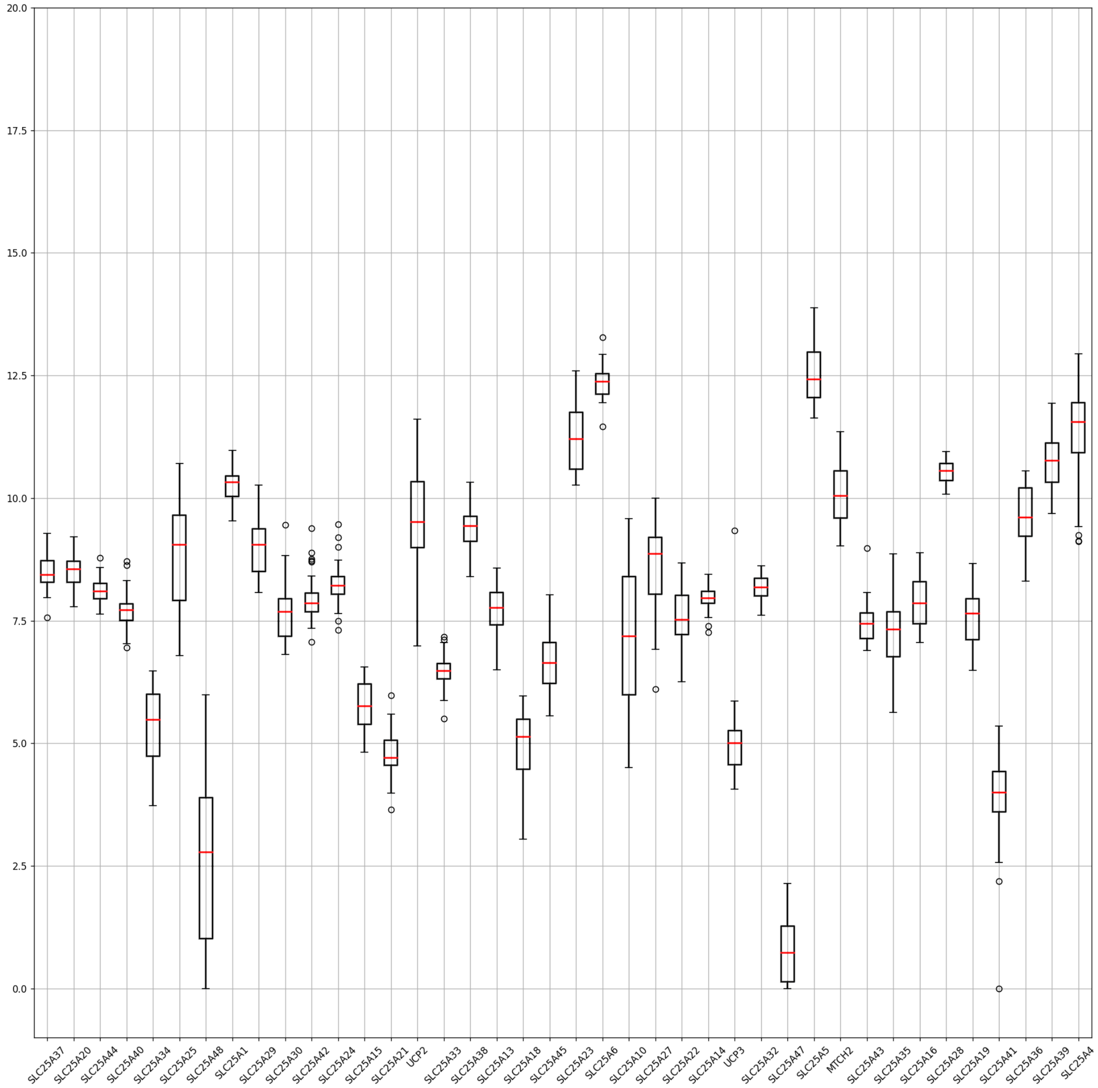

Figure S10Z.

Boxplot of SLC25 transporter expression cluster 41 of GTEx normal tissues (log_2FPKM)

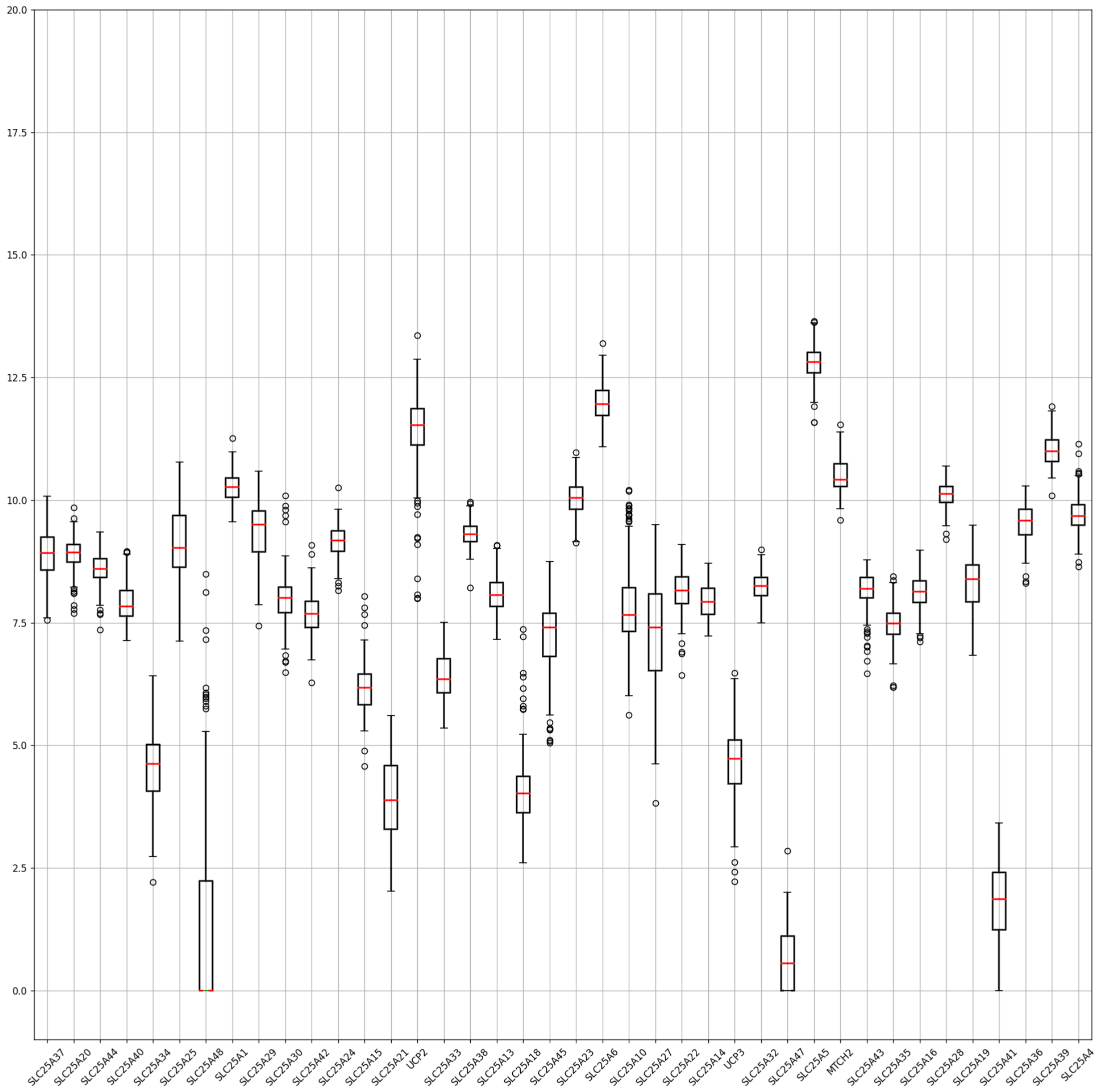

Figure S10AA.

Boxplot of SLC25 transporter expression cluster 42 of GTEx normal tissues ($\log_2$FPKM)

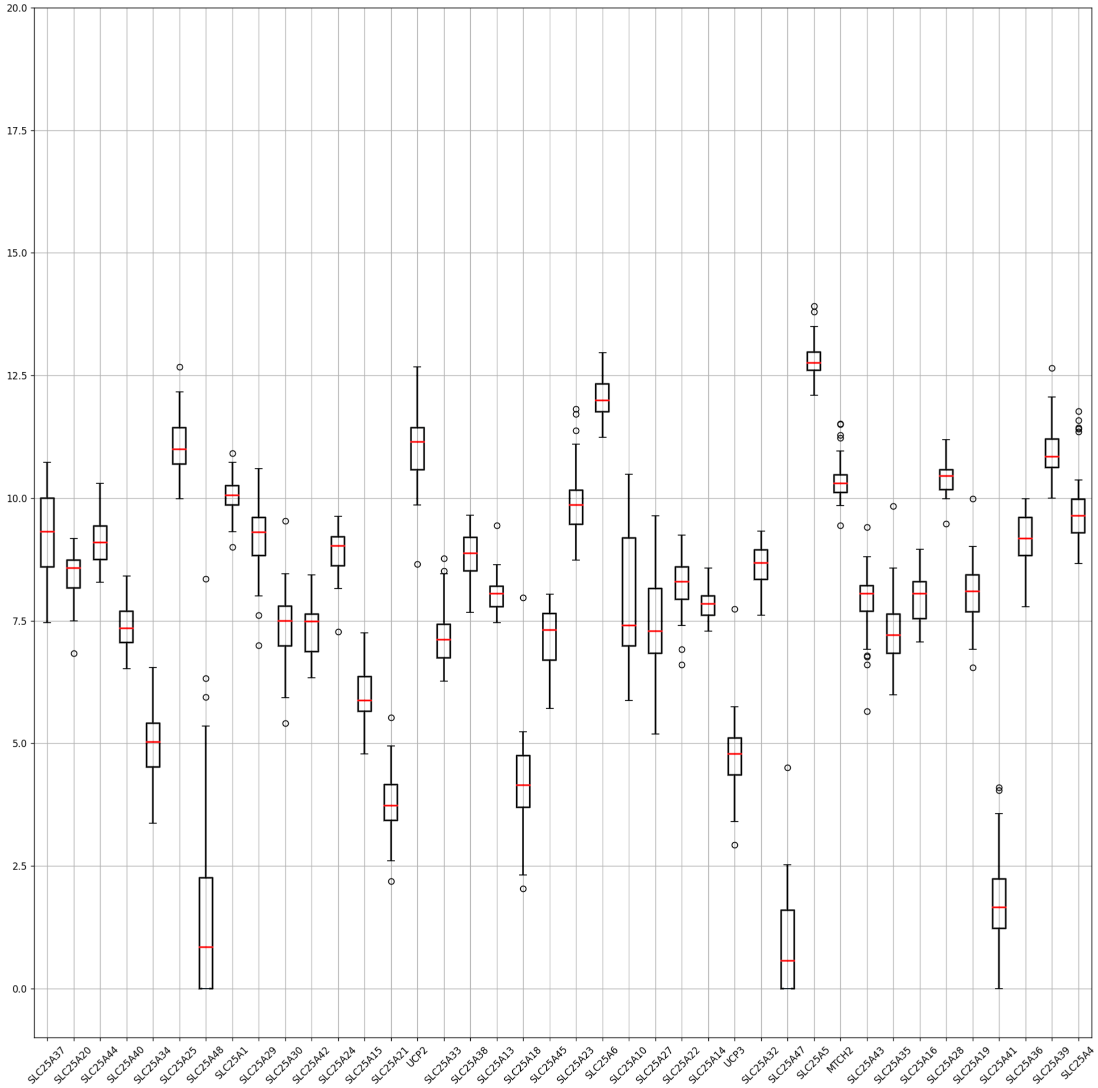

Figure S10AB.

Boxplot of SLC25 transporter expression cluster 43 of GTEx normal tissues (log_2FPKM)

Table S1. Primers for qRT-PCR of enzyme and SLC25 transporter genes

REAGENTS	SOURCE	IDENTIFIER
ALDOA (cytosolic aldolase A) Prime Time qPCR Primers	Integrated DNA Technologies	Hs.PT.56a.39231720.g standard desalting
GAC,KGA (mitochondrial and cytosolic variants of GLS1, glutaminase 1) Prime Time qPCR Primers	Integrated DNA Technologies	Hs.PT.58.3289452 standard desalting
GCLC (cytosolic glutamate-cysteine ligase catalytic subunit) Prime Time qPCR Primers	Integrated DNA Technologies	Hs.PT.58.246294 standard desalting
GLDC [mitochondrial P-protein (pyridoxal phosphate-dependent glycine decarboxylase) of the GLDC (glycine decarboxylase complex or glycine cleavage system)] Prime Time qPCR Primers	Integrated DNA Technologies	Hs.PT.58.19939816 standard desalting
GLUD1 (mitochondrial glutamate dehydrogenase) Prime Time qPCR Primers	Integrated DNA Technologies	Hs.PT.58.40215018 standard desalting
GLUL (mitochondrial glutamine synthetase) Prime Time qPCR Primers	Integrated DNA Technologies	Hs.PT.58.39372230 standard desalting
IDH1 (cytosolic isocitrate dehydrogenase 1) Prime Time qPCR Primers	Integrated DNA Technologies	Hs.PT.58.2561570 standard desalting
KGA (cytosolic variant of GLS1, glutaminase 1) Prime Time qPCR Primers	Integrated DNA Technologies	Hs.PT.58.3646381 standard desalting
LDHA (cytosolic lactate dehydrogenase A) Prime Time qPCR Primers	Integrated DNA Technologies	Hs.PT.58.38690093 standard desalting
MPC1 (mitochondrial pyruvate transporter) Prime Time qPCR Primers	Integrated DNA Technologies	Hs.PT.58.46455699 standard desalting
MTFMT (mitochondrial methionyl-tRNA formyl transferase) Prime Time qPCR Primers	Integrated DNA Technologies	Hs.PT.58.27867496 standard desalting
MTHFD1 (cytosolic trifunctional c-1-tetrahydrofolate synthase) Prime Time qPCR Primers	Integrated DNA Technologies	Hs.PT.58.22984373 standard desalting
MTHFD2 (mitochondrial bifunctional methylenetetrahydrofolate dehydrogenase / cyclohydrolase) Prime Time qPCR Primers	Integrated DNA Technologies	Hs.PT.58.27454737 standard desalting
MTHFD2L (location and the catalytic properties of human MTHFD2 but expressed more ubiquitously) Prime Time qPCR Primers	Integrated DNA Technologies	Hs.PT.58.38398095 standard desalting
PC (mitochondrial pyruvate carboxylase) Prime Time qPCR Primers	Integrated DNA Technologies	Hs.PT.58.3296529 standard desalting
PKM1, PKM2 (splice variants of human cytosolic pyruvate kinase) Prime Time qPCR Primers	Integrated DNA Technologies	Hs.PT.58.14798218 standard desalting
PKM1 (splice variants of human cytosolic pyruvate kinase) Prime Time qPCR Primers	Integrated DNA Technologies	Hs.PT.58.561234 standard desalting
PPIA (peptidyl-prolyl cis-trans isomerase A) Prime Time qPCR Primers	Integrated DNA Technologies	Hs.PT.59a.22214851 standard desalting

SHMT1 (cytosolic serine hydroxymethyltransferase) Prime Time qPCR Primers	Integrated DNA Technologies	Hs.PT.58.24984705 standard desalting
SHMT2 (mitochondrial serine hydroxymethyltransferase) Prime Time qPCR Primers	Integrated DNA Technologies	Hs.PT.58.40911690 standard desalting
SLC25A1 Prime Time qPCR Primers	Integrated DNA Technologies	Hs.PT.58.45315671 standard desalting
SLC25A15 QuantiTect Primer Assays	Qiagen	Cat# 249900 QT00062867
SLC25A2 QuantiTect Primer Assays	Qiagen	Cat# 249900 QT00203931
SLC25A3 QuantiTect Primer Assays	Qiagen	Cat# 249900 QT00036043
SLC25A4 QuantiTect Primer Assays	Qiagen	Cat# 249900 QT00048279
SLC25A5 QuantiTect Primer Assays	Qiagen	Cat# 249900 QT00084189
SLC25A6 QuantiTect Primer Assays	Qiagen	Cat# 249900 QT00037191
SLC25A31 Prime Time qPCR Primers	Integrated DNA Technologies	Hs.PT.58.38424672 standard desalting
SLC25A7 Prime Time qPCR Primers	Integrated DNA Technologies	Hs.PT.58.39157006 standard desalting
SLC25A8 Prime Time qPCR Primers	Integrated DNA Technologies	Hs.PT.58.15294923 standard desalting
SLC25A9 Prime Time qPCR Primers	Integrated DNA Technologies	Hs.PT.58.23089893 standard desalting
SLC25A10 QuantiTect Primer Assays	Qiagen	Cat# 249900 QT00073724
SLC25A11 Prime Time qPCR Primers	Integrated DNA Technologies	Hs.PT.58.39807718 standard desalting
SLC25A12 QuantiTect Primer Assays	Qiagen	Cat# 249900 QT00073010
SLC25A13 QuantiTect Primer Assays	Qiagen	Cat# 249900 QT00087906
SLC25A14 QuantiTect Primer Assays	Qiagen	Cat# 249900 QT00040544
SLC25A30 QuantiTect Primer Assays	Qiagen	Cat# 249900 QT00080941
SLC25A16 QuantiTect Primer Assays	Qiagen	Cat# 249900 QT00037849
SLC25A43 Prime Time qPCR Primers	Integrated DNA Technologies	Hs.PT.58.39634062 standard desalting
SLC25A17 QuantiTect Primer Assays	Qiagen	Cat# 249900 QT00075502
SLC25A22 Prime Time qPCR Primers	Integrated DNA Technologies	Hs.PT.58.14419178 standard desalting

SLC25A18 Prime Time qPCR Primers	Integrated DNA Technologies	Hs.PT.58.1426160 standard desalting
SLC25A19 QuantiTect Primer Assays	Qiagen	Cat# 249900 QT00077490
SLC25A20 Prime Time qPCR Primers	Integrated DNA Technologies	Hs.PT.56a.21374355 standard desalting
SLC25A21 Prime Time qPCR Primers	Integrated DNA Technologies	Hs.PT.58.28258655 standard desalting
SLC25A23 QuantiTect Primer Assays	Qiagen	Cat# 249900 QT00044107
SLC25A24a QuantiTect Primer Assays	Qiagen	Cat# 249900 QT01019606
SLC25A24b QuantiTect Primer Assays	Qiagen	Cat# 249900 QT01019613
SLC25A25 QuantiTect Primer Assays	Qiagen	Cat# 249900 QT00002422
SLC25A41 QuantiTect Primer Assays	Qiagen	Cat# 249900 QT00052577
SLC25A26 QuantiTect Primer Assays	Qiagen	Cat# 249900 QT01029497
SLC25A27 QuantiTect Primer Assays	Qiagen	Cat# 249900 QT00009100
SLC25A28 QuantiTect Primer Assays	Qiagen	Cat# 249900 QT00052801
SLC25A37 QuantiTect Primer Assays	Qiagen	Cat# 249900 QT00020559
SLC25A29 QuantiTect Primer Assays	Qiagen	Cat# 249900 QT00231623
SLC25A32 Prime Time qPCR Primers	Integrated DNA Technologies	Hs.PT.58.26726272 standard desalting
SLC25A33 Prime Time qPCR Primers	Integrated DNA Technologies	Hs.PT.58.20602154 standard desalting
SLC25A36 QuantiTect Primer Assays	Qiagen	Cat# 249900 QT00026208
SLC25A34 QuantiTect Primer Assays	Qiagen	Cat# 249900 QT01033942
SLC25A35 Prime Time qPCR Primers	Integrated DNA Technologies	Hs.PT.58.23165318 standard desalting
SLC25A38 Prime Time qPCR Primers	Integrated DNA Technologies	Hs.PT.58.1061544 standard desalting
SLC25A39 QuantiTect Primer Assays	Qiagen	Cat# 249900 QT00083839
SLC25A40 QuantiTect Primer Assays	Qiagen	Cat# 249900 QT00040698
SLC25A42 QuantiTect Primer Assays	Qiagen	Cat# 249900 QT00041412
SLC25A44 QuantiTect Primer Assays	Qiagen	Cat# 249900 QT00093065
SLC25A45 QuantiTect Primer Assays	Qiagen	Cat# 249900 QT00060473

SLC25A46 QuantiTect Primer Assays	Qiagen	Cat# 249900 QT00013041
SLC25A47 Prime Time qPCR Primers	Integrated DNA Technologies	Hs.PT.58.25038599.g standard desalting
SLC25A48 Prime Time qPCR Primers	Integrated DNA Technologies	Hs.PT.58.39281677 standard desalting
SLC25A49 Prime Time qPCR Primers	Integrated DNA Technologies	Hs.PT.58.45375549.g standard desalting
SLC25A50 Prime Time qPCR Primers	Integrated DNA Technologies	Hs.PT.58.15104870 standard desalting
SLC25A51 QuantiTect Primer Assays	Qiagen	Cat# 249900 QT00202328
SLC25A52 QuantiTect Primer Assays	Qiagen	Cat# 249900 QT00271565
SLC25A53 Prime Time qPCR Primers	Integrated DNA Technologies	Hs.PT.58.4275637 standard desalting

Table S2. Oligonucleotides and Recombinant DNA

Reagent	Source	Identifier
Lentiviral pZIP-TRE3G - V125 vector encoding tetracycline inducible shRNA targeting human SLC25A22 TGCTGTTGACAGTGAGCGACATGTCCGACTGCCTCATCAATAGTGAAGCCACAGATGTATTGATGAGGCAGTCGGACATGCTGCCTACTGCCTCGGA	transOMIC technologies	ULTRA-3395*753*
Lentiviral pZIP-TRE3G - V125 vector encoding tetracycline inducible shRNA targeting human SLC25A22 TGCTGTTGACAGTGAGCGCCCCCTTCTCTGTGGTGTACTTTAGTGAAGCCACAGATGTAAAGTACACCACAGAGAAGGGGATGCCTACTGCCTCGGA	transOMIC technologies	ULTRA-3395755
Lentiviral pZIP-TRE3G - V125 vector encoding tetracycline inducible shRNA targeting human SLC25A22 TGCTGTTGACAGTGAGCGAAGAAGTCGCCTTTCTACGTGATAGTGAAGCCACAGATGTATCACGTAGAAAGGCGACTTCTCTGCCTACTGCCTCGGA	transOMIC technologies	ULTRA-3395*752*
Lentiviral pZIP-TRE3G - V125 vector encoding tetracycline inducible shRNA targeting human SLC25A32 TGCTGTTGACAGTGAGCGCCCGAAATATAATGGAATTTTATAGTGAAGCCACAGATGTATAAAATTCCATTATATTTCGGTTGCCTACTGCCTCGGA	transOMIC technologies	ULTRA-3398782
Lentiviral pZIP-TRE3G - V125 vector encoding tetracycline inducible shRNA targeting human SLC25A32 TGCTGTTGACAGTGAGCGCACAGTATGATGCTGTTGTTAATAGTGAAGCCACAGATGTATTAACAACAGCATCATACTGTATGCCTACTGCCTCGGA	transOMIC technologies	ULTRA-3398780
Lentiviral pZIP-TRE3G - V125 vector encoding tetracycline inducible shRNA targeting human SLC25A32 TGCTGTTGACAGTGAGCGCGTTGAGCACAGTAGAATATAATAGTGAAGCCACAGATGTATTATATTCTACTGTGCTCAACTTGCCTACTGCCTCGGA	transOMIC technologies	ULTRA-3398783
Lentiviral pZIP-TRE3G - V125 vector encoding tetracycline inducible shRNA targeting human SLC25A43 TGCTGTTGACAGTGAGCGCTAGCACCTTTGAGTTCTGCAATAGTGAAGCCACAGATGTATTGCAGAACTCAAAGGTGCTAATGCCTACTGCCTCGGA	transOMIC technologies	ULTRA-3236*863*
Lentiviral pZIP-TRE3G - V125 vector encoding tetracycline inducible shRNA targeting human SLC25A43 TGCTGTTGACAGTGAGCGACCAGGAATTACGAGAATTAAATAGTGAAGCCACAGATGTATTTAATTCTCGTAATTCCTGGGTGCCTACTGCCTCGGA	transOMIC technologies	ULTRA-3236*859*
Lentiviral pZIP-TRE3G - V125 vector encoding tetracycline inducible shRNA targeting human SLC25A43 TGCTGTTGACAGTGAGCGCTGGTTACATTCTGTCTCCACATAGTGAAGCCACAGATGTATGTGGAGACAGAATGTAACCATTGCCTACTGCCTCGGA	transOMIC technologies	ULTRA-3236864
Lentiviral pZIP-TRE3G - V125	transOMIC technologies	N/A

psPAX2	Addgene	Plasmid #12260
pCMV-VSV-G	Addgene	Plasmid #8485

Table S3. Results of the F1 scores for SVM, KNN, RF, GBT and GNB tests

Classification model	Run 1	Run 2	Run 3	Run 4	Run 5
SVM	0.86413245	0.80966841	0.85548812	0.85793773	0.85617276
KNN	0.74035855	0.71975537	0.74828806	0.75209747	0.75324551
RF	0.68800053	0.6846961	0.69532281	0.71585688	0.70805913
GBT	0.732525	0.75331907	0.74607151	0.75168713	0.75039186
GNB	0.64639778	0.61877729	0.63198712	0.66647984	0.64625803

Table S4. The results of the test against the reserved test data set. Precision: TP / (TP + FP); Recall: TP / (TP + FN); F1-score: TP / (TP + 1/2 * (FP + FN)); Support: number of samples. The macro avg is the mean of all the scores, while the weighted avg is the mean score adjusted by the support.

cluster	precision	recall	f1-score	support
0	0.96	0.99	0.97	87
1	0.91	0.95	0.93	99
2	1.00	0.73	0.84	22
3	0.86	0.86	0.86	172
4	0.83	1.00	0.91	5
5	0.81	0.76	0.79	34
6	0.94	0.93	0.93	68
7	0.84	0.89	0.86	149
8	0.90	0.80	0.85	66
9	0.90	0.76	0.83	34
10	1.00	1.00	1.00	4
11	0.87	0.91	0.89	53
12	0.83	0.87	0.85	52
13	1.00	0.97	0.98	29
14	0.88	0.91	0.89	172
15	1.00	0.83	0.91	6
16	0.86	0.93	0.89	99
17	0.87	0.87	0.87	15
19	1.00	0.33	0.50	3
20	0.92	0.90	0.91	116
21	0.84	0.86	0.85	174
22	0.75	0.75	0.75	4
23	0.88	0.78	0.83	58
24	0.88	0.99	0.93	141
26	0.96	1.00	0.98	26
27	0.97	0.90	0.93	62
31	0.88	0.93	0.91	162
32	0.87	0.91	0.89	141
33	0.96	0.95	0.96	124
34	0.95	0.93	0.94	56
36	0.92	0.80	0.86	60
38	0.86	0.83	0.84	246
39	1.00	0.80	0.89	5
40	0.75	1.00	0.86	6
41	1.00	0.77	0.87	22
42	0.88	0.83	0.85	140
43	0.93	0.76	0.84	17
accuracy			0.89	2729
macro avg	0.90	0.86	0.88	2729
weighted avg	0.89	0.89	0.89	2729

Table S5. Results tested against the GTEx data set that was filtered from use in the training data

cluster	precision	recall	f1-score	support
0	0.67	1.00	0.80	4
1	0.89	0.89	0.89	65
2	0.67	0.87	0.75	53
3	0.92	0.85	0.88	40
4	1.00	0.85	0.92	100
5	0.78	0.81	0.80	239
6	1.00	0.75	0.86	4
7	0.76	0.73	0.75	26
8	0.40	0.50	0.44	4
9	0.00	0.00	0.00	0
10	0.86	0.80	0.83	15
11	1.00	1.00	1.00	1
12	0.71	0.95	0.82	21
13	0.00	0.00	0.00	0
14	0.57	0.78	0.66	36
15	1.00	1.00	1.00	3
16	0.85	0.85	0.85	27
17	0.88	1.00	0.93	7
18	0.00	0.00	0.00	1
19	0.49	0.79	0.60	107
20	0.96	0.90	0.93	162
21	0.84	0.81	0.83	194
22	0.75	1.00	0.86	3
23	0.84	0.82	0.83	39
24	0.25	0.40	0.31	5
26	0.97	0.96	0.97	141
27	0.91	0.93	0.92	168
28	0.00	0.00	0.00	1
29	0.00	0.00	0.00	12
30	0.00	0.00	0.00	8
31	0.33	0.33	0.33	3
32	0.00	0.00	0.00	0
33	0.44	0.78	0.56	9
34	0.86	1.00	0.92	18
36	0.71	0.88	0.78	90
38	0.53	0.56	0.55	16
39	1.00	0.40	0.57	5
40	0.74	0.29	0.41	80
41	0.89	0.78	0.83	387
42	0.67	0.80	0.73	142
43	0.86	0.66	0.75	86
accuracy			0.80	2322
macro avg	0.63	0.65	0.63	2322
weighted avg	0.82	0.80	0.80	2322

Table S6. Human mitochondrial SLC25 transporter family

SLC25	Name / Substrate	SLC25	Name / Substrate	SLC25	Name / Substrate
A1[1,2]	citrate, CTP	A43[3]	?	A33[4]	PNC1
A15[5]	ornithine, ORNT1	A17*[6]	CoA, FAD, NAD+	A36[7]	PNC2
A2[8]	ornithine, ORNT2	A22[9]	glutamate 1	A34[10]	?
A3[11,12]	phosphate, Cu, PTP	A18[13]	glutamate 2	A35[14]	?
A4[15-17]	ADP/ATP, T1	A19[18]	thiamine PP	A38[19]	glycine
A5[20]	ADP/ATP, T2	A20[21]	acylcarnitine	A39[22]	glutathione, heme
A6[23]	ADP/ATP, T3	A21[24,25]	2-oxodicarboxylate	A40[26]	MCFP
A31[27]	ADP/ATP, T4	A23[28]	APC2	A42[29,30]	CoA, ADP
A7[31]	UCP1	A24b[32]	APC1iso2	A44[33]	BCAA
A8[34]	UCP2, C4 metabolites	A24a[32]	APC1iso1	A45[35]	?
A9[36]	UCP3	A25[37]	APC3	A46[38]	mito fission
A10[39]	dicarboxylate	A41[40]	APC4	A47[41]	HDMCP, UCP?
A11[42]	oxoglutarate-malate	A26[43,44]	SAM	A48[45]	?
A12[46]	asp-glu, aralar 1	A27[47]	UCP4	A49[48]	MTCH1apo
A13[49]	asp-glu, aralar 2	A28[50]	mitoferrin2	A50[51]	MTCH2adi
A14[52]	UCP5, BMCP1	A37[53]	mitoferrin1	A51[54]	NAD$^+$,MCART1
A30[55]	KMPC1	A29[56,57]	BAA	A52[14]	MCART2
A16[58]	CoA,Grave's disease	A32[59]	THF	A53[60]	MCART6

Note: transporters grouped between red lines have high sequence similarity, i.e. they have at least one transmembrane helix with similarity E-value of less than 1e-04. Name/Substrate (transporter name or transported substrate). BMCP1 (brain mitochondrial carrier protein 1), KMCP1 (kidney mitochondrial carrier protein 1), A17* (peroxisomal membrane protein 34, the only known SLC25 transporter expressed in peroxisomes and not in mitochondria), A24a and b isoforms differ in their N-terminal sequences (about 50 residues) and are shown in most data as only A24, BAA (basic amino acids), THF (tetrahydrofolate), MCFP (mitochondrial carrier family protein), BCAA (branched-chain amino acids), HDMCP (hepatocellular carcinoma-down regulated mitochondrial carrier protein), MTCH1apo (apoptosis-related), MTCH2adi (adipocyte-related). The SLC25 transporters are referred to in the text without the SLC25.

References to Table S6

1 Kaplan, R. S., Mayor, J. A., Gremse, D. A. & Wood, D. O. High level expression and characterization of the mitochondrial citrate transport protein from the yeast Saccharomyces cerevisiae. *J. Biol. Chem.* **270**, 4108-4114 (1995). https://doi.org:10.1074/jbc.270.8.4108

2 Fernandez, H. R. *et al.* The mitochondrial citrate carrier, SLC25A1, drives stemness and therapy resistance in non-small cell lung cancer. *Cell Death Differ.* **25**, 1239-1258 (2018). https://doi.org:10.1038/s41418-018-0101-z

3 Gabrielson, M., Reizer, E., Stal, O. & Tina, E. Mitochondrial regulation of cell cycle progression through SLC25A43. *Biochem. Biophys. Res. Commun.* **469**, 1090-1096 (2016). https://doi.org:10.1016/j.bbrc.2015.12.088

4 Favre, C., Zhdanov, A., Leahy, M., Papkovsky, D. & O'Connor, R. Mitochondrial pyrimidine nucleotide carrier (PNC1) regulates mitochondrial biogenesis and the invasive phenotype of cancer cells. *Oncogene* **29**, 3964-3976 (2010). https://doi.org:10.1038/onc.2010.146

5 Ji, S. M. Overexpression of SLC25A15 is involved in the proliferation of cutaneous melanoma and leads to poor prognosis. *Med. Sci. (Paris)* **34 Focus issue F1**, 74-80 (2018). https://doi.org:10.1051/medsci/201834f113

6 Agrimi, G., Russo, A., Scarcia, P. & Palmieri, F. The human gene SLC25A17 encodes a peroxisomal transporter of coenzyme A, FAD and NAD+. *Biochem. J.* **443**, 241-247 (2012). https://doi.org:10.1042/BJ20111420

7 Cavanillas, M. L. *et al.* Replication of top markers of a genome-wide association study in multiple sclerosis in Spain. *Genes Immun.* **12**, 110-115 (2011). https://doi.org:10.1038/gene.2010.52
8 Camacho, J. A., Rioseco-Camacho, N., Andrade, D., Porter, J. & Kong, J. Cloning and characterization of human ORNT2: a second mitochondrial ornithine transporter that can rescue a defective ORNT1 in patients with the hyperornithinemia-hyperammonemia-homocitrullinuria syndrome, a urea cycle disorder. *Mol. Genet. Metab.* **79**, 257-271 (2003). https://doi.org:10.1016/s1096-7192(03)00105-7
9 Wong, C. C. *et al.* In Colorectal Cancer Cells With Mutant KRAS, SLC25A22-Mediated Glutaminolysis Reduces DNA Demethylation to Increase WNT Signaling, Stemness, and Drug Resistance. *Gastroenterology* **159**, 2163-2180 e2166 (2020). https://doi.org:10.1053/j.gastro.2020.08.016
10 Lawlor, N. *et al.* Single-cell transcriptomes identify human islet cell signatures and reveal cell-type-specific expression changes in type 2 diabetes. *Genome Res.* **27**, 208-222 (2017). https://doi.org:10.1101/gr.212720.116
11 Wohlrab, H. Purification and reconstitution of the mitochondrial phosphate transporter. *Ann. N. Y. Acad. Sci.* **358**, 364-367 (1980). https://doi.org:10.1111/j.1749-6632.1980.tb15423.x
12 Zhu, X. *et al.* Mitochondrial copper and phosphate transporter specificity was defined early in the evolution of eukaryotes. *Elife* **10** (2021). https://doi.org:10.7554/eLife.64690
13 Knijnenburg, J. *et al.* A 600 kb triplication in the cat eye syndrome critical region causes anorectal, renal and preauricular anomalies in a three-generation family. *Eur. J. Hum. Genet.* **20**, 986-989 (2012). https://doi.org:10.1038/ejhg.2012.43
14 Giurato, G. *et al.* Quantitative mapping of RNA-mediated nuclear estrogen receptor beta interactome in human breast cancer cells. *Sci Data* **5**, 180031 (2018). https://doi.org:10.1038/sdata.2018.31
15 Trisolini, L. *et al.* Differential Expression of ADP/ATP Carriers as a Biomarker of Metabolic Remodeling and Survival in Kidney Cancers. *Biomolecules* **11** (2020). https://doi.org:10.3390/biom11010038
16 Zhao, L. *et al.* Conformational change of adenine nucleotide translocase-1 mediates cisplatin resistance induced by EBV-LMP1. *EMBO Mol. Med.* **13**, e14072 (2021). https://doi.org:10.15252/emmm.202114072
17 Klingenberg, M. The ADP and ATP transport in mitochondria and its carrier. *Biochim. Biophys. Acta* **1778**, 1978-2021 (2008). https://doi.org:10.1016/j.bbamem.2008.04.011
18 Sabui, S., Subramanian, V. S., Kapadia, R. & Said, H. M. Structure-function characterization of the human mitochondrial thiamin pyrophosphate transporter (hMTPPT; SLC25A19): Important roles for Ile(33), Ser(34), Asp(37), His(137) and Lys(291). *Biochim. Biophys. Acta* **1858**, 1883-1890 (2016). https://doi.org:10.1016/j.bbamem.2016.05.011
19 Fan, Z. *et al.* SLC25A38 as a novel biomarker for metastasis and clinical outcome in uveal melanoma. *Cell Death Dis.* **13**, 330 (2022). https://doi.org:10.1038/s41419-022-04718-8
20 Oishi, M. *et al.* Apigenin sensitizes prostate cancer cells to Apo2L/TRAIL by targeting adenine nucleotide translocase-2. *PLoS One* **8**, e55922 (2013). https://doi.org:10.1371/journal.pone.0055922
21 Gurbuz, B. B. *et al.* Clinical and molecular characteristics of carnitineacylcarnitine translocase deficiency with c.270delC and a novel c.408C>A variant. *Turk. J. Pediatr.* **63**, 691-696 (2021). https://doi.org:10.24953/turkjped.2021.04.017
22 Wang, Y. *et al.* SLC25A39 is necessary for mitochondrial glutathione import in mammalian cells. *Nature* **599**, 136-140 (2021). https://doi.org:10.1038/s41586-021-04025-w
23 Zamora, M., Granell, M., Mampel, T. & Vinas, O. Adenine nucleotide translocase 3 (ANT3) overexpression induces apoptosis in cultured cells. *FEBS Lett.* **563**, 155-160 (2004). https://doi.org:10.1016/S0014-5793(04)00293-5
24 Michailidou, K. *et al.* Large-scale genotyping identifies 41 new loci associated with breast cancer risk. *Nat. Genet.* **45**, 353-361, 361e351-352 (2013). https://doi.org:10.1038/ng.2563
25 Boczonadi, V. *et al.* Mitochondrial oxodicarboxylate carrier deficiency is associated with mitochondrial DNA depletion and spinal muscular atrophy-like disease. *Genet. Med.* **20**, 1224-1235 (2018). https://doi.org:10.1038/gim.2017.251
26 Rosenthal, E. A. *et al.* Joint linkage and association analysis with exome sequence data implicates SLC25A40 in hypertriglyceridemia. *Am. J. Hum. Genet.* **93**, 1035-1045 (2013). https://doi.org:10.1016/j.ajhg.2013.10.019
27 Dupont, P. Y. & Stepien, G. Computational analysis of the transcriptional regulation of the adenine nucleotide translocator isoform 4 gene and its role in spermatozoid glycolytic metabolism. *Gene* **487**, 38-45 (2011). https://doi.org:10.1016/j.gene.2011.07.024
28 Del Arco, A. Novel variants of human SCaMC-3, an isoform of the ATP-Mg/P(i) mitochondrial carrier, generated by alternative splicing from 3'-flanking transposable elements. *Biochem. J.* **389**, 647-655 (2005). https://doi.org:10.1042/BJ20050283

29 Fiermonte, G., Paradies, E., Todisco, S., Marobbio, C. M. & Palmieri, F. A novel member of solute carrier family 25 (SLC25A42) is a transporter of coenzyme A and adenosine 3',5'-diphosphate in human mitochondria. *J. Biol. Chem.* **284**, 18152-18159 (2009). https://doi.org:10.1074/jbc.M109.014118
30 Almannai, M. *et al.* Expanding the phenotype of SLC25A42-associated mitochondrial encephalomyopathy. *Clin. Genet.* **93**, 1097-1102 (2018). https://doi.org:10.1111/cge.13210
31 Takeda, Y. & Dai, P. Chronic Fatty Acid Depletion Induces Uncoupling Protein 1 (UCP1) Expression to Coordinate Mitochondrial Inducible Proton Leak in a Human-Brown-Adipocyte Model. *Cells* **11** (2022). https://doi.org:10.3390/cells11132038
32 Farrow, E. *et al.* SLC25A24 gene methylation and gray matter volume in females with and without conduct disorder: an exploratory epigenetic neuroimaging study. *Transl Psychiatry* **11**, 492 (2021). https://doi.org:10.1038/s41398-021-01609-y
33 Yoneshiro, T. *et al.* BCAA catabolism in brown fat controls energy homeostasis through SLC25A44. *Nature* **572**, 614-619 (2019). https://doi.org:10.1038/s41586-019-1503-x
34 Stanzione, R. *et al.* Uncoupling Protein 2 as a Pathogenic Determinant and Therapeutic Target in Cardiovascular and Metabolic Diseases. *Curr. Neuropharmacol.* **20**, 662-674 (2022). https://doi.org:10.2174/1570159X19666210421094204
35 Abraham, C. G. *et al.* DeltaNp63alpha Suppresses TGFB2 Expression and RHOA Activity to Drive Cell Proliferation in Squamous Cell Carcinomas. *Cell Rep.* **24**, 3224-3236 (2018). https://doi.org:10.1016/j.celrep.2018.08.058
36 Braun, N. *et al.* UCP-3 uncoupling protein confers hypoxia resistance to renal epithelial cells and is upregulated in renal cell carcinoma. *Sci. Rep.* **5**, 13450 (2015). https://doi.org:10.1038/srep13450
37 Jabalameli, M. R. *et al.* Exome sequencing identifies a disease variant of the mitochondrial ATP-Mg/Pi carrier SLC25A25 in two families with kidney stones. *Mol Genet Genomic Med* **9**, e1749 (2021). https://doi.org:10.1002/mgg3.1749
38 Bitetto, G. *et al.* SLC25A46 mutations in patients with Parkinson's Disease and optic atrophy. *Parkinsonism Relat. Disord.* **74**, 1-5 (2020). https://doi.org:10.1016/j.parkreldis.2020.03.018
39 Zhou, X., Paredes, J. A., Krishnan, S., Curbo, S. & Karlsson, A. The mitochondrial carrier SLC25A10 regulates cancer cell growth. *Oncotarget* **6**, 9271-9283 (2015). https://doi.org:10.18632/oncotarget.3375
40 Haitina, T., Lindblom, J., Renstrom, T. & Fredriksson, R. Fourteen novel human members of mitochondrial solute carrier family 25 (SLC25) widely expressed in the central nervous system. *Genomics* **88**, 779-790 (2006). https://doi.org:10.1016/j.ygeno.2006.06.016
41 Tan, M. G., Ooi, L. L., Aw, S. E. & Hui, K. M. Cloning and identification of hepatocellular carcinoma down-regulated mitochondrial carrier protein, a novel liver-specific uncoupling protein. *J. Biol. Chem.* **279**, 45235-45244 (2004). https://doi.org:10.1074/jbc.M403683200
42 Buffet, A. *et al.* Germline Mutations in the Mitochondrial 2-Oxoglutarate/Malate Carrier SLC25A11 Gene Confer a Predisposition to Metastatic Paragangliomas. *Cancer Res.* **78**, 1914-1922 (2018). https://doi.org:10.1158/0008-5472.CAN-17-2463
43 Ji, Y. *et al.* Identification and characterization of novel compound variants in SLC25A26 associated with combined oxidative phosphorylation deficiency 28. *Gene* **804**, 145891 (2021). https://doi.org:10.1016/j.gene.2021.145891
44 Menga, A. *et al.* SLC25A26 overexpression impairs cell function via mtDNA hypermethylation and rewiring of methyl metabolism. *FEBS J* **284**, 967-984 (2017). https://doi.org:10.1111/febs.14028
45 Lu, H. *et al.* Cell cycle-dependent phosphorylation regulates RECQL4 pathway choice and ubiquitination in DNA double-strand break repair. *Nat Commun* **8**, 2039 (2017). https://doi.org:10.1038/s41467-017-02146-3
46 Aoki, Y. & Cortese, S. Mitochondrial Aspartate/Glutamate Carrier SLC25A12 and Autism Spectrum Disorder: a Meta-Analysis. *Mol. Neurobiol.* **53**, 1579-1588 (2016). https://doi.org:10.1007/s12035-015-9116-3
47 Montesanto, A. *et al.* Uncoupling protein 4 (UCP4) gene variability in neurodegenerative disorders: further evidence of association in Frontotemporal dementia. *Aging (Albany N. Y.)* **10**, 3283-3293 (2018). https://doi.org:10.18632/aging.101632
48 Chen, G., Mo, S. & Yuan, D. Upregulation Mitochondrial Carrier 1 (MTCH1) Is Associated with Cell Proliferation, Invasion, and Migration of Liver Hepatocellular Carcinoma. *Biomed Res Int* **2021**, 9911784 (2021). https://doi.org:10.1155/2021/9911784
49 Rabinovich, S. *et al.* The mitochondrial carrier Citrin plays a role in regulating cellular energy during carcinogenesis. *Oncogene* **39**, 164-175 (2020). https://doi.org:10.1038/s41388-019-0976-2
50 Hung, H. I., Schwartz, J. M., Maldonado, E. N., Lemasters, J. J. & Nieminen, A. L. Mitoferrin-2-dependent mitochondrial iron uptake sensitizes human head and neck squamous carcinoma cells to photodynamic therapy. *J. Biol. Chem.* **288**, 677-686 (2013). https://doi.org:10.1074/jbc.M112.422667

51 Labbe, K. *et al.* The modified mitochondrial outer membrane carrier MTCH2 links mitochondrial fusion to lipogenesis. *J. Cell Biol.* **220** (2021). https://doi.org:10.1083/jcb.202103122

52 Ramsden, D. B. *et al.* Human neuronal uncoupling proteins 4 and 5 (UCP4 and UCP5): structural properties, regulation, and physiological role in protection against oxidative stress and mitochondrial dysfunction. *Brain Behav* **2**, 468-478 (2012). https://doi.org:10.1002/brb3.55

53 Phillips, J. *et al.* Strong correlation of ferrochelatase enzymatic activity with Mitoferrin-1 mRNA in lymphoblasts of patients with protoporphyria. *Mol. Genet. Metab.* **128**, 391-395 (2019). https://doi.org:10.1016/j.ymgme.2018.10.005

54 Kory, N. *et al.* MCART1/SLC25A51 is required for mitochondrial NAD transport. *Sci Adv* **6** (2020). https://doi.org:10.1126/sciadv.abe5310

55 Haguenauer, A. *et al.* A new renal mitochondrial carrier, KMCP1, is up-regulated during tubular cell regeneration and induction of antioxidant enzymes. *J. Biol. Chem.* **280**, 22036-22043 (2005). https://doi.org:10.1074/jbc.M412136200

56 Zhang, H. *et al.* Elevated mitochondrial SLC25A29 in cancer modulates metabolic status by increasing mitochondria-derived nitric oxide. *Oncogene* **37**, 2545-2558 (2018). https://doi.org:10.1038/s41388-018-0139-x

57 Porcelli, V., Fiermonte, G., Longo, A. & Palmieri, F. The human gene SLC25A29, of solute carrier family 25, encodes a mitochondrial transporter of basic amino acids. *J. Biol. Chem.* **289**, 13374-13384 (2014). https://doi.org:10.1074/jbc.M114.547448

58 Prohl, C. *et al.* The yeast mitochondrial carrier Leu5p and its human homologue Graves' disease protein are required for accumulation of coenzyme A in the matrix. *Mol. Cell. Biol.* **21**, 1089-1097 (2001). https://doi.org:10.1128/MCB.21.4.1089-1097.2001

59 Al Shamsi, B., Al Murshedi, F., Al Habsi, A. & Al-Thihli, K. Hypoketotic hypoglycemia without neuromuscular complications in patients with SLC25A32 deficiency. *Eur. J. Hum. Genet.* **30**, 976-979 (2022). https://doi.org:10.1038/s41431-021-00995-7

60 Huttlin, E. L. *et al.* Architecture of the human interactome defines protein communities and disease networks. *Nature* **545**, 505-509 (2017). https://doi.org:10.1038/nature22366

Table S7. L10 and S10 SLC25 transporters for kidney and colon cell lines

L10						S10					
Kidney				Colon		Kidney				Colon	
RXF-393	CAKI-1	786-O	A498	HCT-116	HT-29	RXF-393	CAKI-1	786-O	A498	HCT-116	HT-29
A32	A32	A24	A43	A21	A22	A47	A48	A33	A48	A18	A21
A43	A22	A32	A22	A22	A8	A35	A42	A48	A25	A34	A34
A24	A14	A43	A32	A30	A32	A27	A29	A18	A4	A25	A48
A14	A19	A22	A23	A32	A28	A48	A27	A27	A34	A24	A20
A23	A43	A40	A14	A19	A40	A25	A34	A25	A29	A45	A4
A37	A24	A3	A39	A39	A19	A34	A18	A34	A33	A20	A9
A40	A39	A14	A46	A36	A15	A20	A47	A4	A42	A8	A24
A22	A36	A1	A24	A17	A17	A4	A25	A45	A27	A13	A45
A36	A15	A17	A41	A33	A39	A8	A20	A30	A20	A44	A41
A1	A26	A19	A10	A26	A30	A45	A21	A20	A5	A16	A16

Note: transcript ratios were calculated by dividing the average of n=2 qRT-PCR values from the CCLs by the average qRT-PCR values from the seven uninvolved ccRcc tissues or the six (no matched uninvolved tissue for 3463-3T) uninvolved CRC tissues.
